# The Mousterian in North-Western Tuscany: new data from the Piano di Mommio sites

**DOI:** 10.12688/openreseurope.17018.1

**Published:** 2024-02-22

**Authors:** Jacopo Gennai

**Affiliations:** 1 Civilisations and Forms of Knowledge, University of Pisa, Pisa, Tuscany, 56126, Italy

**Keywords:** Mousterian, Neanderthals, archaeological excavation, stratigraphy, Italy, fieldnotes

## Abstract

**Background:**

The Mousterian technocomplex is commonly associated with Neanderthals and therefore serves as a proxy for their presence across Europe. Stratified archaeological sites are the most informative because they can yield information about artefacts' spatial distribution and dating. Only a few of the Mousterian sites in Tuscany (Italy) met these conditions and most of these sites are concentrated in the North-Western region, with three specific sites situated in proximity to the village of Piano di Mommio, on the slopes of a small river canyon. Nevertheless, research on the sites stopped early on due to their small extent and complete excavation, which does not allow for additional fieldwork.

**Methods:**

This article presents previously unpublished field notes, reports, and images, which are then correlated with recent archaeological surveys.

**Results:**

This combination of historical and contemporary data aims to provide a more detailed understanding of the context in which the assemblages at these sites were found. The insights gained from this research shed light on the arrangement and positioning of artefacts at these locations, offering valuable information to guide future investigations on the assemblages.

**Conclusions:**

By enhancing our knowledge of Neanderthal presence in this region through this interdisciplinary approach, this study contributes to a better understanding of the Mousterian culture and the broader narrative of human prehistory in Italy. It underscores the importance of integrating historical field data with modern archaeological techniques to advance our understanding of human history.

## Plain language summary

Buca della Iena, Grotta del Capriolo and Grotta del Cervo are three small sites in the hills next to the town of Viareggio (Tuscany, Italy). Buca della Iena and Grotta del Capriolo were investigated in the late 1960s, while Grotta del Cervo, despite being located next to Buca della Iena, was discovered and investigated much later during the early 1990s. The three sites represent some of the few stratified archaeological sites revealing Mousterian artefacts in Tuscany. The Mousterian is the material culture generally associated with Neanderthals, the indigenous European extinct hominin species that preceded Homo sapiens. Stratified sites are pivotal in Archaeology to discern spatial and chronological relationships. By establishing what lies above or beneath archaeologists can interpret what is younger and what is older, also by dating material associated with the artefacts we can obtain an absolute date for the artefacts and the human occupation. Stratigraphical contexts in the three sites are hindered by decades of lack of research, which meant the loss of information about excavation methods and therefore stratigraphical relationships of the artefacts. The article presents the effort to publish all resources still available and draw some new interpretations about how the artefacts might have been found at the sites. Thanks to this the artefacts can be investigated again and shed new light on Neanderthals behaviour in Tuscany and Italy.

## Introduction

### The Mousterian industries

The Mousterian, a lithic industry primarily characterised by flake production, is associated with Neanderthals in Europe ( Higham *et al.*, 2014). Initially defined by Gabriel de Mortillet in 1873, the Mousterian became a fundamental term within the field of Prehistoric Archaeology as the discipline advanced, ultimately becoming often interchangeable with the term Middle Palaeolithic in Europe ( Depaepe, 2020; Monnier, 2006). Spanning from approximately 300/250 thousand years Before the Present (ka BP) to around 40 thousand years calibrated Before the Present (cal BP) ( Depaepe, 2020; Higham *et al.*, 2014; Richter, 2011), the European Middle Palaeolithic period closely aligns with the emergence and evolution of the Neanderthal species ( Arsuaga *et al.*, 2014; Romagnoli *et al.*, 2022). At the core of the Mousterian lies the adoption of predeterminate flaking methods. These facilitated the controlled shaping of end products and, in specific instances, the categorisation of products into distinct technological roles—core-shaping flakes, forming the necessary convexities, and end-products, crafted from these convexities ( Moncel *et al.*, 2020; Moncel *et al.*, 2012; Richter, 2011; Soressi & Geneste, 2011). François Bordes sought to systematise the Mousterian and the broader Middle Palaeolithic framework, resulting in the creation of the first typological list for Lower and Middle Palaeolithic artefacts in 1961, which is largely adopted until nowadays ( Bordes, 2000). This taxonomy contains also non-retouched types, for example, Levallois products. Subsequently, the concept of chaîne opératoire and the integration of lithic technology into lithic artefacts’ studies contributed to a clearer elucidation of the Levallois flaking process ( Audouze & Karlin, 2017; Boëda, 1994). The emergence of the Levallois method is as early as MIS 12 to MIS 9, with several sites yielding artefacts characteristic of this knapping strategy ( Carmignani *et al.*, 2017; Hérisson & Soriano, 2020; Moncel *et al.*, 2020). From MIS 8 onwards the method is spread throughout the whole of Europe ( Delagnes *et al.*, 2007; Delagnes & Meignen, 2006; Faivre *et al.*, 2017). In addition, to the Levallois method, the Mousterian assemblages revealed other flaking techniques, such as the Quina and the Discoid methods ( Boëda, 1993; Boëda *et al.*, 1990; Bourguignon, 1997; Delagnes *et al.*, 2007; Peresani, 2003). It is worth noting that the chaîne opératoires are not inherently exclusive, with instances of cross-method integration on the same core or branching out in a technological continuum ( Bourguignon *et al.*, 2004; Bustos-Pérez *et al.*, 2023; Çep *et al.*, 2021; Mathias & Bourguignon, 2020). Since the initial work of Bordes ( Bordes, 1953), archaeologists have endeavoured to identify shared typological and technological traits within technocomplexes, which underlie Palaeolithic societies and possibly indicate phylogenetic connections ( Reynolds & Riede, 2019; Riede *et al.*, 2020). For the European Middle Palaeolithic, the prime focus of this endeavour has centred on southwestern France due to the abundance of sites across different periods ( Delagnes & Rendu, 2011; Faivre *et al.*, 2017; Jaubert *et al.*, 2011). However, the identified technocomplexes have been subject to diverse debates. Binford, for instance, favoured a functional interpretation over Bordes' cultural perspective ( Binford & Binford, 1966; Binford & Binford, 1969). Subsequently, as researchers scrutinised the concept of technocomplexes within the context of global variation, questions arose concerning their significance ( Dibble, 1991; Monnier & Missal, 2014; Shea, 2014). Even assuming that technocomplexes are real societal entities, the cultural sequence in southwestern France has been a subject of intense discussion. For instance, Bordes and subsequent scholars recognised a connection between the Mousterian of Acheulean Tradition – A (MTA – A), the Mousterian of Acheulean Tradition – B (MTA – B), and the Chatelperronian, as these assemblages often were found in this stratigraphical order and had insights of gradual techno-typological evolution ( Roussel & Soressi, 2014). More recently, this lineage has been re-evaluated considering the potential non-existence of the MTA – B (conceived from unrecognised lithic artefacts mixing in palimpsests) and the identification of distinct final Mousterian technocomplexes between MTA – A and the Chatelperronian ( Gravina & Discamps, 2015; Jaubert *et al.*, 2011).

### The Mousterian sites in Tuscany

**Figure 1.**
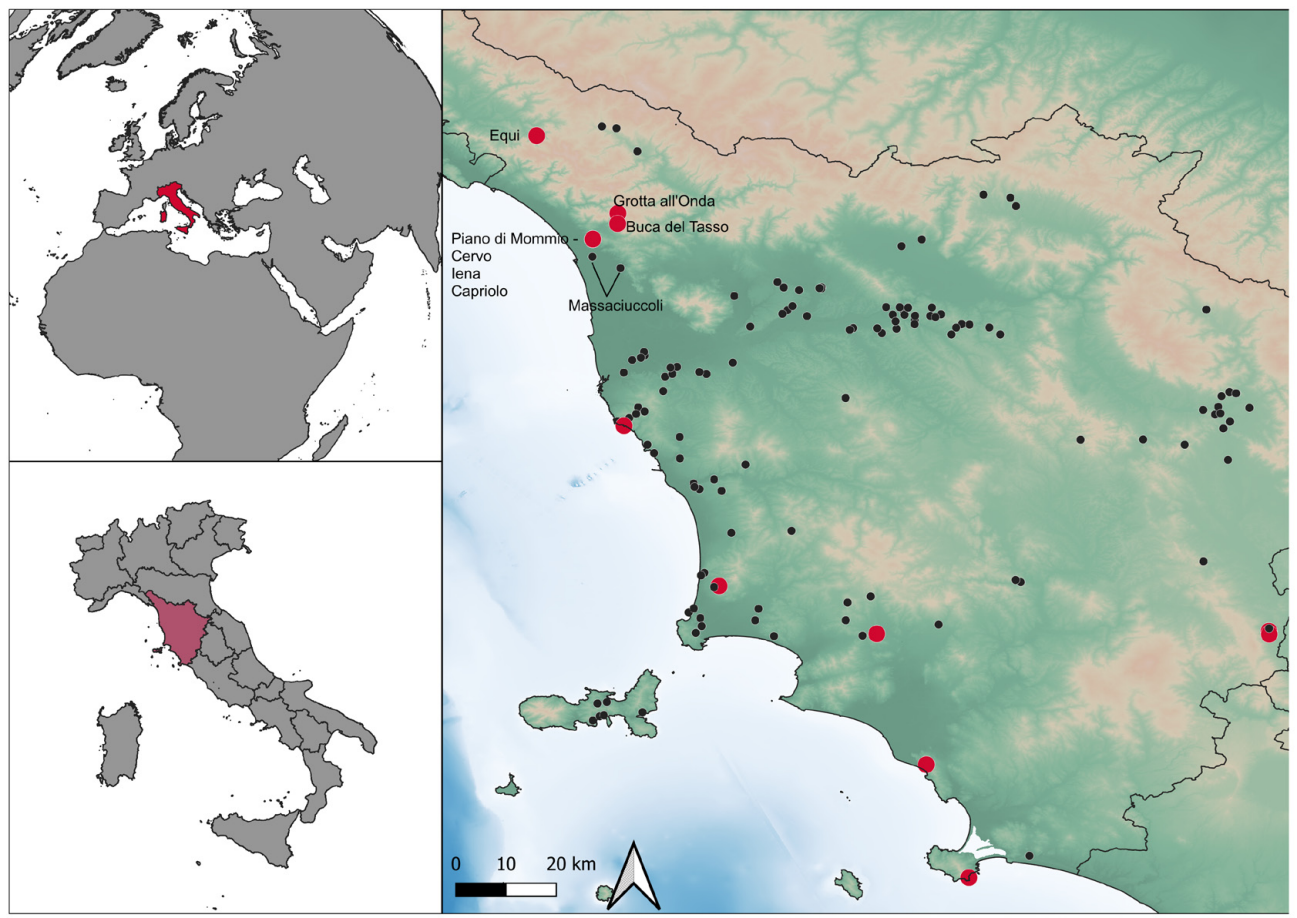
Location of Mousterian sites in Tuscany and sites mentioned in the text. Red: stratified sites.

The Mousterian is distributed across the whole Italian Peninsula, revealing clusters of sites, particularly referring to stratified assemblages ( Aureli & Ronchitelli, 2018; Palma di Cesnola, 2001). Notably, the northwestern area of Tuscany (Italy – Figure 1) stands as one of the prominent clusters of stratified sites ( Gennai, 2023). Most of the discoveries in the area date to the earliest endeavours of Prehistoric Archaeology in Italy ( Blanc *et al.*, 1935; Branchini, 1928; De Stefani, 1915; Graziosi, 1944; Mochi, 1915; Palma di Cesnola, 1970; Regnoli, 1867; Rellini, 1916). In order of discovery, the sites yielding Middle Palaeolithic assemblages that emerged during this pioneering era are Grotta all’Onda, Grotta and Tecchia d’Equi, and Buca del Tasso.

Grotta all’Onda (located near Casoli, Camaiore, Italy) stands as one of the earliest Prehistoric sites to be surveyed and excavated in Italy, a pursuit initiated during the latter half of the 19th century by C. Regnoli. He was notably captivated by the large and conveniently accessible cavern, leading to exploratory test pits dispersed throughout the cave's extent. He dug a trench in the western side of the cave, a zone less affected by cave roof collapse boulders. Regnoli's trench reached a depth of 34 cm stopping when a hard surface, interpreted as the bedrock, was hit: no stratigraphical profile is available anymore. The findings revealed vestiges of the Neolithic and post-Neolithic epochs ( Regnoli, 1867). These preliminary findings sparked a rather unstructured examination of the site, which continued yielding artefacts associated with the Neolithic and Metal Ages ( Tonini, 1886). In 1914, A. Mochi and R. Schiff-Giorgini embarked upon the first systematic excavation of Grotta all’Onda. Their efforts were intertwined within a broader survey initiative spanning the surrounding district of the southern Apuan Alps ( Mochi, 1927). They published a full report of their activities ( Mochi, 1915; Mochi & Schiff-Giorgini, 1915). Their investigation encompassed a more substantial trench (measuring 18,5 meters in length and 4 – 8 meters in width), still in the western cave sector and involving the cave mouth area. They recognised that the Regnoli’s bedrock was a thick flowstone, and they exposed a 4.5-metre stratigraphic sequence, revealing the presence of multiple flowstones. The initial flowstone lays beneath the Neolithic layer, followed by a substantial clay deposit (Layer 3). Layer 3, in turn, exhibited two distinctive darker horizons containing Pleistocene fauna, including cave bear remains ( *Ursus spelaeus*). The first of these dark horizons (Foyer B) lacked artefacts, whereas the deeper one (Foyer C) yielded lithic artefacts attributed to the Upper Palaeolithic (Aurignacian). Another flowstone separated Foyer C from a deeper, more humid clay layer containing more Pleistocene fauna (Layer 5 – Foyer D). This latter layer yielded a small assemblage of Middle Palaeolithic (Mousterian) lithic artefacts. Foyer D rested upon yet another flowstone, which was coating a substantial bedrock of boulders. P. Graziosi, L. Cardini, and N. Puccioni re-investigated the site in 1931 ( Graziosi, 1944; Graziosi, 1934; Puccioni, 1933). They opened a new trench between the 1914 excavations and the western cave wall, keeping intact deposits next to the wall as future reference. Their examination corroborated the lithostratigraphic revelations of 1914, up to the second dark horizon of Layer 3. However, beneath this level, the neat segregation of Layer 5 from Layer 3 could not be confirmed. Instead, they proposed that these layers are constituent components of the same anthropic horizon, which appears in lenses disrupted by roof collapse and the formation of flowstones. They found few artefacts in Layers 3 and 5, attributed to the Middle Palaeolithic, prompting a re-evaluation of the 1914 findings, and not supporting the presence of the Upper Palaeolithic. Thus, the entire interval spanning the initial to the ultimate flowstone was deduced to belong to the Middle Palaeolithic, with Foyer D revealed as an integral aspect of Foyer C. Furthermore, they excavated 4,5 m beneath the flowstone of Layer 5, finding no artefacts. A subsequent sampling of the Layer 5 flowstone secured a radiometric date of 39.3 ± 3.2 ka ( Fornaca-Rinaldi & Radmilli, 1968). Following a prolonged hiatus, research endeavours resumed in 1996 under the leadership of S. Campetti, ending in 2005 ( Berton *et al*., 2005; Campetti *et al*., 2001). Campetti's research aimed to further unravel the lithostratigraphy and chronological context of the site, extending their excavations to encompass the unexplored portion against the cave's western wall. Their meticulous efforts were instrumental in enhancing the comprehension of the site's sedimentary layers and their temporal sequence. Delving deeper into the stratigraphy, they revealed the presence of the First Stalagmitic Level, which acted as the start of the archaeological sequence. Immediately atop this, a slender layer of light clay was identified, followed by a substantial deposit of brecciated material (Layer 1). Nestled within Layer 1, a dark reddish stratum was discerned, corresponding to the Foyer C of 1931. Overlying the occupation layer, a sterile clay layer (Layer 2) emerged, succeeded by a higher content of clast and limestone (Layer 3). This uppermost layer (Layer 4) was in turn capped by the Second Stalagmitic Level, marking the transition to the Neolithic deposit situated at the apex of the stratigraphic succession (Layer 5). The retrieval of archaeological materials yielded varying quantities across the layers, with the Middle Palaeolithic assemblage (Layer 1) containing a relatively more abundant number of artefacts (18 in total). Layer 3's sparse assortment, on the other hand, suggested a possible association with the Upper Palaeolithic. A significant difference was made with the discovery of an intact hearth with abundant lithic artefacts, positioned adjacent to the modern entrance of the cave. Ascribed to the late Upper Palaeolithic (Epigravettian) period, this find emerged as a notable update to the site ( Berton *et al.*, 2003b). In tandem with their renewed excavation efforts, radiometric dating endeavours were also undertaken. The First Stalagmitic Level, presumed to be the deeper flowstone observed in 1914 documentation, was re-dated using the 230Th/232Th method, yielding an age of 174.0 ± 8.2 ka BP. This substantially predates the previous dating from 1968, while, the Second Stalagmitic Level was ascertained to be 10.7 ± 0.2 ka BP, reaffirming its role as a demarcation between the Pleistocene and the Holocene ( Berton *et al*., 2003a). A likely explanation for the discrepancy between 1968 and the latter measurement of the First Stalagmitic Level is that 1968 investigations sampled a localised calcite accumulation within the Foyer C, nowadays destroyed ( Berton *et al*., 2003a). Radiocarbon dating conducted on bones sourced from the 1999 Layer 1 yielded results more closely aligned with the earlier dating of the flowstone: 37,139 ± 530 (68.2% probability: 42,076 – 41,256 cal BP; 95.4% probability: 42,462 - 40,724 cal BP) and 36,996 ± 565 (68.2% probability: 42,016 – 41,096 cal BP; 95.4% probability: 42,402 – 40,498 cal BP) ( Moroni *et al*., 2019). However, the bones exhibited no anthropogenic modification, and the presence of hibernating cave bear remains within Layer 1 suggested human presence at the site was sporadic ( Molara, 2009).

The Grotta and Tecchia d’Equi (Equi Terme, Italy), an important archaeological and paleontological site in Tuscany, consists of two contiguous parts: the Tecchia (a shelter) and the Grotta (inner cave). In 1909, fossilized bones were found in the Tecchia, prompting systematic excavations starting in 1911 led by C. De Stefani ( De Stefani, 1916a; De Stefani, 1916b; De Stefani, 1916c; De Stefani, 1915). De Stefani’s campaigns continued until 1917, revealing the Grotta, until then covered by sediment ( De Stefani, 1923; De Stefani, 1916b; De Stefani, 1916a; De Stefani, 1916c; Rellini, 1924; Rellini, 1916). The Tecchia showed traces of occupation and use during historical times, while the Grotta, focus of the De Stefani excavations, showed Prehistoric settlements ( Bigagli *et al*., 2018; De Stefani, 1916a). The entrance of the cave started at about 1.3 m below the Tecchia floor level (the origin of all the De Stefani’s depth measurements). The stratigraphical sequence of the cave unveiled an intricate alternation between limestone breccias and thin layers of fine reddish sediment, which turned yellowish at the end of the sequence. The sequence was mainly horizontal at the cave mouth while in the inner cave sediment accumulated mostly in lenses ( De Stefani, 1916c). Of paramount significance was the discovery of a distinctive archaeological horizon, found roughly midway through the excavation at a depth of 3.75 m. This layer, spanning 0.1 to 0.3 m in thickness, yielded a diverse assemblage of Pleistocene fauna, notably the cave bear ( *Ursus spelaeus*), alongside traces of human presence. These traces included fragmentary human remains, pottery fragments, and lithic artefacts potentially originating from the Neolithic or Metal Ages. In addition, in a lower stratigraphical position, Mousterian lithic artefacts ( De Stefani, 1916b; De Stefani, 1916b; De Stefani, 1916a; Ghezzo *et al.*, 2014; Rellini, 1924). The archaeological campaigns of 1916 and 1917 unearthed an additional layer located approximately 5 meters below the Tecchia floor. This lower layer yielded further Mousterian artefacts ( De Stefani, 1923; De Stefani, 1917; Ghezzo *et al.*, 2014; Rellini, 1924; Rellini, 1916). De Stefani noticed also that the cave continued deeper, but abandoned the investigation after consideration that the stratigraphical sequence and the mostly palaeontological content did not differ from areas already excavated ( De Stefani, 1923; De Stefani, 1917). U. Rellini continued the site's exploration in 1919, focusing his investigations on the cave's entrance. His work confirmed the stratigraphic sequence reported by De Stefani, affirming the presence of distinct layers separated by compact breccia accumulations ( Rellini, 1924). A clear difference emerged, with the first archaeological layer spanning the entire cave, while the second lower layer predominated on the cave's northern wall. The retrieved artefacts echoed earlier findings, featuring undiagnostic lithic artefacts and rudimentary pottery shards ( Rellini, 1924). In subsequent years, I. Branchini undertook the task of analysing the whole archaeological assemblage from the site. Her study concluded that most artefacts resonated with the Palaeolithic, particularly the Mousterian, alongside Neolithic artefacts, lithic ornaments, and pottery shards ( Branchini, 1928). In 1931, the Italian Institute of Human Palaeontology launched a new investigation of Prehistoric sites in the Apuan Alps, and the Tecchia d’Equi was selected for further investigation ( Blanc *et al.*, 1935; Graziosi, 1934). Considering the cave deposit seemed completely removed, G.A. Blanc focused on the Tecchia, where the deposit was untouched. They described a stratigraphy characterised by an upper horizon of reddish clay and an underlying stratum of greyish sand ( Blanc *et al.*, 1935). Importantly, only the upper horizon yielded artefacts, originating from distinct stratigraphic lenses (B and C) ( Blanc *et al.*, 1935). While all the artefacts were attributed to the Mousterian, they remained unpublished and are now lost ( Blanc *et al.*, 1935; Palma di Cesnola, 1970). Prehistoric research was halted at the site, except for the inclusion of the site in the Caves’ Registrar in the ‘50s, during this work new *in situ* deposit in the inner cave was revealed and prompted new research during the ‘70s and the ‘80s ( Ambrosi, 1959; Ambrosi & Feola, 1951; Graziosi, 1969; Graziosi & Guerri, 1972; Guerri, 1982; Guerri, 1980). These spatially limited excavations mostly revealed reworked deposits, due to looters’ digs, with some sparse Mousterian artefacts and cave bear bones ( Graziosi & Guerri, 1972). In 1980, M. Guerri started a test pit in an unexcavated part of the Tecchia and interpreted this area reworked as well, except for a small strip next to the wall ( Guerri, 1980). In 1982, she dug a test pit in the deeper cave left untouched by De Stefani, finding a flowstone capping brecciated sediments with animal bones but no artefacts ( Guerri, 1982). After a long hiatus, research started again in 2009, when the Soprintendenza per i Beni Archeologici della Toscana found untouched deposits both in the Tecchia and in the Grotta ( Iardella *et al.*, 2012). The excavation focused on the western wall of the Tecchia and revealed a deposit characterised by loess sediments. Within the upper portion of the deposit, Mousterian artefacts, fauna remains, and dispersed charcoals were discovered, often associated with detached boulders from the Tecchia's roof ( Iardella *et al.*, 2012). However, this portion of the deposit up to the floor level, exhibited varying degrees of reworking ( Iardella *et al.*, 2012). Subsequent investigations in 2012 extended to the outermost part of the Grotta (Sala 1), revealing yet another *in situ* deposit. This deposit was defined by loess sediment lenses intermingled with detached boulders and breccias from the cave roof. Mousterian artefacts and charcoal were also uncovered within this context, dated to 43700 ± 1900 BP and 44000 ± 2200 BP, effectively aligning the deposit with MIS 3 ( Bigagli *et al.*, 2018; Bigagli *et al.*, 2013). Further developments in 2014 involved the re-excavation of the transitional zone connecting the exterior section of the cave (Sala 1) to the previously explored interior area from the 1980s. This decision was prompted by the recognition of additional *in situ* deposits. The sediment composition in this area retained its loess nature mixed with breccia debris, and the archaeological findings remained consistent with the Mousterian tradition. Nonetheless, dating efforts for these levels, along with those from the 2010 Tecchia investigations, revealed dates exceeding 45 ka BP, providing evidence of an earlier phase of Palaeolithic occupation ( Bigagli *et al.*, 2018; Palchetti, 2015).

Buca del Tasso (Metato, Camaiore, Italy) was discovered during an archaeological survey in 1919 ( Puccioni, 1922). Initially appearing as a shelter perched above a deep canyon, the site revealed its true nature when a small opening beneath the low roof led into a cave. Under the direction of N. Puccioni, excavations were promptly initiated and completed in 1922 through three archaeological campaigns that emptied the deposit both in the talus and in the small cave ( Puccioni, 1922). Initially, the focus was on the shelter and the talus adjacent to the canyon, revealing a stratigraphic sequence that reached approximately 3 – 3.4 meters (north-eastern wall). Despite some reworking attributed to fossorial animals like the badger ( *Meles meles*), the deposit's integrity remained preserved ( Puccioni, 1922). The stratigraphy from top to bottom comprised a thin flowstone, followed by a loose silty light brown layer (A) measuring around 0.7 meters in thickness. Subsequently, a compacted breccia layer (B) of similar thickness follows. Layer C, representing a clay layer, is further subdivided into components: a compact layer (C’), a roof collapse level hosting substantial limestone boulder (C’’), and ultimately another compact clay layer (C’’’) that rests upon the bedrock ( Puccioni, 1922). Within the cave, layer A reveals two distinct horizons—a darker upper layer and a reddish lower one—where the thickness of layer A diminishes with cave interior progression. Beneath layer A, layer B rests directly upon the bedrock ( Puccioni, 1922). Both Layer A and Layer C yielded Mousterian lithic artefacts. These assemblages are relatively modest: Layer A comprised a total of 58 artefacts, whereas Layer C contained 8 artefacts in total ( Palma di Cesnola, 1970; Puccioni, 1926; Puccioni, 1922). Notably, within the upper portion of Layer A, two human remains, femur fragments, were found. Of these remains, only one is currently recognised as a Neanderthal, attributed to a 7-9-year-old child. This constitutes the sole Neanderthal evidence in Tuscany ( Alciati *et al.*, 2005; Puccioni, 1922). Faunal evidence is notably present within Layers A and C, with Layer A displaying greater abundance. This fauna encompasses cold and alpine environments inhabited by creatures such as the marmot ( *Marmota marmota*), chamois ( *Rupicapra rupicapra*), and ibex ( *Capra ibex*). Additionally, the faunal record incorporates species adapted to more open and warm environments, including the rhino ( *Rhinocerus mercki*) and auroch ( *Bos taurus*). While carnivores are not abundant, the cave bear ( *Ursus spelaeus*) and some hyena ( *Hyeana crocuta spelea*) remains are present. Considering the faunal composition and the chronological framework provided by neighbouring sites such as Grotta all’Onda, Buca del Tasso is associated with the MIS 3 period ( Alciati *et al*., 2005).

Palaeolithic artefacts, among those Mousterian ones, occurred with the commercial sand extraction in various localities of the Massaciuccoli Lake (Viareggio – Massarosa, Lucca, Italy)( Blanc, 1937; Blanc, 1934; Fornaciari, 1966). Depending on the area of extraction, they could originate from 12 to 26 m depth, but ascertaining the real burial context is impossible at the current stage ( Blanc, 1934; Fornaciari, 1966).

**Figure 2.**
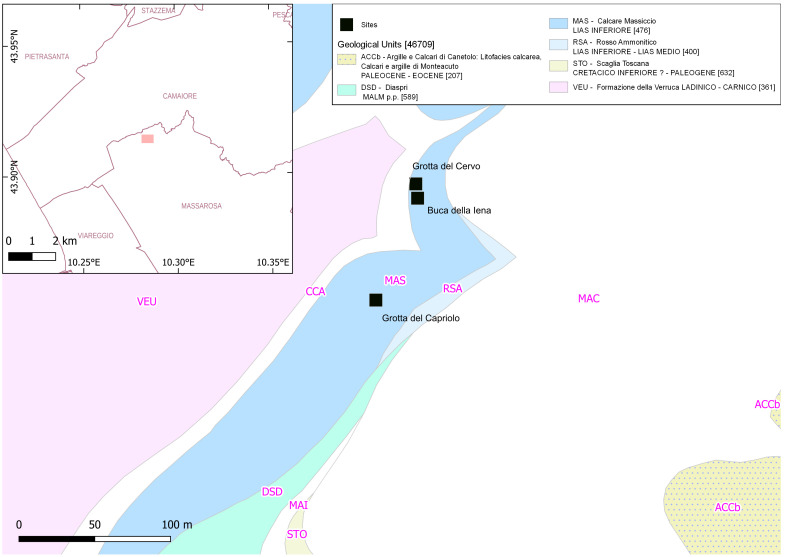
Location of Piano di Mommio sites within their geological context (DB geologico 1:10.000 Regione Toscana).

**Figure 3.**
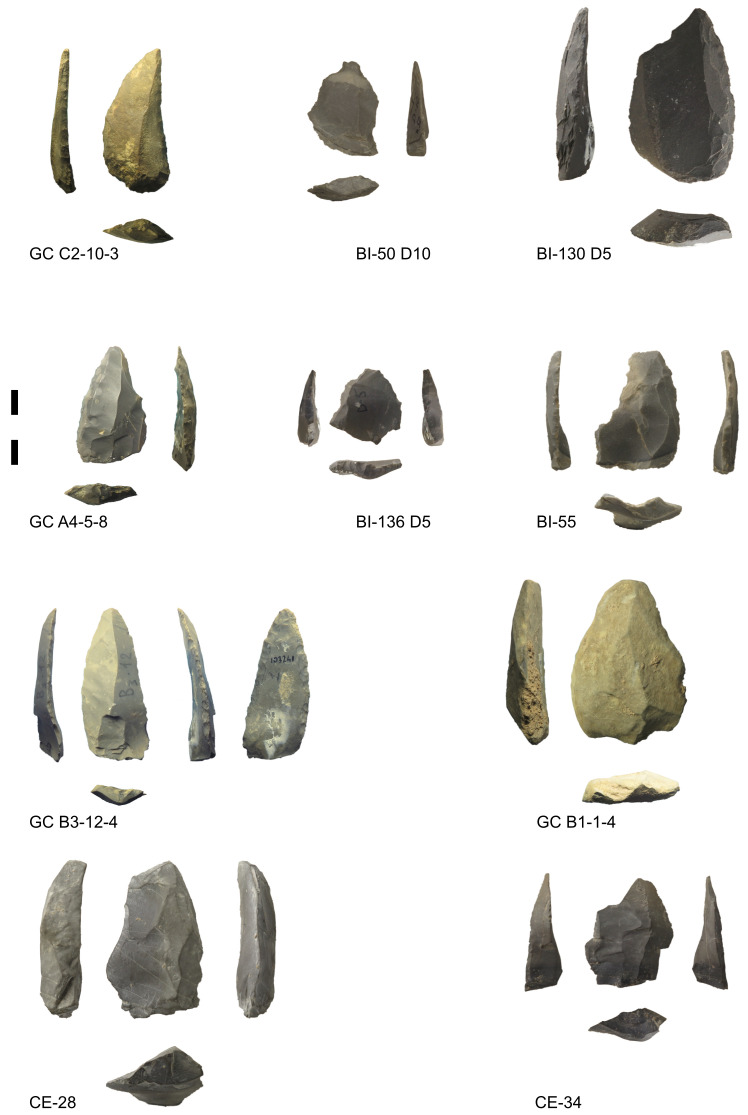
Some of the Mousterian artefacts from Buca della Iena (BI), Grotta del Capriolo (GC) and Grotta del Cervo (CE). Photos Jacopo Gennai.

The above-mentioned findings stimulated further research activities by a local group of amateur archaeologists (Gruppo Ricerche Preistoriche e Archeologiche “Alberto Carlo Blanc”) collaborating with the professor of Prehistoric Archaeology A.M. Radmilli. They focused on the limestone landscape next to Piano di Mommio (Massarosa, Lucca, Italy) ( Figure 2; Conti *et al.*, 2010; Gennai, 2023) leading to the discovery of multiple Prehistoric sites, among those two new Mousterian sites: Buca della Iena and Grotta del Capriolo ( Fornaciari, 1977; Fornaciari, 1966; Gennai, 2023; Pitti & Tozzi, 1971). Dating of a flowstone underlying the Buca della Iena assemblage provided a *terminus post quem* for the artefacts <41 ka BP ( Fornaca-Rinaldi & Radmilli, 1968), and the sites are generally believed to belong to the last Mousterian manifestations ( Figure 2; Figure 3; Gennai, 2023). Some 20 years later, the Grotta del Cervo site was added ( Cocchi, 1989; Cocchi-Genick, 1993; Cocchi-Genick, 1992; Cocchi-Genick, 1989; Gennai, 2023). These three new sites, given the proximity and the typological similarities, were aggregated with Tecchia d’Equi, Grotta all’Onda, and Buca del Tasso in the so-called “Alpine Mousterian”, rich in denticulates ( Cocchi-Genick, 1993; Palma di Cesnola, 1970; Pitti & Tozzi, 1971). More recently, the presence of denticulates was downplayed in favour of a more technological approach, but the sites are still considered largely contemporary, belonging to the MIS 3 and the last Neanderthals in the area ( Dini & Koehler, 2009). Despite the relatively small assemblages, short stratigraphical sequences and limited site extent, the sites are of vital importance to the understanding of the last Neanderthals’ survival in Central Italy due to the scarcity of stratified sites. Nevertheless, details are difficult to obtain because publications are dispersed in various notes and local papers in Italian.

## Methods

The research for the original documentation began with the primary goal of determining the stratigraphical provenance of the artefacts. These artefacts raised discrepancies with their published provenance details ( Fornaciari, 1966; Pitti & Tozzi, 1971). As a result, it became imperative to source the original documentation and ascertain the genuine stratigraphical origins of these artefacts. The investigation encompassed multiple research avenues.

Most of the original documentation or duplicates were procured through collaboration with the original excavators, specifically Prof. Gino Fornaciari and Prof. Carlo Tozzi. These documents included two profile sections of Buca della Iena, one profile section (Trench B), and the comprehensive plan of the 1968 Grotta del Capriolo excavations. In addition, they provided the Buca della Iena 1966 excavation field notes and the 1970 Grotta del Capriolo excavation field notes.

Concurrently, the literature search extended to local publications. The Soprintendenza Beni Archeologici e Paesaggistici (SABAP) Massa-Carrara/Lucca possessed additional resources, encompassing the Bulletin of Information and the Activities Diary of the local archaeologists' society, which had conducted excavations at Buca della Iena and Grotta del Capriolo. This source also provided the Grotta del Cervo 1989 post-excavation report, along with the site plan, profile sections, and the list of spits. Furthermore, the Museo Civico Archeologico e dell’Uomo “Carlo Alberto Blanc” in Viareggio housed photographs of the Grotta del Cervo excavation, in addition to the archaeological material.

Given the composite nature of the documentation, sites needed new surveys to compare with the documentation and address any gaps. This entailed multiple site visits to obtain fresh photographs, and coordinates, and to compare or integrate existing documentation. This process was particularly significant for Grotta del Cervo and Buca della Iena, which are both located on the same private property.

## Results

### Buca della Iena

**Figure 4.**
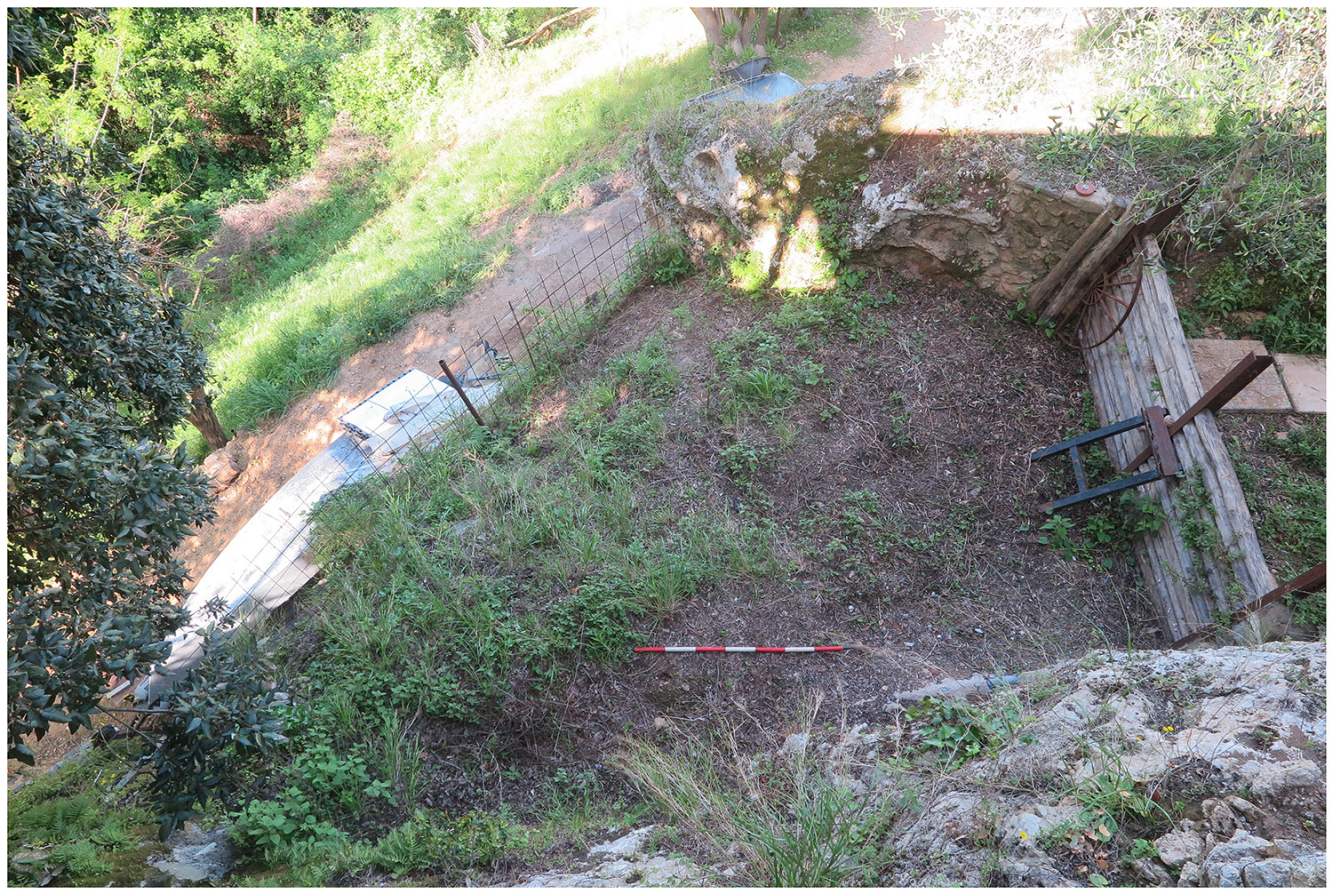
The terrace in front of the Buca della Iena. The limestone boulder ends it. Photo Jacopo Gennai.

**Figure 5.**
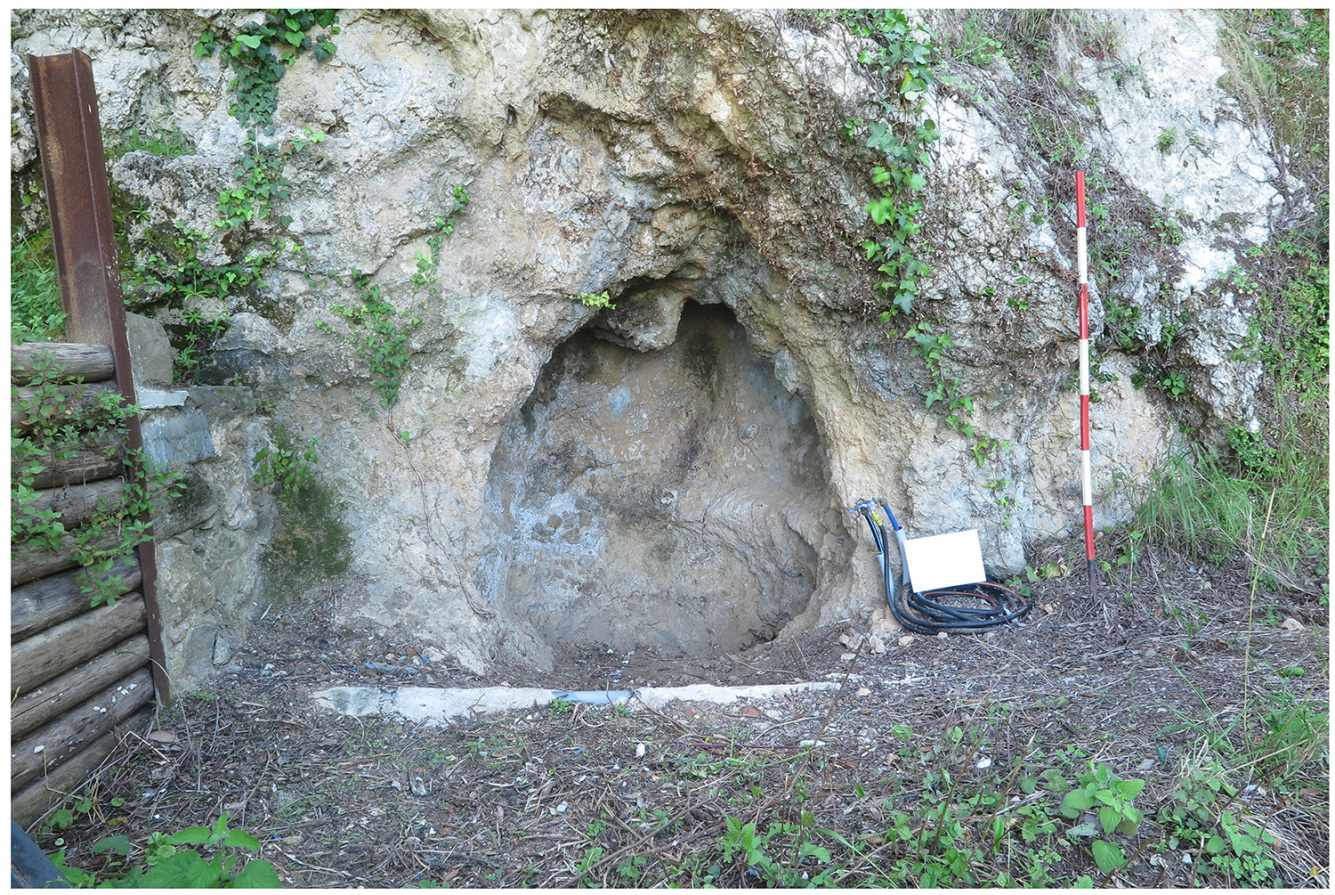
The Buca della Iena niche in the limestone wall. Photo Jacopo Gennai.

The site is located on a limestone outcrop which is isolated by two narrow canyons on the southern and northern sides, nowadays it faces west-southwest (43.9142645 N, 10.2850590 E; Figure 2; Gennai, 2023). The slope was modified by terracing to facilitate farming and one of these terraces took advantage of the extent between the limestone natural step and two limestone boulders ( Figure 4; Gennai, 2023). Nowadays the site consists only of an empty, small niche in the limestone wall, measuring around 2 m in length 1,7 m in width and 1,85 m maximum height ( Figure 5; Gennai, 2023).

The site was discovered on the 17th of May 1966 and interpreted as a collapsed cave. A small test pit yielded bones of Pleistocene fauna in light-coloured clay underneath the humic level. On the 28th of May, a second test pit was dug, next to the previous one, discovering analogous findings. The systematic excavation started on the 6th of July of that year and lasted until the 20th of July ( Gennai, 2023; Grifoni Cremonesi, 1971; Radmilli, 1966). The following year the Institute for Human Palaeontology of the University of Pisa sampled the remaining stratigraphical sequence to ascertain the sedimentological sequence ( Gennai, 2023; Pitti & Tozzi, 1971; Radmilli, 1967). After recognising damages to the site, the Gruppo di Ricerche Preistoriche ed Archeologiche “Alberto Carlo Blanc” dug the remaining deposit to recover and salvage the archaeological evidence. These excavations took part in the northern sector of the site on the 18th of June 1972, 25th of June 1972, 23rd of July 1972, 27th of August 1972 and 8th of April 1973 ( Gennai, 2023).

**Figure 6.**
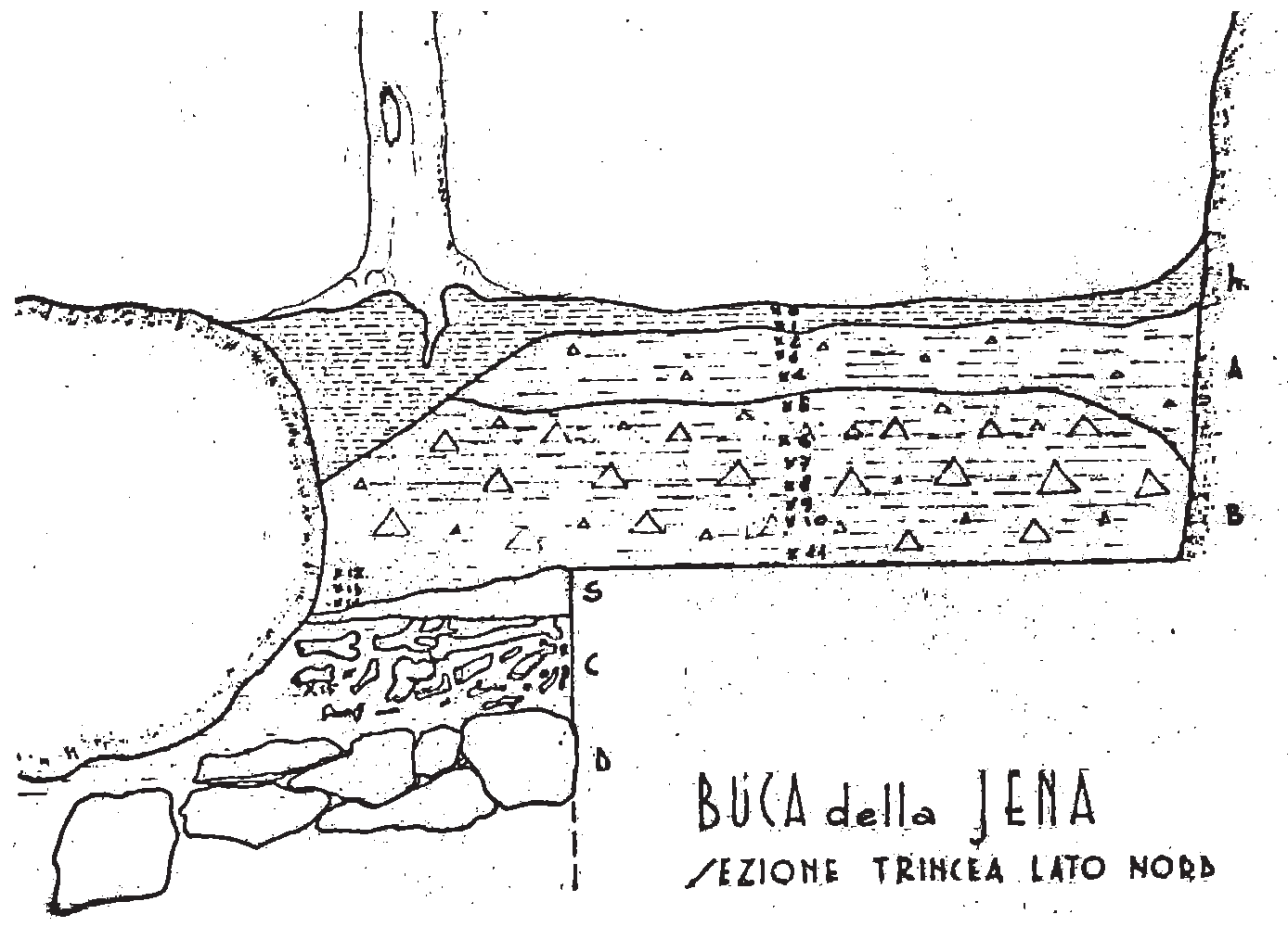
Buca della Iena 1966 northern profile section original drawing. Courtesy of Gino Fornaciari.

According to the original field notes ( Gennai, 2023), the systematic excavation proceeded opening subsequent trenches on the terrace. While the position of these trenches (named sectors in the field notes) can be deduced from field notes, their extent is not recorded. The deposit sequence was explored through arbitrary spits, and sedimentological similarities were used to correlate them to each other. A total of fifteen spits are mentioned, starting from the number 0. Each spit measured roughly 10 to 20 cm, except the last one (no. 15) measuring 50 cm ( Figure 6; Gennai, 2023). The excavated deposit reached 2,75 m depth from the floor level ( Fornaciari, 1966).

The first investigated sectors were A and B. The field notes mention four spits for sector A, two spits for sector B, and a total one-metre depth for both sectors. Artefacts attributed to the Mousterian were found in the two upper spits, associated with Pleistocene fauna like cave bear (Ursus spelaeus), while the lower spits only yielded Pleistocene fauna.

Sector C and Sector D followed. Sector D was recorded between Sector C on the left and Sector A on the right. There are thirteen recorded spits in D, while C consists of twelve spits. The two sectors are divided by an olive tree, whose roots and agricultural works highly disturbed the sediments until spit 3. In spit 2 a diagnostic Gravettian or Epigravettian (Upper Palaeolithic) backed point and additional volumetric small blades were found. The following spits showed a progressive increase of limestone fragments and hardening of the sediments, culminating in the appearance of flowstone starting in spits 11 and 12, depending on the sector. The artefacts were all attributed to the Mousterian, and they were mixed with Pleistocene fauna like hyena, cave bear, horse, and rhino.

**Figure 7.**
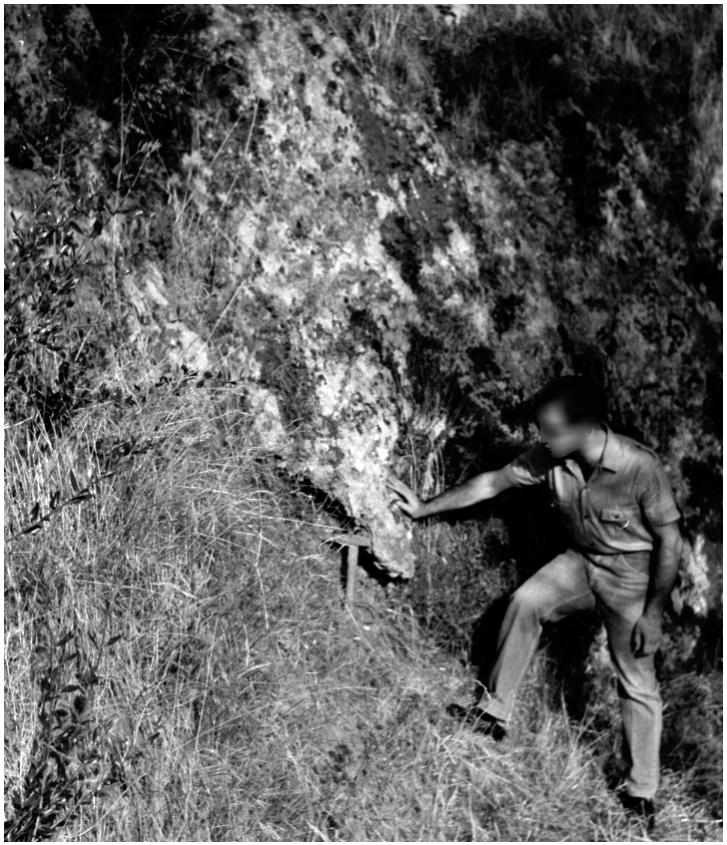
The Buca della Iena before excavation in 1966. Photo courtesy of Gino Fornaciari.

Sector E corresponded to the area of the niche in the limestone wall, which was completely covered before excavation ( Figure 7; Gennai, 2023). It consisted of seven spits, the first one being correlated with spit 5 of sectors C and D. As sediments probably sloped and compacted inside, the sedimentological sequence has less resolution. Hence, spit X corresponds to spits 8, 9, and 10 in sectors C and D. As in the other sectors, sediments transitioned from loose clay to a more cemented one. Deeper and more inside the niche, the deposit shifted to a clast-supported sediment with middle-sized stones. Also, sector E yielded Mousterian artefacts mixed with Pleistocene fauna, mostly cave bear and hyaena.

**Figure 8.**
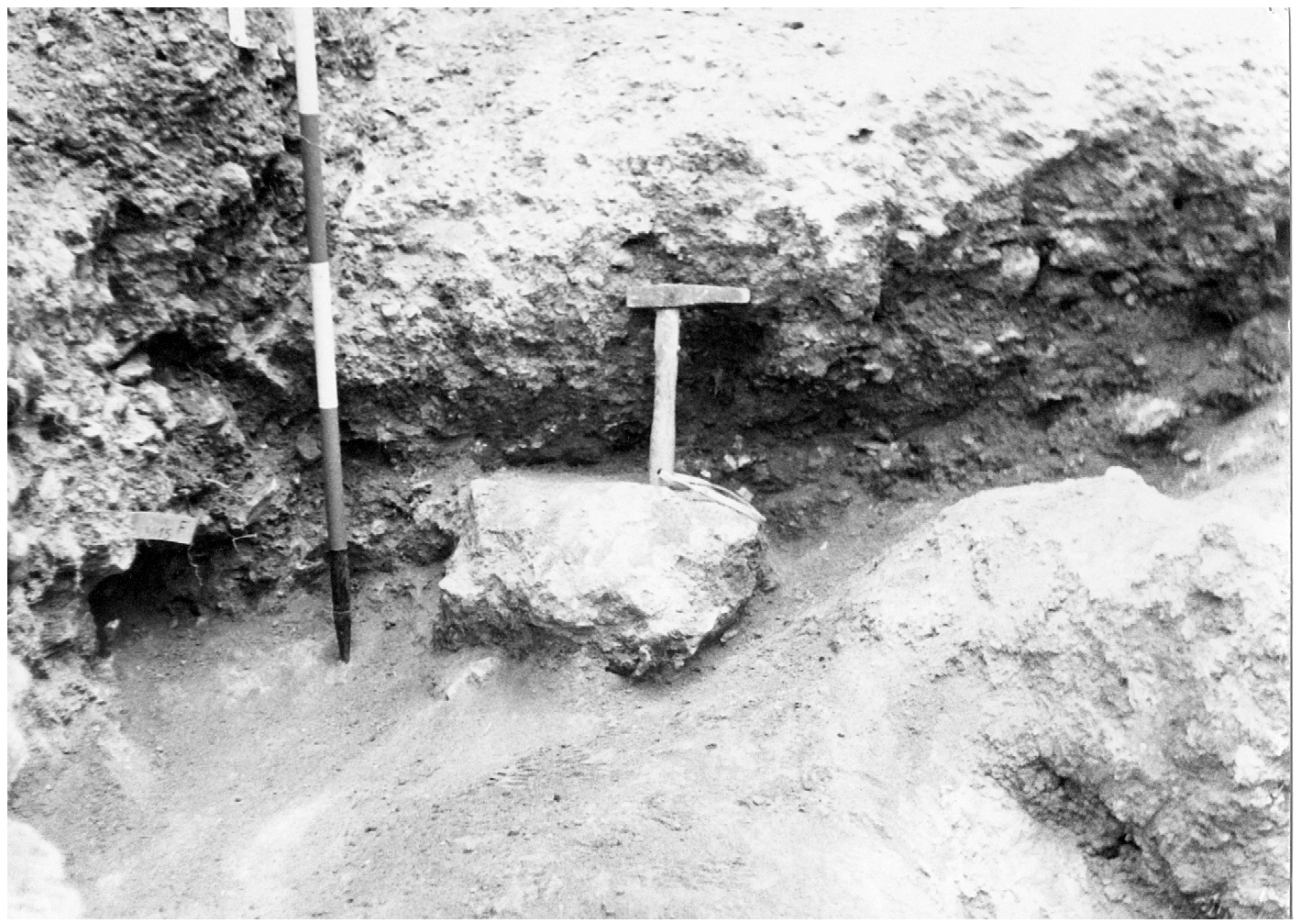
The bottom of the Buca della Iena sequence after excavation in 1966, flowstone closeup. Photo courtesy of Gino Fornaciari.

Finally, sector F was dug. The sector was heavily impacted by an olive tree and by the earliest test-pits meaning that only a small portion of deposit was available for excavation. Sector F consists of eight spits, starting with spit 7 and ending with spit 15, the lowest in the whole excavation. Therefore, sector F is likely located on the slope of the terrace and partially limited by a big limestone boulder, nowadays at the western edge of the terrace. The deposit is similar to that uncovered in the other sectors, increasing in clastic content toward the bottom and hardening progressively. The flowstone is reached in spit 14. Underneath the flowstone, whose lower end is encasing fauna bones, the sediment changes in light-coloured clay with abundant Pleistocene fauna, mostly hyaena and horse, and devoid of any artefacts ( Figure 8; Gennai, 2023). Underneath the clay, there were big blocks interpreted as the bedrock.

**Figure 9.**
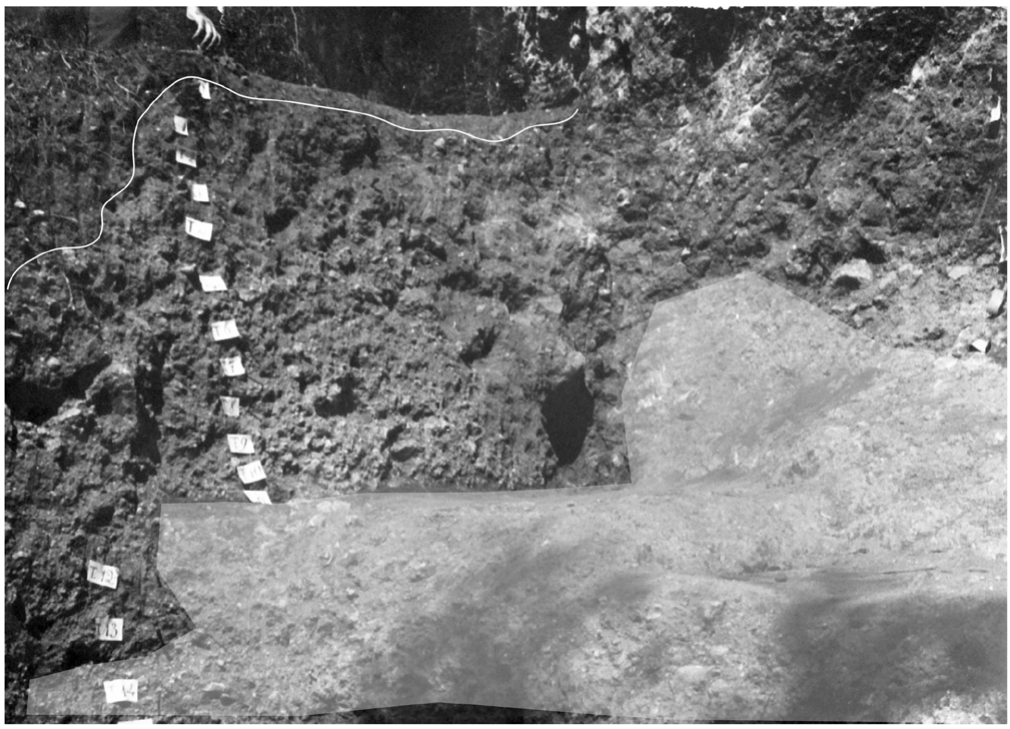
The Buca della Iena northern profile section after excavation in 1966. White line: disturbed sediment, White area: flowstone. Photo courtesy Gino Fornaciari, modified by Jacopo Gennai.

Therefore, the deposit is interpreted as having an upper humic level covering silty reddish-brown sediments with a higher content of limestone and progressive hardening towards the bottom. The flowstone is a clear break in sedimentation because underneath the sedimenti is changing in colour, yellowish, and texture, loamy ( Fornaciari, 1966). The subsequent analysis of the sequence by the University of Pisa confirmed the excavators’ impressions ( Pitti & Tozzi, 1971). Both publications ( Fornaciari, 1966; Pitti & Tozzi, 1971) reported the thickness and the sequence of lithological layers, which are difficult to ascertain against the fieldwork notes ( Figure 9; Gennai, 2023).

- Humic level (h)
- Silty sandy brown-reddish loose sediment with gravel, 70 – 50 cm (Layer A)
- Greyish silty sandy sediment with gravel, increasing towards the bottom and progressively more compact 80 – 60 cm (Layer B). Pitti and Tozzi (1971) refined the knowledge of the main deposit (layer B), operating subdivisions according to the gravel content and the progressing hardening of the sediment.
- Fornaciari (1966) recorded a 25 cm thick brown loamy with a gravel layer, located in between the flowstone and the main deposit, which is not accounted for in Pitti and Tozzi (1971)
- Flowstone, 35 – 10 cm
- Yellowish loamy sediment, 50 – 30 cm
- Boulders

In 1968, samples of the flowstone were radiometrically dated with the Th230/U230 and gave two dates (< 41000 and < 51000 years), the discrepancy is due to the high content of clay in the flowstone ( Fornaca-Rinaldi & Radmilli, 1968). Because of the clay contamination and similarity with the nearby date of Grotta all’Onda, also obtained on a flowstone underneath sediment with Middle Palaeolithic artefacts, the younger date is favoured ( Pitti & Tozzi, 1971).

In the following years, the archaeological group lamented several damages to the remaining deposit, mainly due to looters. Therefore, to salvage the remaining deposits and archaeological evidence they excavated the northern profile in 1972 and 1973 ( Gennai, 2023). The new sectors were named A and B, progressing towards the North. In sector A the deposit was dug in 5 spits, all 30 cm thick except the initial spit 0, 23 cm thick, corresponding to the humic level. The deposit in A sloped towards the Buca della Iena niche (to the south) and consisted of mostly yellowish clay, becoming more reddish towards the bottom and with increasing stone content. A big boulder spanning the whole sequence was finally extracted in spit 4. In spit 4 a piece of flowstone was found too. Therefore, the excavators are believed to have reached the end of the stratigraphical sequence. In sector B, four spits, each 30 cm thick, were dug. The sediment was much looser and consisted of reddish clay with loose gravel. The findings were similar to the previous excavations: Pleistocene fauna and some, dubious, Middle Palaeolithic artefacts.

### Grotta del Cervo

The site is adjacent to Buca della Iena, opening on the northern-facing side of the hill (43.9143446 N, 10.2850449 E; Figure 2; Gennai, 2023). The site is a bigger cavity than Buca della Iena, featuring two branches. It has never received an official name, being called like the nearby main village: Piano di Mommio. Informally, it is known as Grotta del Cervo ( Galiberti, 1997). The cave mouth faces West, the excavation was limited to the West by a drystone wall forming the modern terrace edge, to the South and to the South-West by the Buca della Iena northern profile and a big limestone boulder, as to the North additional boulders were present.

**Figure 10.**
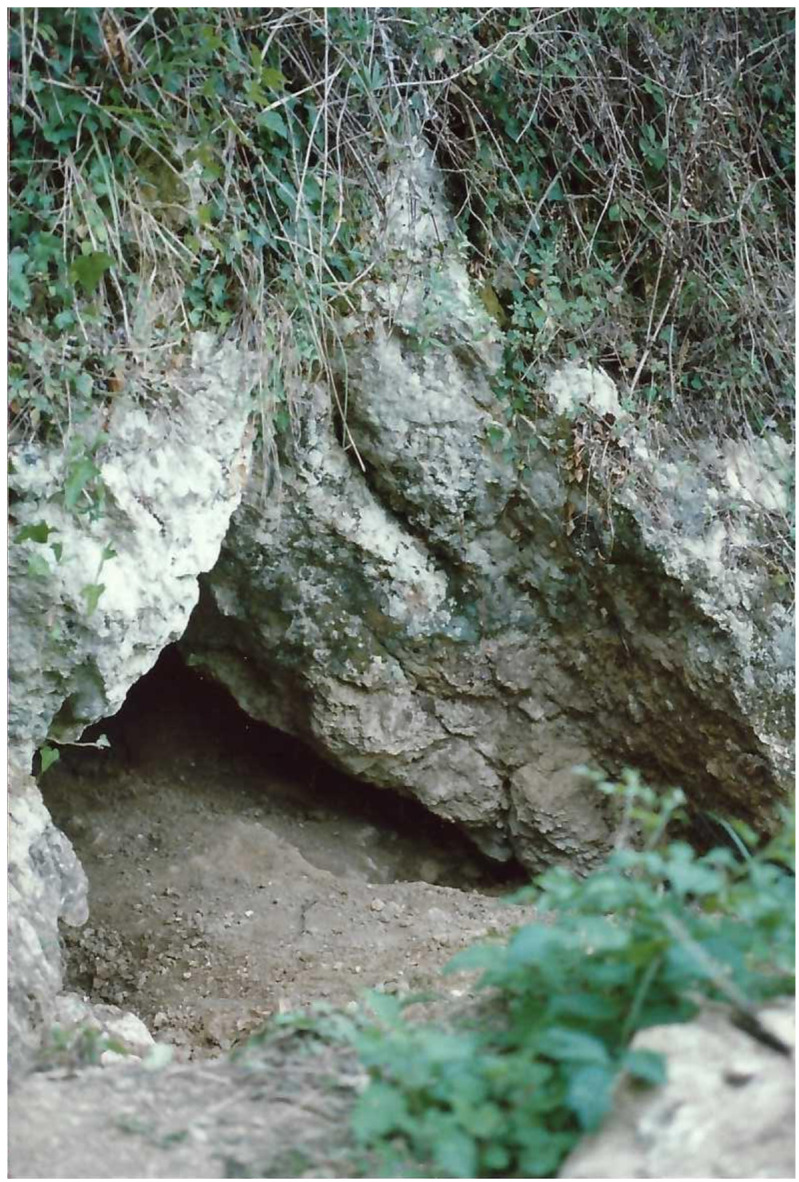
The Grotta del Cervo opening before excavation in 1988. Photo courtesy of Museo Civico and Archeologico e dell’Uomo “Carlo Alberto Blanc”.

The opening might have been discovered during the Buca della Iena excavations in 1972-1973, but systematic excavations started in 1989 ( Cocchi-Genick, 1989; Gennai, 2023). Before excavation the opening was 3.85 m wide, 2,5 m deep and 0.80 m high ( Figure 10; Gennai, 2023). The excavation took place in four different campaigns in 1989 - 1992 and excavated the whole site, reaching 3.30 m of depth ( Cocchi-Genick, 1993; Cocchi-Genick, 1992; Cocchi-Genick, 1989). The site was divided into a 1 m ^2^ grid, with numbers from 1 to 7 progressing South to North on the X axis and letters from A to F progressing East to West on the Y axis.

This way, the deposit is roughly divided into two areas: the external deposit, corresponding to squares D1-F7, and the inner deposit, corresponding to squares C1-A7. The excavation proceeded by 5 cm spits. The soil was wet-sieved with a 2 mm mesh.

**Figure 11.**
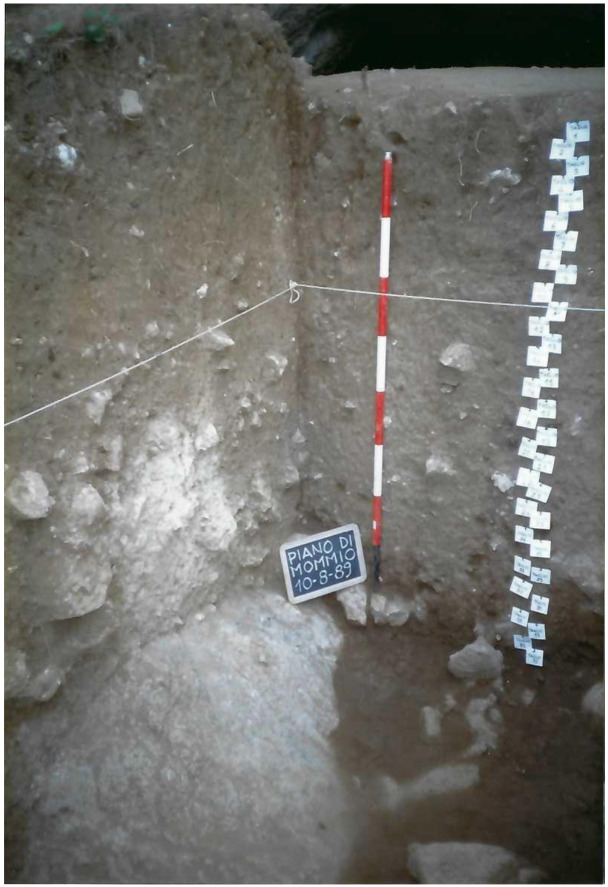
Grotta del Cervo complete excavated sequence, external area. Photo courtesy of Museo Civico and Archeologico e dell’Uomo “Carlo Alberto Blanc”.

During the first campaign, a 2x2 m trench was opened outside (F4-F5-E4-E5) and a second trench of 1x7 m opened a whole transect (D1-D7). The excavation of the external trench removed the humic level up to 30 cm depth and then reddish silt mixed with gravel up to one metre depth from point 0. The same depth was reached in the inner cave and then excavation continued in the two branches, yielding human remains in B1 (-0.80 m and -1.80 m), A6 (-0.80 m) and B6 (-1.32 m). In C2 also a fragment of pottery was found (-1.30 m). The human remains, belonging to various juvenile and adult individuals, and the pottery fragment revealed a probable secondary burial that happened during the Copper Age, as attested by other nearby sites ( Fornaciari, 1977). The excavation stopped at 1.30 m depth in the northern branch arriving on top of a big stones layer and 2.20 m depth in the southern branch. Instead, in the external trench, it reached 2.90 m depth. The sediment is a homogenous reddish silt with gravel and bigger stones in the lower spits ( Figure 11; Gennai, 2023). Pleistocene fauna started appearing at 1,05 m depth with higher frequency in the lower spits, Mousterian artefacts started appearing in spit 13 (1.65 – 1.70 m depth), with a higher frequency in the lower spits. The sediments appeared deeply reworked by fossorial animals and agricultural works, only at -2.80 m (spit 36) depth did the sediments hardened and were preserved.

**Figure 12.**
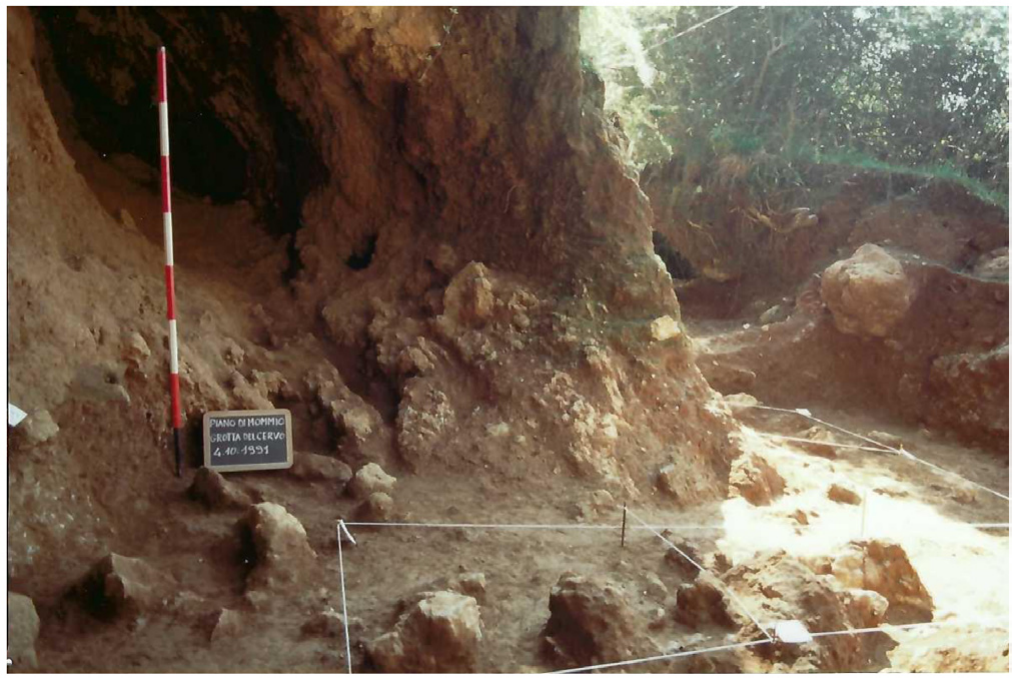
Grotta del Cervo southern branch, bottom of the sequence. The olive tree marks the border with the Buca della Iena excavation. Photo courtesy of Museo Civico and Archeologico e dell’Uomo “Carlo Alberto Blanc”.

During the second campaign, the southern branch of the cave was totally excavated until the stony level (2.40 m depth; Figure 12; Gennai, 2023). The rest of the external trench was enlarged, finding a similar deposit and with Pleistocene findings mostly coming from 2.40 m depth onwards.

The third campaign reached the 2.90 m depth, previously reached outside, inside the cave and expanded the external trench. Only sparse Mousterian lithic artefacts and Pleistocene fauna were found in the lower spits.

The fourth campaign reached the 3.30 m depth inside and externally, the sediment showing hardening and a higher frequency of Pleistocene fauna and some Mousterian lithic artefacts.

### Grotta del Capriolo

**Figure 13.**
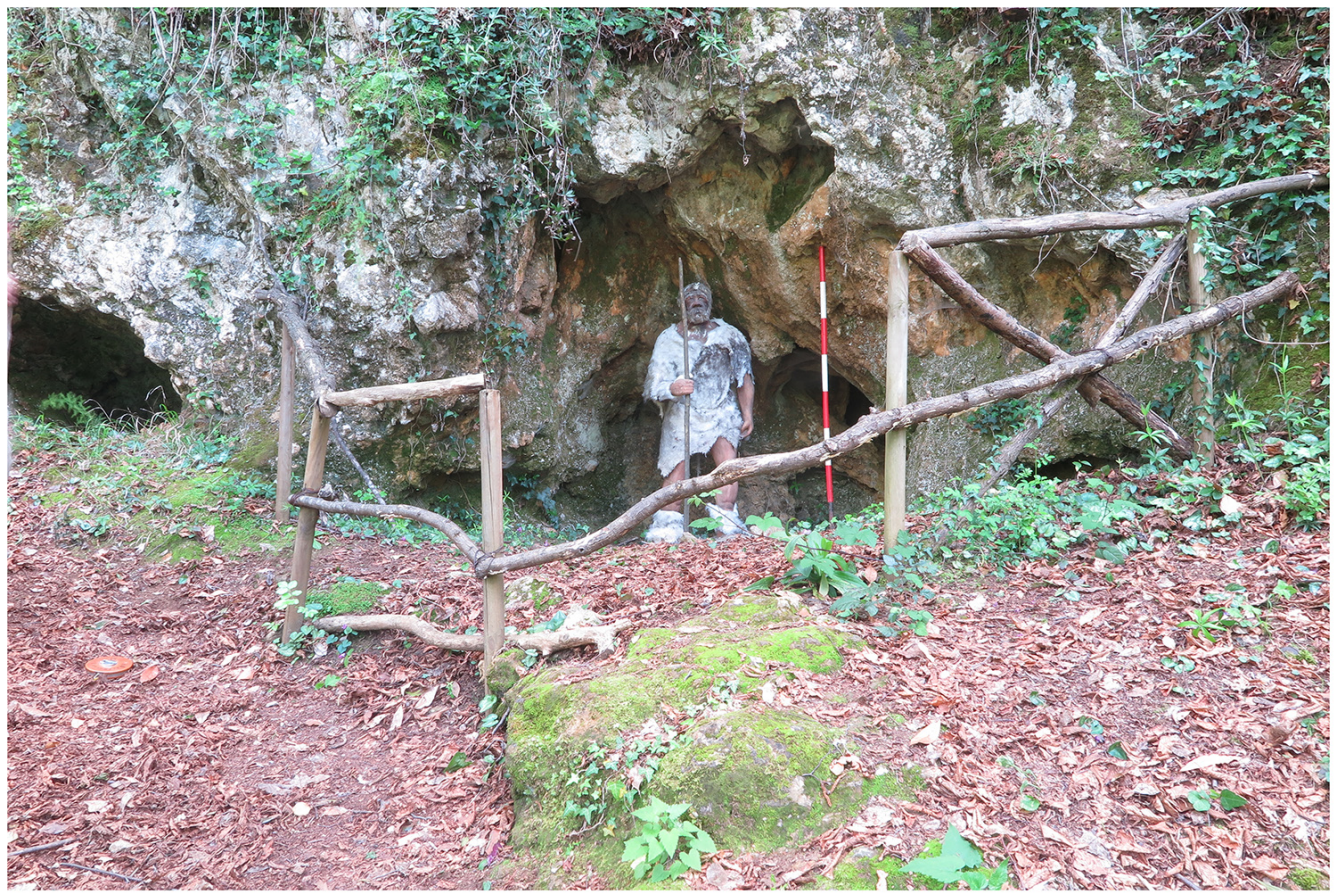
Grotta del Capriolo modern view. Photo Jacopo Gennai.

The Grotta del Capriolo is facing the Grotta del Cervo and Buca della Iena from the southern slope of the canyon at around 90 m a.s.l. (43.9136514 N, 10.2847033 E – Figure 2; Gennai, 2023). The site is a small cave opening in the same limestone outcrop as the above-mentioned sites, running continuously in the whole area. The opening faces to the North and the whole area is comprised of 20 m ^2^. The cave measures approximately 4.00 m in width, 3.00 m in length and 2.50 m in height ( Figure 13; Gennai, 2023).

**Figure 14.**
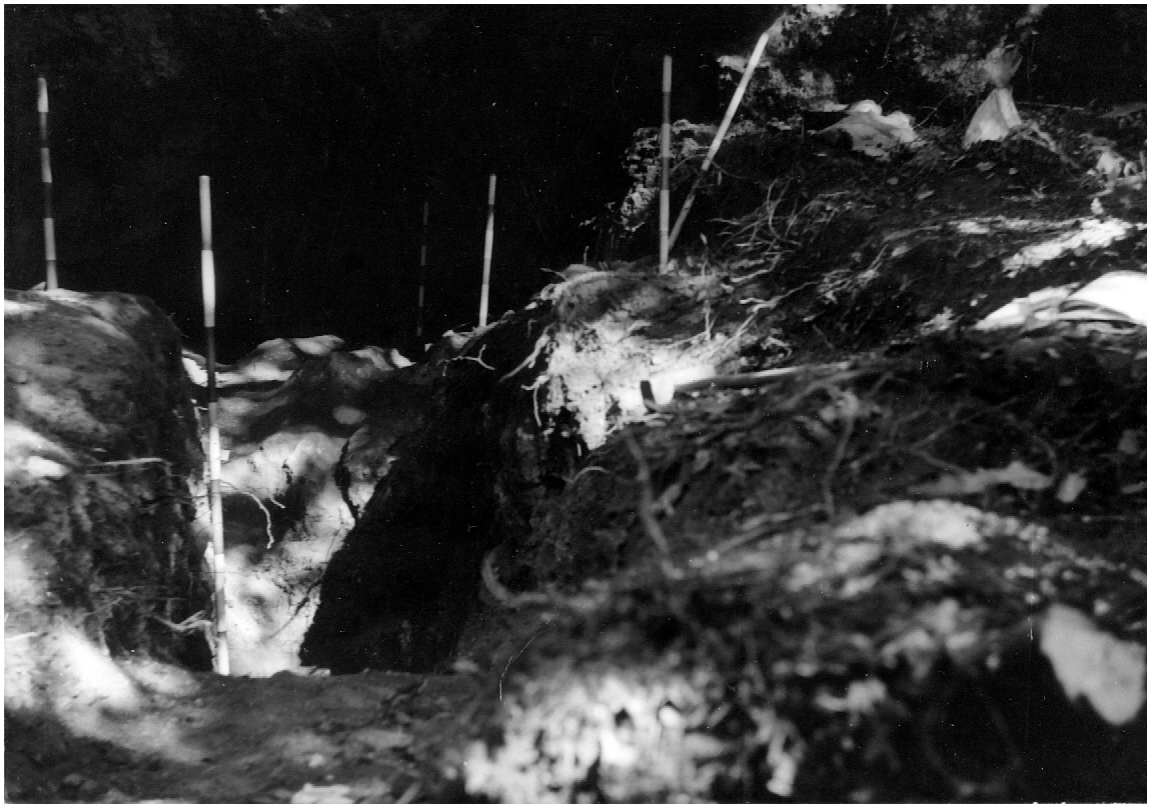
Grotta del Capriolo during excavation in 1968. Photo courtesy of Gino Fornaciari.

The site was discovered in February 1968 and found completely covered by the slope deposit ( Figure 14; Gennai, 2023). The site was excavated between the 24th of July and the 7th of August 1968 by the “Alberto Carlo Blanc” archaeological Group ( Radmilli, 1968). Later in May-June 1970, the University of Pisa excavated the remaining deposit ( Pitti & Tozzi, 1971).

**Figure 15.**
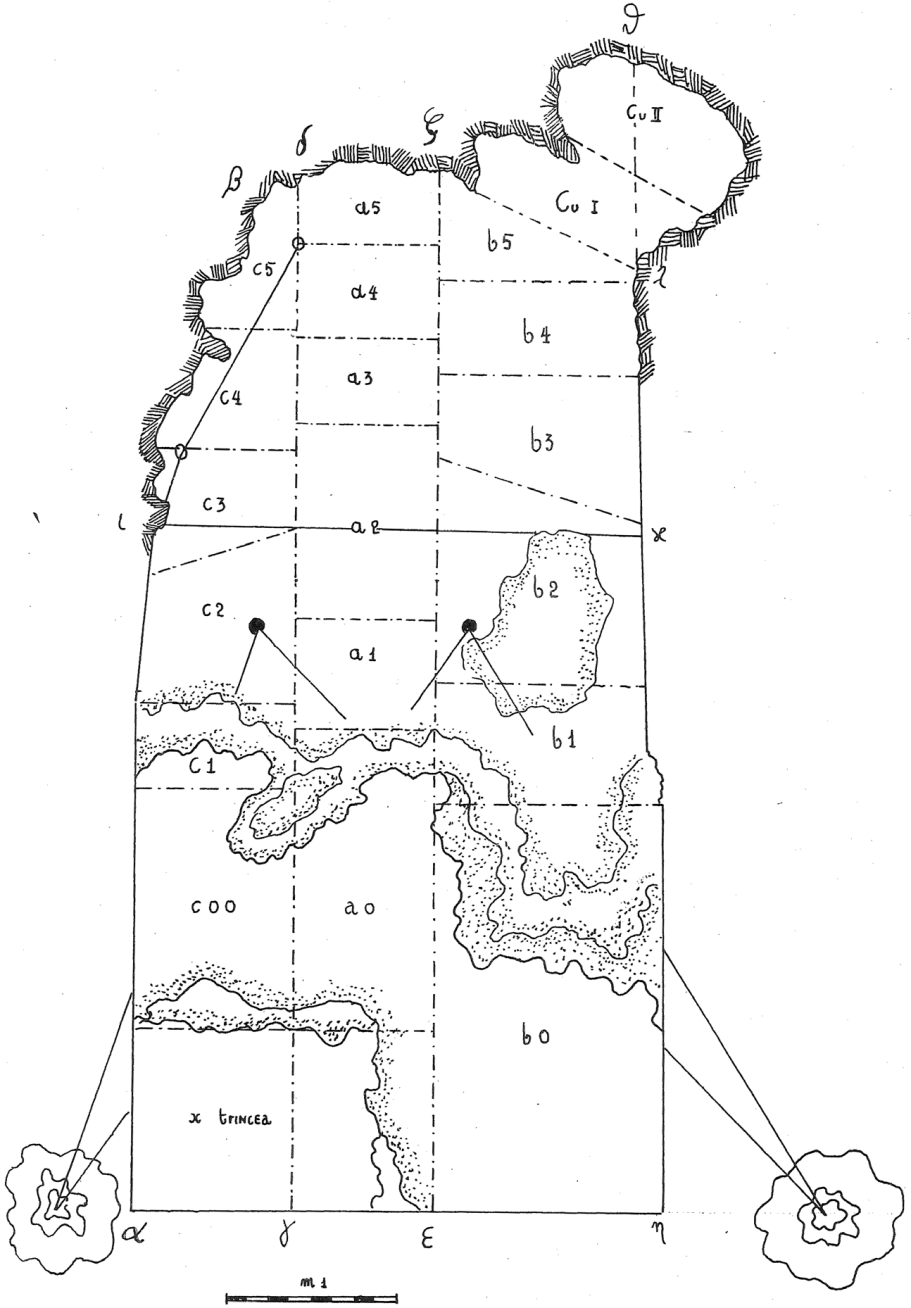
Grotta del Capriolo original site excavation plan 1968. Courtesy of Gino Fornaciari.

In 1968, the excavation of the site involved the creation of three trenches, each extending from the outermost to the innermost areas of the cave. Initially, they dug trench A, positioned at the central point, followed by trench B in the western sector, and finally trench C along the eastern cave wall. Trenches are divided into sectors, numbered sequentially from 0 to 5 as they progress from the exterior to the interior. Trenches and sectors do not follow a specific grid and they differ in extent ( Figure 15; Table 2; Gennai, 2023) Sector 0, yielded little archaeological sediment. Towards the back of the cave, two sectors, Cu I and Cu II, are covering a small niche.

**Figure 16.**
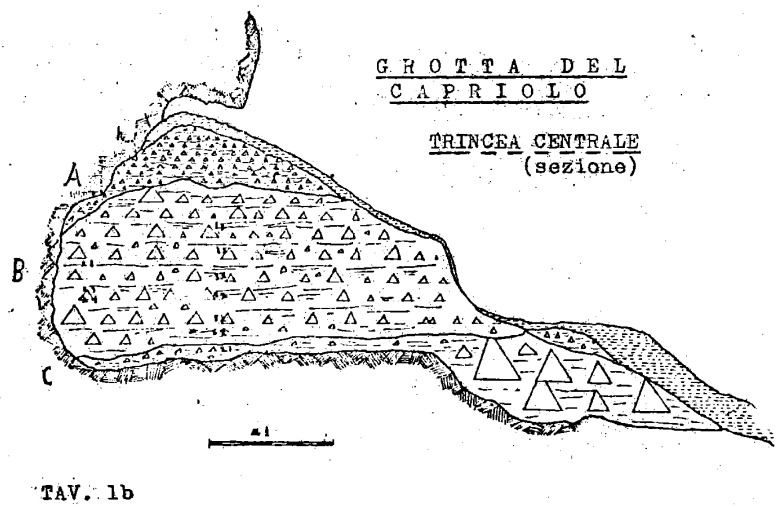
Grotta del Capriolo original Trench A profile section drawing.

**Figure 17.**
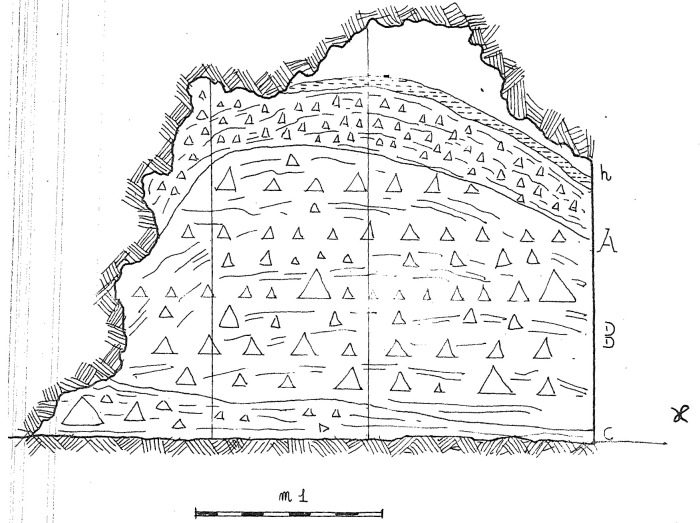
Grotta del Capriolo original transversal (C3-A3-B2/B3) profile section drawing. Courtesy of Gino Fornaciari.

**Figure 18.**
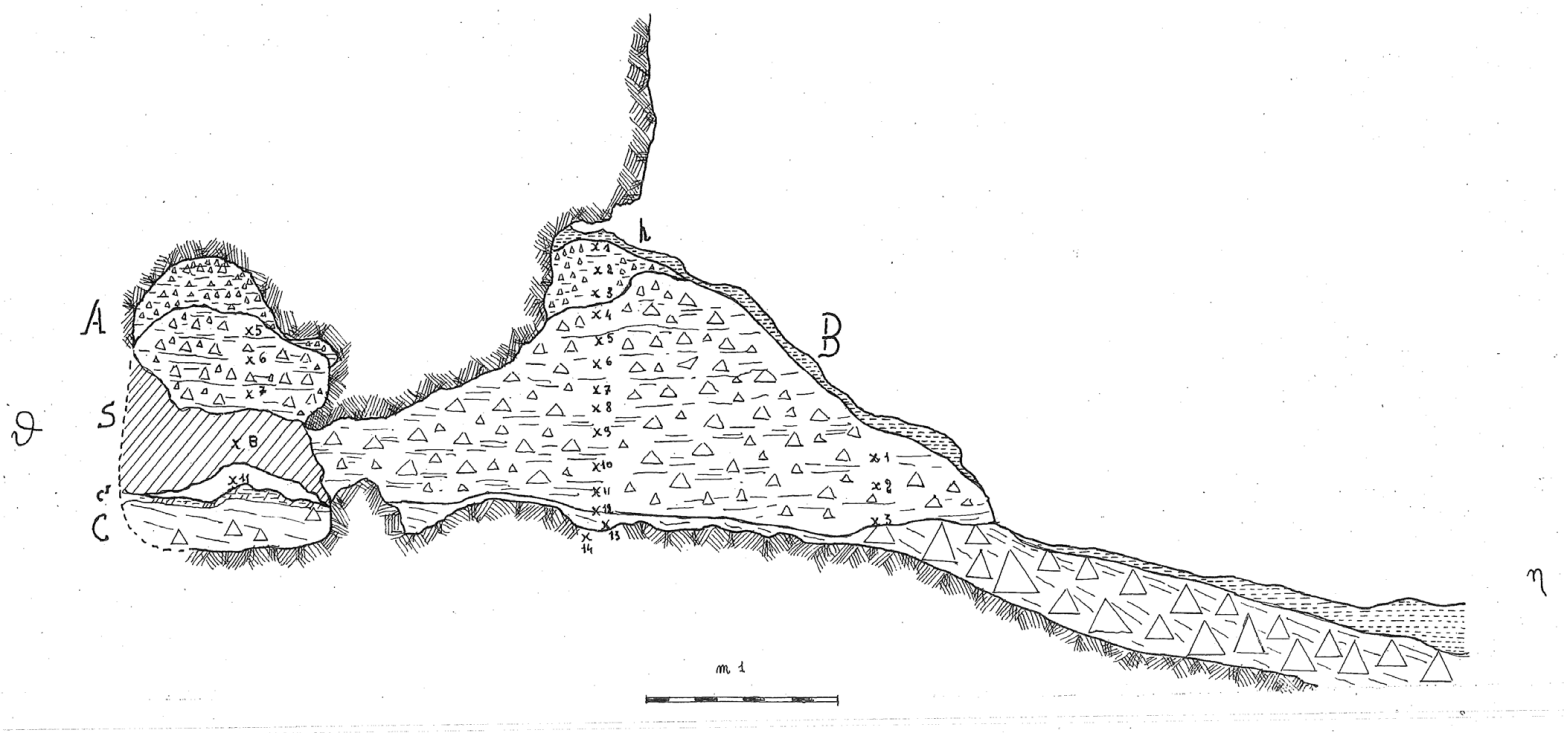
Grotta del Capriolo original Trench B profile section drawing. Courtesy of Gino Fornaciari.

The deposit was excavated in spits, with the total number of spits varying based on factors such as the specific sector, deposit thickness, and the inherent thickness of the spits themselves. For instance, trench A, at its highest point, comprised 7 spits ( Figure 16; Figure 17; Gennai, 2023), while trench B featured 14 spits ( Figure 18; Gennai, 2023), and trench C had 11 spits. The thickness of these spits fluctuated, ranging from 30-40 cm down to 10 cm. Dry sieving was conducted on-site, enabling the recovery of artefacts as small as 10 mm in their maximum dimension.

In 1970, excavation focused on the remaining sediment, which was designated as trench DE ( Gennai, 2023). Unfortunately, only a few fieldwork notes have survived from this period. The deposit was excavated in arbitrary spit, beginning with spit A9 and continuing up to A15. Other spits were denoted with the letters D, E1, E2, and F. The starting excavation height was 50 cm from a reference point, although the exact reference point height remains undocumented. Nevertheless, spatial coordinates (x, y, z) are recorded for some of the artefacts.

The excavations reported a similar stratigraphy.

- Superficial humic level, 5 – 20 cm (h)
- brown-reddish silt with little gravel and devoid of any archaeological material, 20 - 50 cm (A)
- light brown silt with progressively increasing gravel bearing archaeological Mousterian artefacts and Pleistocene fauna, 60/95 – 130 cm (B)
- yellow silt covering clast-supported sediment and big boulders interpreted as the bottom of the sequence, 5 – 20 cm (C or D)

The 1968 excavations also recognised a flowstone in Cu I and Cu II. A much more cemented area of sediment is found in the back of the cave (S). The 1970 excavation subdivided layer B into three horizons following the progressive increase of gravel and hardening of the sediments ( Pitti & Tozzi, 1971).

## Discussion: new stratigraphical interpretation

### Buca della Iena

The absence of quotes taken from a conventional point zero and the reporting of average thicknesses allow only a rough estimate of the extent and position of the lithological layers. Also, the position of the spits on the profile section cannot be translated into precise measurements. Therefore, only a reconstruction of the spits’ relative positions within the stratigraphy and their correlations, reported in the fieldwork notes, is here proposed.

**Figure 19.**
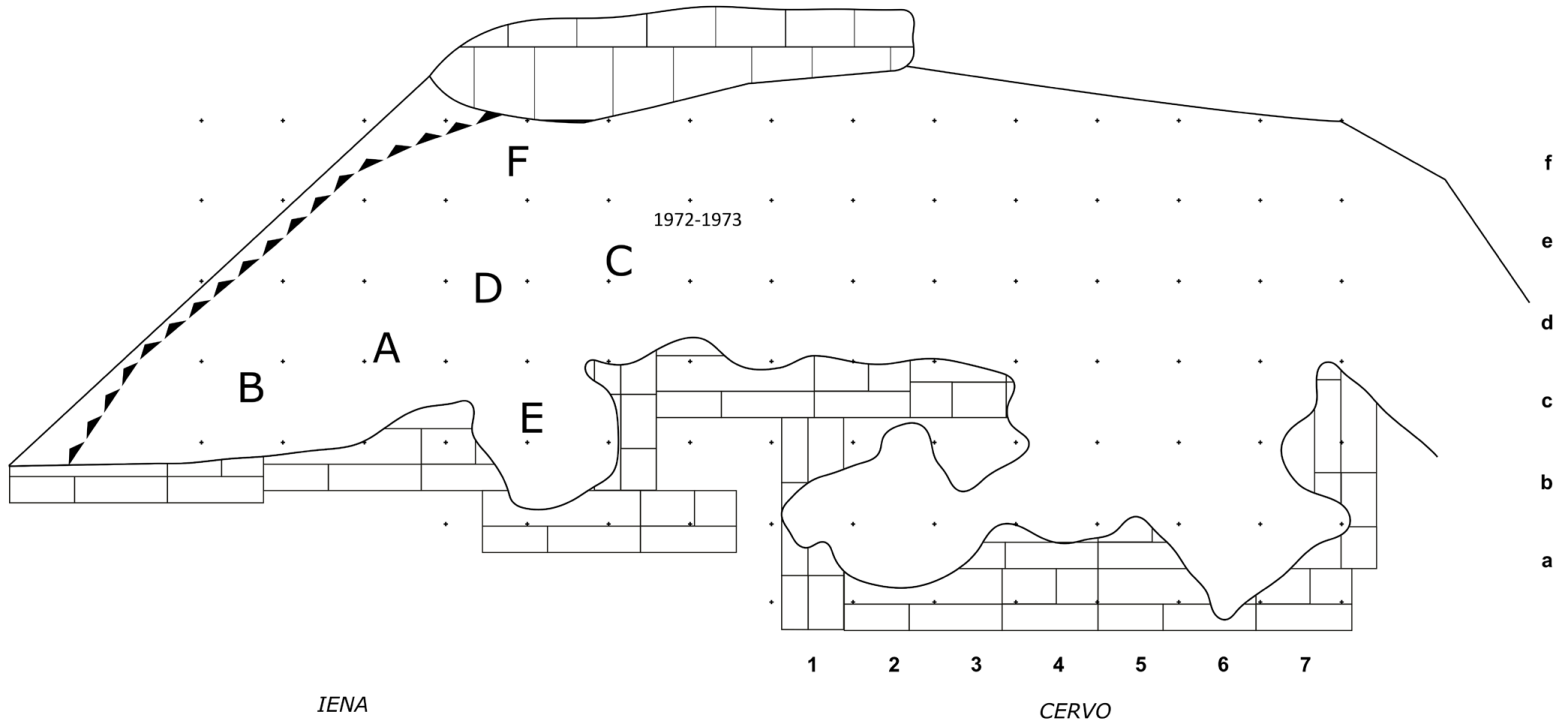
Buca della Iena and Grotta del Cervo redrawn site plan. Drawing Jacopo Gennai.

**Figure 20.**
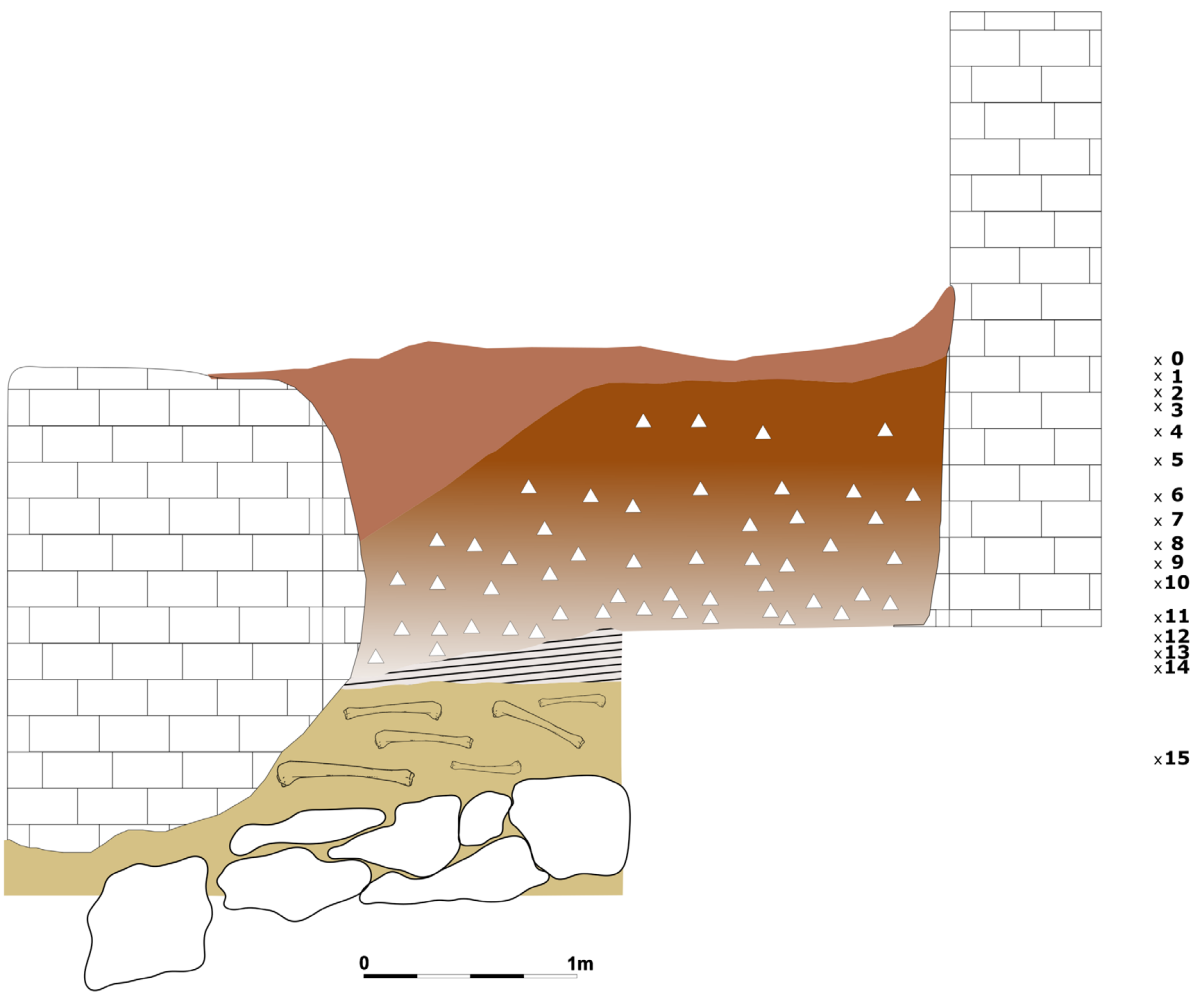
Buca della Iena northern profile section redrawn. Drawing Jacopo Gennai.

Nowadays the current extent of the terrace accounts for a roughly 25 m ^2^ area ( Figure 19; Gennai, 2023). Reconstruction of excavation sectors goes as follows: to the north sectors A and B (1972–73) and sector C run across the width of the terrace from the limestone wall to the big boulder. More to the south, in front of the opening of the niche, supposedly lays sector D and in front sector F, representing the edge of the terrace with sediments and the flowstone sloping downwards. Sectors C and D were the most intact and the highest of the whole deposit ( Figure 20; Gennai, 2023). Their sediments sloped frontally towards F, which showed a shift of the same sediments one spit lower than D and C. Sector C and D sediments covered the opening of the niche which corresponded to the sector E. Sector E sediments probably sloped inside and compacted, also the weathering of the wall accumulated more clastic sediments towards the cave end. To the South, sector A was probably sloping laterally and frontally because the deposit was only one metre high and excavated in four spits. B was probably in the front, on the slope, because only two spits are mentioned and sedimentologically the B – 2 is correlated with F – 14/15. The yellow clay is found only in these two lowermost spits ( Table 1; Gennai, 2023).

**Table 1.** Buca della Iena. Correlation of spits in the different sectors.

B	A	C	D	E	F
		1	1		
		2	2		
		3	3		
		4	4		
		5	5	5	
		6	6	6	
	1	7	7	7	7
		8	8	X	8
	2	9	9		9
	3	10	10		10
	4	11	11	11	11
		12	12	12	12
1				13	13
			13	14	
2					
					15

**Table 2.** Grotta del Capriolo area of each sector and total area of excavation.

Sectors	B1	B2	B3	B4	B5	Cu I	Cu II	A1	A2	A3	A4	A5	C1	C2	C3	C4	C5	Tozzi trench	Tot
Area (m2)	0,92	1,64	1,07	0,63	0,78	0,57	0,51	0,53	0,94	0,41	0,45	0,36	0,47	0,91	0,38	0,48	0,33	1,7	13,07

### Grotta del Cervo

**Figure 21.**
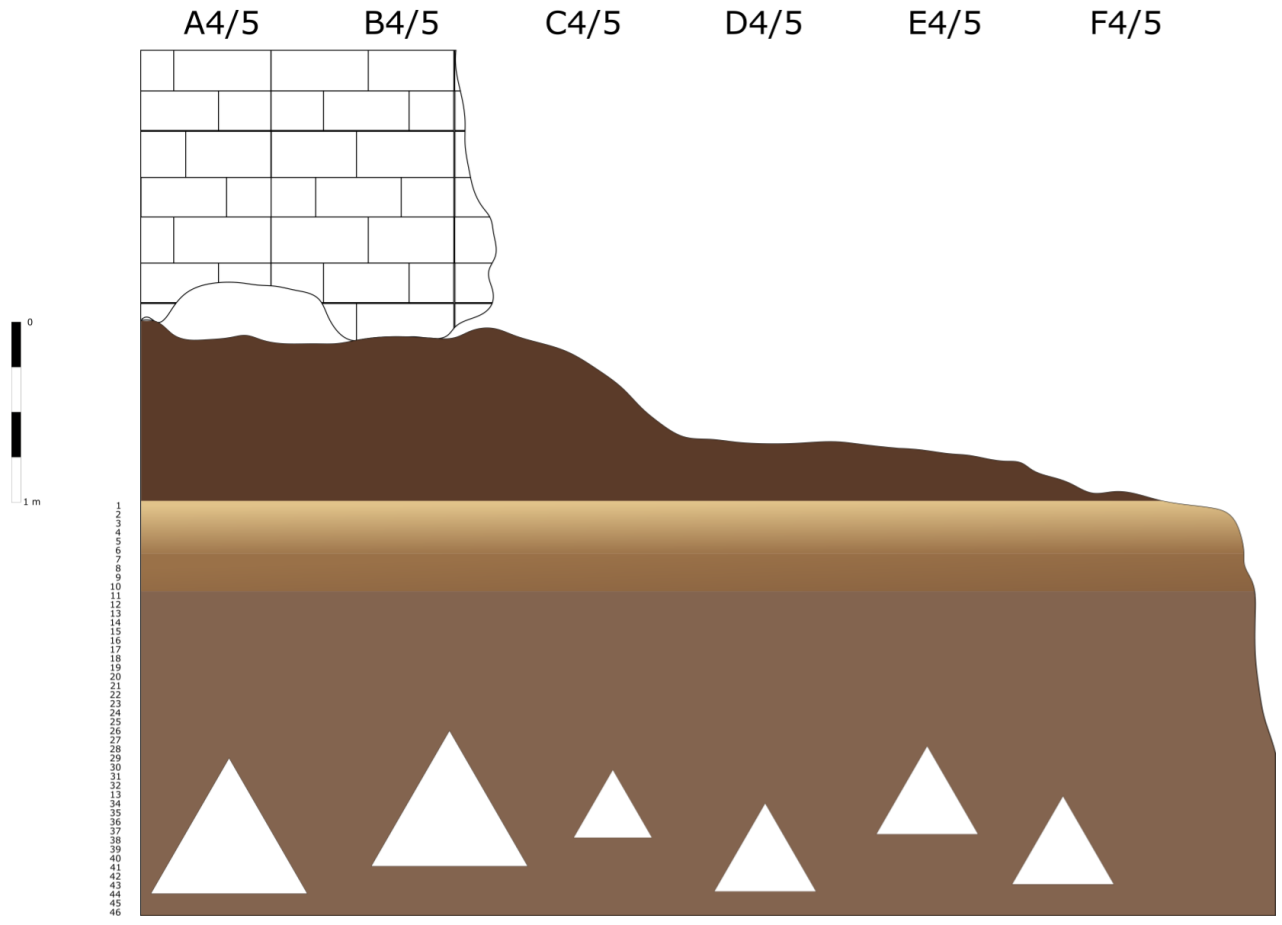
Grotta del Cervo profile section redrawn. Drawing Jacopo Gennai.

Grotta del Cervo shows many analogies with the adjacent Buca della Iena. The sediment is a brownish-reddish loose silt with gravel progressively hardening with depth ( Figure 21; Gennai, 2023). Also, the increase of stones and boulders in the lower spits is similar to the stratigraphical sequence in Buca della Iena. Tentative correlations might be operated between spit 5 - 6 of Buca della Iena and spit 1 of Grotta del Cervo (1.05 – 1.10 m depth). Buca della Iena and the adjacent Grotta del Cervo might have been part of the same site frequented by Middle Palaeolithic groups.

### Grotta del Capriolo

**Figure 22.**
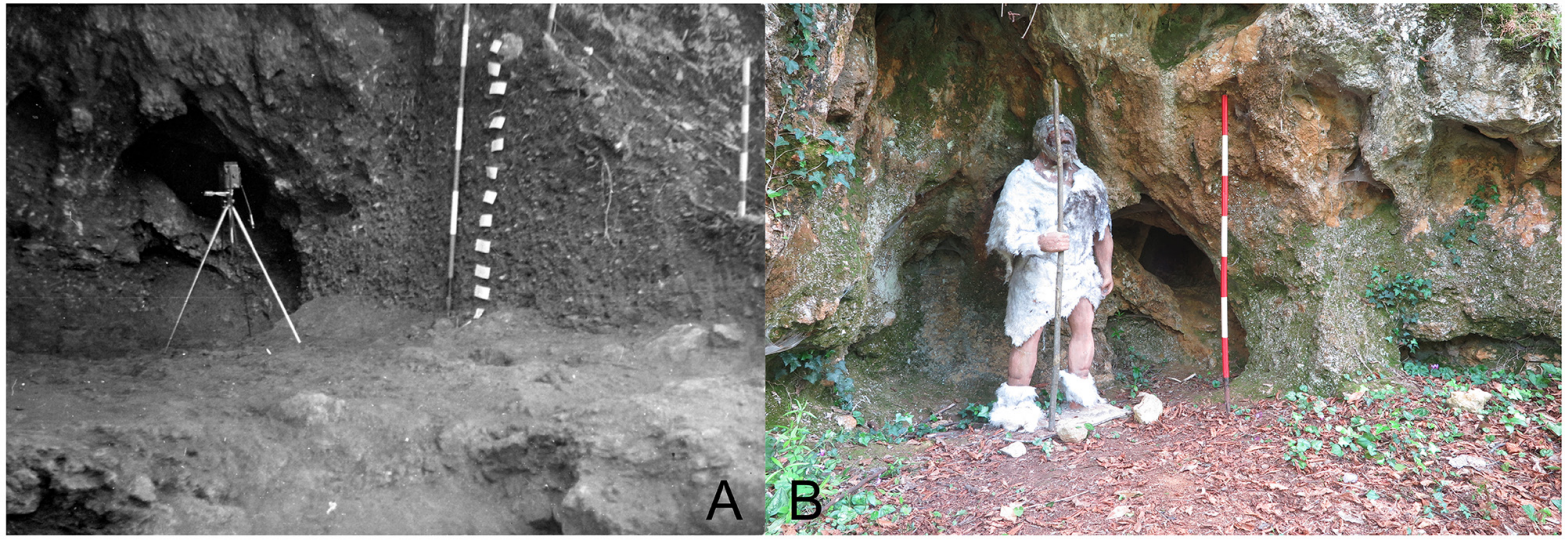
Comparison between the end of 1968 Grotta del Capriolo excavation ( **a**) and modern state ( **b**). Photo Jacopo Gennai and Gino Fornaciari.

**Figure 23.**
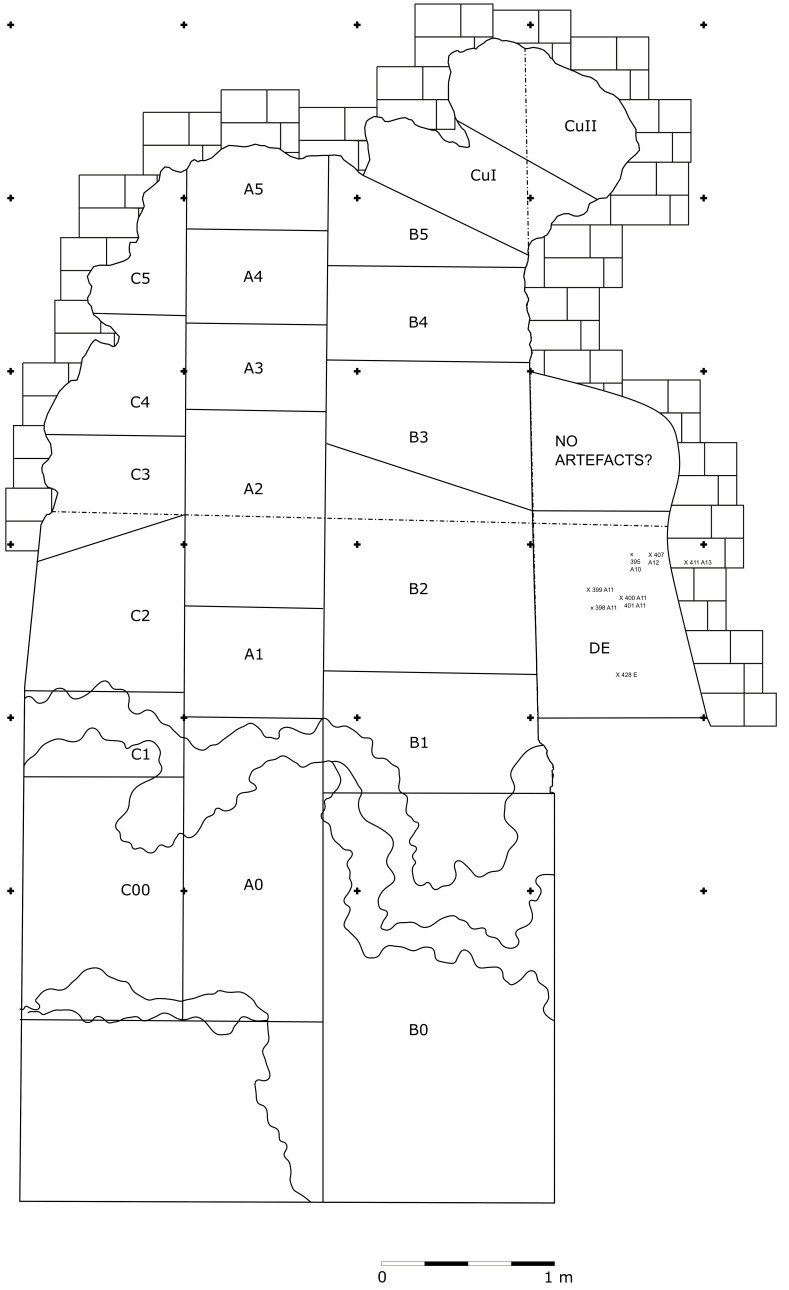
Grotta del Capriolo redrawn site plan including trench DE. Drawing Jacopo Gennai.

**Figure 24.**
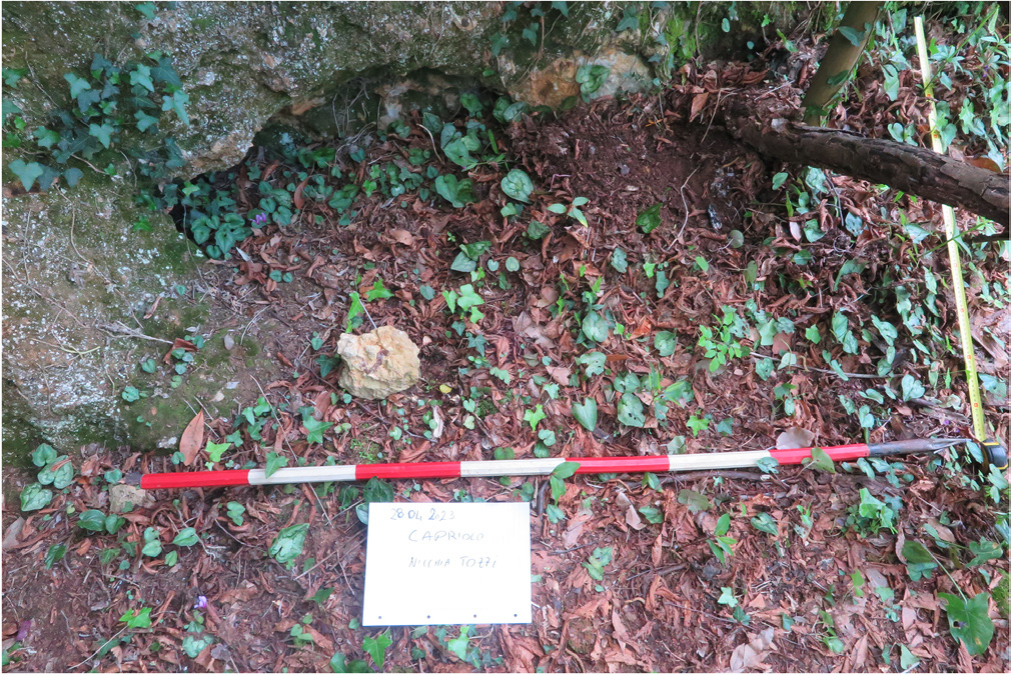
The likely location of trench DE. Photo Jacopo Gennai.

**Figure 25.**
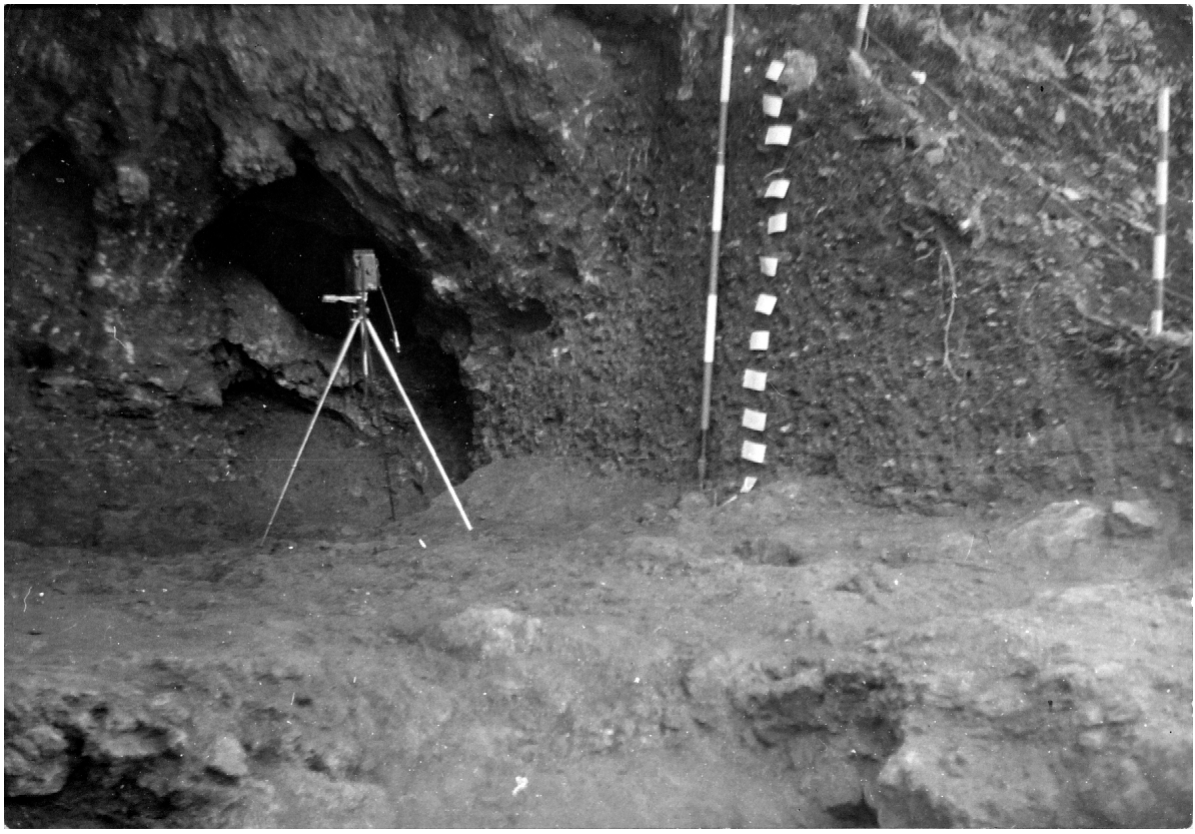
The end of 1968 Grotta del Capriolo excavation and Trench B profile section. Photo Gino Fornaciari.

**Figure 26.**
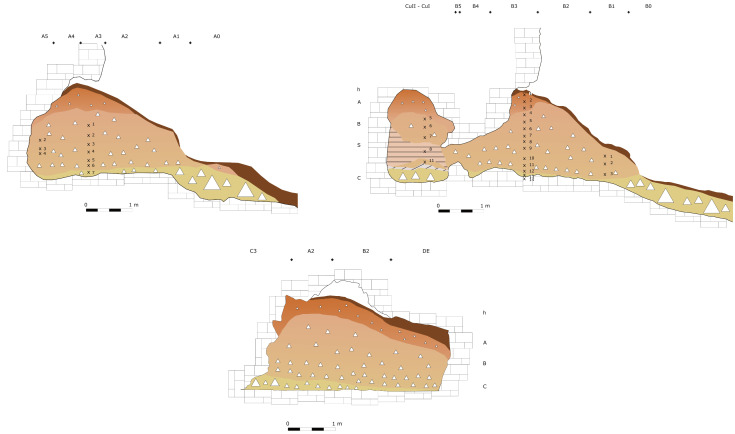
Grotta del Capriolo 1968 redrawn profile sections drawings. Drawing Jacopo Gennai.

In contrast to the relatively comprehensive documentation available for Buca della Iena, the records for Grotta del Capriolo are rather sparse. The excavation diaries are either missing or contain minimal information. However, we do have the benefit of surviving plans and three-section profiles’ drawings that describe the 1968 excavation. Additionally, a few photographs from the 1968 excavation have survived. Both the photographic and graphical documentation align well with the present-day conditions of the site ( Figure 22; Gennai, 2023). Unfortunately, pinpointing the precise extent and location of trench DE remains a challenge. Comparing the 1968 excavation end and the modern state, Trench DE is confined to the small area between trench B and the external wall ( Figure 23; Figure 24; Gennai, 2023). As depicted in the photographs and section profiles ( Figure 14; Figure 25; Figure 26; Gennai, 2023), the distribution of the deposit followed the primary hill slope. Consequently, the highest accumulation of sediment can be observed in sectors A2 and A3, gradually sloping towards the cave walls, particularly evident in trench B. Simultaneously, the deposit conformed to the slope of the main hill, resulting in less sediment in sectors 1, with the highest accumulation occurring in sectors 2 and 3, which are positioned adjacent to the modern dripline. Similar to the situation at Buca della Iena, artefacts report only the spit and sector information, but unfortunately, there is no correlation between spits in different sectors due to the loss of the original fieldwork notebook. The approximate positions of spits are recorded on the trench A and B profile sections. Consequently, the spans and heights of spits based on the topography of the deposit could be calculated, yielding the results illustrated in Table 3.

**Table 3.** Grotta del Capriolo. Correlation of spits among the different sectors.

C1	A1	B1	DE	C3/2/4	A2/3/4	B2/3/4	DE
				1		1	
						2	
				2		3	
				3	1	4	
1	1			4	2	5	
2				5	3	6	
3	2				4	7	A9
4				6	5	8	A10
5	3	1	E1	7	6	9	A11
6		2		8	7	10	A12
7		3	E2	9		11	A13
				10		12	A14
						13	A15
				11		14	

Understanding the spatial organization of the DE trench and its spits is a complex undertaking. The initial excavation height was set at 50 cm, which aligns well with the beginning of layer B and the highest points where artefacts were buried (z). According to notes accompanying the artefacts' list, spits E and D correspond to the lower portion of the deposit and are sedimentologically similar to the lowest layer B and to layer C. Specifically, spits A9 to A15 cover the upper section (z= 47 – 102 cm), while spits D and E encompass the lower section (z= 80 – 125/138 cm). By examining the x and y coordinates of artefacts in spits A and E, the approximate position can be deduced. Spits A9 – 15 and D are situated near sector B2 and can be correlated with spits B2-7 to B2-14 ( Figure 22; Figure 25; Gennai, 2023). Conversely, spits E are located adjacent to sector B1 and can be correlated with spit B1-3 ( Table 3).

## Conclusions

The collaborative efforts invested in digitizing, preserving, and disseminating previously unexplored documentation have significantly advanced the accessibility and transparency within the field of archaeological research. This transparency helps in cultivating an environment of cooperation and knowledge exchange, ultimately proving advantageous for the broader scientific community. Buca della Iena, Grotta del Cervo, and Grotta del Capriolo, although relatively inconspicuous on the international stage, stand as exceptional stratified archaeological sites bearing Mousterian artefacts in Tuscany, offering crucial insights into the last Neanderthal occupation during the Middle to Upper Palaeolithic Transition in Western Italy.

One of the principal impediments to their international recognition has been the absence of a comprehensive description of the archaeological context. Using previously unpublished documentation from these sites has played a pivotal role in addressing this lacuna in our understanding of the sites’ artefact burial context. This approach has facilitated the re-evaluation of established paradigms, enriching the comprehension of these archaeological sites. The incorporation of previously undisclosed field notes, diaries, photographs, and drawings has provided invaluable insights into the intricacies of the excavation procedures, enabling a more precise and nuanced reconstruction of the stratigraphical sequences at these sites.

Regarding Buca della Iena, this reassessment has resulted in the systematic identification of sectors and the subsequent correlation between various spits. The most noteworthy outcomes, however, have originated from the re-evaluation of Grotta del Capriolo, where an understanding of the precise positioning of spits and their associated heights would have remained elusive without access to the original section profiles. This information holds pivotal importance for comprehending the burial context of artefacts, as the artefacts themselves are exclusively marked with references to sectors and spits, without explicit links to the published stratigraphical sequences. Notably, Grotta del Cervo, hitherto relatively unknown to the global scientific community, is now being introduced on an international scale for the first time.

Following the re-evaluation of the three sites’ stratigraphy, ongoing investigations are focusing on the technological attributes of each archaeological assemblage and gaining insights into the site functions. Furthermore, radiocarbon dating has been initiated to refine our chronostratigraphic understanding of the sequences. In summary, the assimilation of previously unpublished documentation will provide the necessary backbone to introduce fresh perspectives on Neanderthal presence in Western Italy and enhance our understanding of the Middle to Upper Palaeolithic Transition.

## Ethics and consent

Ethical approval and consent were not required.

## Data Availability

DARIAH-DE: Underlying data for ‘The Mousterian in North-Western Tuscany: new data from the Piano di Mommio sites - The Tuscan Mousterian’, https://doi.org/10.20375/0000-0011-4996-2 ( Gennai, 2023). This project contains the following underlying data: Figure 1_Gennai_Toscana_Musteriano.tiff (image/tiff) Figure 2_Gennai_Piano di Mommio sites and geology.tiff (image/tiff) Figure 3_Gennai_Mousterian artefacts.tiff (image/tiff) Figure 4_Gennai_Buca della Iena opening.tiff (image/tiff) Figure 5_Gennai_Buca della Iena terrace.tiff (image/tiff) Figure 6_Gennai_Buca della Iena northern profile section drawing 1966.tiff (image/tiff) Figure 7_Gennai_Buca della Iena before excavation in 1966.tiff (image/tiff) Figure 8_Gennai_Buca della Iena bottom of the sequence 1966.tiff (image/tiff) Figure 9_Gennai_Buca della Iena Northern profile after excavation in 1966.tiff (image/tiff) Figure 10_Gennai_Grotta del Cervo before excavation 1988.tiff (image/tiff) Figure 11_Gennai_Grotta del Cervo external area complete excavated sequence.tiff (image/tiff) Figure 12_Gennai_Grotta del Cervo bottom of the sequence inner southern area.tiff (image/tiff) Figure 13_Gennai_Grotta del Capriolo modern view.tiff (image/tiff) Figure 14_Gennai_Grotta del Capriolo during excavation 1968 trench A.tiff (image/tiff) Figure 15_Gennai_Grotta del Capriolo site plan.tiff (image/tiff) Figure 16_Gennai_Grotta del Capriolo transversal section drawing 1968.tiff (image/tiff) Figure 17_Gennai_Grotta del Capriolo trench A profile section drawing.tiff (image/tiff) Figure 18_Gennai_Grotta del Capriolo trench B profile section drawing.tiff (image/tiff) Figure 19_ Gennai_Buca della Iena and Grotta del Cervo redrawn site plan.tiff (image/tiff) Figure 20_Gennai_Buca della Iena redrawn Northern profile sectiion.tiff (image/tiff) Figure 21_Gennai_Grotta del Cervo profile section.tiff (image/tiff) Figure 22_Gennai_Grotta del Capriolo comparison between end of excavation 1968 and modern.tiff (image/tiff) Figure 23_Gennai_Grotta del Capriolo redrawn comprehensive site plan.tiff (image/tiff) Figure 25_Gennai_Grotta del Capriolo end of excavation 1968.tiff (image/tiff) Figure 24_modern niche DE.tiff (image/tiff) Figure 26_Gennai_Grotta del Capriolo sections redrawn.tiff (image/tiff) SI_Gennai_Piano di Mommio.pdf (Text/pdf) bollettino gruppo di ricerche archeologiche Blanc_1_1967.pdf (application/pdf) bollettino gruppo di ricerche archeologiche Blanc_5_1968.pdf (Text/pdf) bollettino gruppo di ricerche archeologiche Blanc_9_1972.pdf (Text/pdf) bollettino gruppo di ricerche archeologiche Blanc_11_1973 .pdf (Text/pdf) bollettino gruppo di ricerche archeologiche Blanc_13_1975.pdf (Text/pdf) Buca della Iena 1966-1973 settori A e B.pdf (Text/pdf) Grotta del Capriolo_diario di scavo Tozzi 1970.pdf (Text/pdf) Grotta del Capriolo_inventario manufatti Fornaciari-Tozzi.pdf (Text/pdf) Grotta del Cervo_1989_Cocchi.pdf (Text/pdf) Buca Iena_Diario Fornaciari trascritto.pdf (Text/pdf) Siti Musteriano Toscana.csv (Dataset/csv) Siti Musteriano Toscana.xlsx (Datasets/xlsx) Data are available under the terms of the Creative Commons Attribution 4.0 International license (CC-BY 4.0)
