# The Mousterian in North-Western Tuscany: publishing fieldwork documentation leads to a new stratigraphical interpretation of the Piano di Mommio sites

**DOI:** 10.12688/openreseurope.17018.2

**Published:** 2024-06-21

**Authors:** Jacopo Gennai

**Affiliations:** 1 Civilisations and Forms of Knowledge, University of Pisa, Pisa, Tuscany, 56126, Italy

**Keywords:** Mousterian, Neanderthals, archaeological excavation, stratigraphy, Italy, fieldnotes

## Abstract

**Background:**

The Mousterian technocomplex is commonly associated with Neanderthals and therefore serves as a proxy for their presence across Europe. Stratified archaeological sites are the most informative because they can yield information about artefacts' spatial distribution and dating. Only a few of the Mousterian sites in Tuscany (Italy) met these conditions and most of these sites are concentrated in the North-Western area, with three specific sites situated in proximity to the village of Piano di Mommio, on the slopes of a small river canyon. Nevertheless, research on the sites stopped early on due to their small extent and complete excavation, which does not allow for additional fieldwork.

**Methods:**

This article presents previously unpublished field notes, reports, and images, which are then correlated with recent archaeological surveys.

**Results:**

This combination of historical and contemporary data aims to provide a more detailed understanding of the context in which the assemblages at these sites were found. The insights gained from this research shed light on the arrangement and positioning of artefacts at these locations, offering valuable information to guide future investigations on the assemblages.

**Conclusions:**

The proposed stratigraphical interpretation adheres to the available information and therefore contributes to a future baseline for new research on the sites and on Neanderthal presence in the area.

## Introduction

### The Mousterian industries

The Mousterian, a lithic industry primarily characterised by flake production, is associated with Neanderthals in Europe ( Higham *et al.*, 2014). Initially defined by Gabriel de Mortillet in 1873, the Mousterian became a fundamental term within the field of Prehistoric Archaeology as the discipline advanced, ultimately becoming often interchangeable with the term Middle Palaeolithic in Europe ( Depaepe, 2020; Monnier, 2006). Spanning from approximately 300/250 thousand years Before the Present (ka BP) to around 40 thousand years calibrated Before the Present (cal BP) ( Depaepe, 2020; Higham *et al.*, 2014; Richter, 2011), the European Middle Palaeolithic period closely aligns with the emergence and evolution of the Neanderthal species ( Arsuaga *et al.*, 2014; Romagnoli *et al.*, 2022). At the core of the Mousterian lies the adoption of predeterminate flaking methods. These facilitated the controlled shaping of end products and, in specific instances, the categorisation of products into distinct technological roles—core-shaping flakes, forming the necessary convexities, and end-products, crafted from these convexities ( Moncel *et al.*, 2020; Moncel *et al.*, 2012; Richter, 2011; Soressi & Geneste, 2011). François Bordes sought to systematise the Mousterian and the broader Middle Palaeolithic framework, resulting in the creation of the first typological list for Lower and Middle Palaeolithic artefacts in 1961, which is largely adopted until nowadays ( Bordes, 2000). This taxonomy contains also non-retouched types, for example, Levallois products. Subsequently, the concept of chaîne opératoire and the integration of lithic technology into lithic artefacts’ studies contributed to a clearer elucidation of the Levallois flaking process ( Audouze & Karlin, 2017; Boëda, 1994). The emergence of the Levallois method is as early as Marine Isotope Stage 12 - 9 (MIS - about 400 – 350 ka BP), with several sites yielding artefacts characteristic of this knapping strategy ( Carmignani *et al.*, 2017; Hérisson & Soriano, 2020; Moncel *et al.*, 2020). From MIS 8 (about 250 ka BP) onwards the method is spread throughout the whole of Europe ( Delagnes *et al.*, 2007; Delagnes & Meignen, 2006; Faivre *et al.*, 2017). In addition to the Levallois method, the Mousterian assemblages revealed other flaking techniques, such as the Quina and the Discoid methods ( Boëda, 1993; Boëda *et al.*, 1990; Bourguignon, 1997; Delagnes *et al.*, 2007; Peresani, 2003). It is worth noting that the chaîne opératoires are not inherently exclusive, with instances of cross-method integration on the same core or branching out in a technological continuum ( Bourguignon *et al.*, 2004; Bustos-Pérez *et al.*, 2023; Çep *et al.*, 2021; Mathias & Bourguignon, 2020). Since the initial work of Bordes ( Bordes, 1953), archaeologists have endeavoured to identify shared typological and technological traits within technocomplexes, which underlie Palaeolithic societies and possibly indicate phylogenetic connections ( Reynolds & Riede, 2019; Riede *et al.*, 2020). For the European Middle Palaeolithic, the prime focus of this endeavour has centred on southwestern France due to the abundance of sites across different periods ( Delagnes & Rendu, 2011; Faivre *et al.*, 2017; Jaubert *et al.*, 2011). However, the identified technocomplexes have been subject to diverse debates. Binford, for instance, favoured a functional interpretation over Bordes' cultural perspective ( Binford & Binford, 1966; Binford & Binford, 1969). Subsequently, as researchers scrutinised the concept of technocomplexes within the context of global variation, questions arose concerning their significance ( Dibble, 1991; Monnier & Missal, 2014; Shea, 2014). Even assuming that technocomplexes are real societal entities, the cultural sequence in southwestern France has been a subject of intense discussion. For instance, Bordes and subsequent scholars recognised a connection between the Mousterian of Acheulean Tradition – A (MTA – A), the Mousterian of Acheulean Tradition – B (MTA – B), and the Chatelperronian, as these assemblages often were found in this stratigraphical order and had insights of gradual techno-typological evolution ( Roussel & Soressi, 2014). More recently, this lineage has been re-evaluated considering the potential non-existence of the MTA – B (conceived from unrecognised lithic artefacts mixing in palimpsests) and the identification of distinct final Mousterian technocomplexes between MTA – A and the Chatelperronian ( Gravina & Discamps, 2015; Jaubert *et al.*, 2011).

### The Mousterian sites in North-West Tuscany

**Figure 1.**
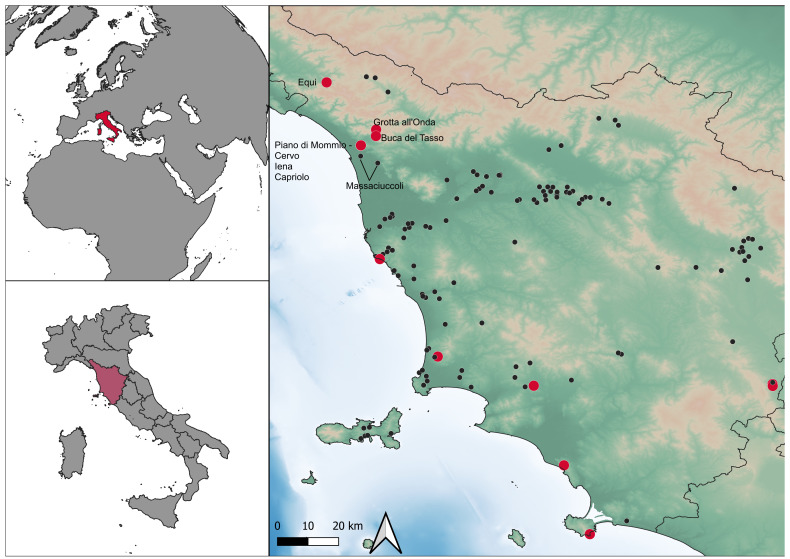
Location of Mousterian sites in Tuscany and sites mentioned in the text. Red: stratified sites.

The Mousterian is distributed across the whole Italian Peninsula, revealing clusters of sites, particularly referring to stratified assemblages ( Aureli & Ronchitelli, 2018; Palma di Cesnola, 2001). Notably, the northwestern area of Tuscany (Italy – Figure 1) stands as one of the prominent clusters of stratified sites ( Gennai, 2024). Most of the discoveries in the area date to the earliest endeavours of Prehistoric Archaeology in Italy ( Blanc *et al.*, 1935; Branchini, 1928; De Stefani, 1915; Graziosi, 1944; Mochi, 1915; Palma di Cesnola, 1970; Regnoli, 1867; Rellini, 1916). In order of discovery, the sites yielding Middle Palaeolithic assemblages that emerged during this pioneering era are Grotta all’Onda, Tecchia d’Equi, and Buca del Tasso.

**Figure 2.**
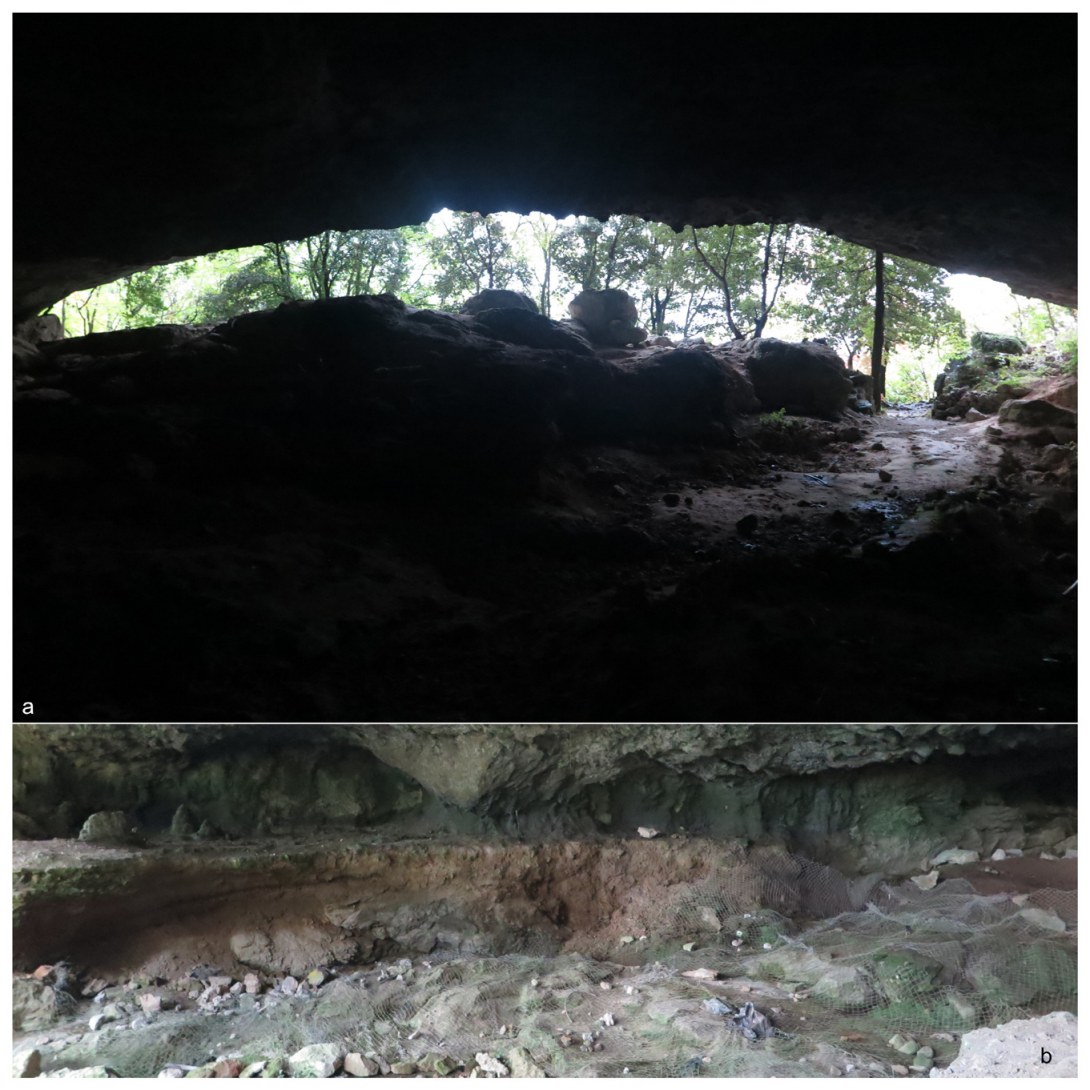
Grotta all’Onda site ( **a**) and the remaining sediments on the western wall ( **b**). Photos Jacopo Gennai.

Grotta all’Onda (located near Casoli, Camaiore, Italy) stands as one of the earliest Prehistoric sites to be surveyed and excavated in Italy. On and off-site investigations started in the latter half of the 19 ^th^ century and ended in 2005, led to the current understanding of the site ( Berton *et al*., 2005; Berton *et al*., 2003a; Campetti *et al.*, 2001; Fornaca-Rinaldi & Radmilli, 1968; Graziosi, 1944; Mochi & Schiff-Giorgini, 1915; Regnoli, 1867). The site is a big karst cave (approximately 60 by 40 m – Figure 2). The various excavation campaigns focussed on the western exterior and middle cave sector and nowadays only some sediment is found attached to the cave wall ( Figure 2, Campetti *et al.,* 2001; Gennai, 2024a). The stratigraphical sequence, as per the last research, comprises five different moments of sedimentation separated by erosions, flowstones, and sedimentation hiatuses providing some evidence for living floors ( Campetti *et al.,* 2001). From bottom to top:

- Basal flowstone: dated with 230Th/232Th method to 174.0 ± 8.2 ka ( Berton *et al.,* 2003a)
- Sedimentation sequence 1: it consists of a fine yellow clay subsequently covered by a clasts-supported breccia deposit
- Palaeosurface: it consists of brown-red loamy sediment with black lenses, within this sediment there are rare Mousterian artefacts. This could be the Layer 5 - Foyer C/D reported in past excavations ( Graziosi, 1944; Mochi & Schiff-Giorgini, 1915). Layer 5 – Foyer C/D rested upon some stalagmite deposition, subsequent 230Th/238U dating of a sample led to 39.3 ± 3.2 ka dating ( Fornaca-Rinaldi & Radmilli, 1968). This date cannot be reproduced, hence later investigations proposed this stalagmite deposition was localised and not related to the basal flowstone. Two cave bear bones retrieved in this level date to 37,139 ± 530 (sublevel 7j3, LTL1026A - 68.2% probability: 42,076 – 41,256 cal BP; 95.4% probability: 42,462 - 40,724 cal BP) and 36,996 ± 565 (sublevel 7j5, LTL1025A - 68.2% probability: 42,016 – 41,096 cal BP; 95.4% probability: 42,402 – 40,498 cal BP) ( Molara, 2009; Moroni *et al*., 2019). No specifications on the dating methods, except being radiocarbon, are available. Cave bear is the most frequent faunal remain, even with young individuals, therefore the site is interpreted as a hibernation den, and a relationship with the human occupation is unclear ( Molara, 2009).
- Sedimentation sequence 2: it consists of clay deposits related to a high-energy water circulation.
- Sedimentation sequence 3: massive silt deposition intercalated with breccia fine levels. Within the breccia levels there rare lithic artefacts.
- Sedimentation sequence 4: another moment of high water energy circulation bringing silt and chalky sediments.
- Upper flowstone: the previous sedimentation sequence culminates in this thick flowstone. Dated with 230Th/232Th method to 10.7 ± 0.2 ka, it is as a demarcation between the Pleistocene and the Holocene ( Berton *et al.*, 2003a).
- Sedimentation sequence 5: lying abruptly on the upper flowstone, it consists of dark, massive clay deposit with numerous findings associated with the Late Neolithic and Copper Age.

Outside of the sequence and the cave's modern dripline, in 2002 an intact hearth associated with numerous lithic artefacts, techno-typologically attributed to the Epigravettian, shows an occupation during the last stages of the Palaeolithic ( Berton *et al.,* 2003b).

**Figure 3.**
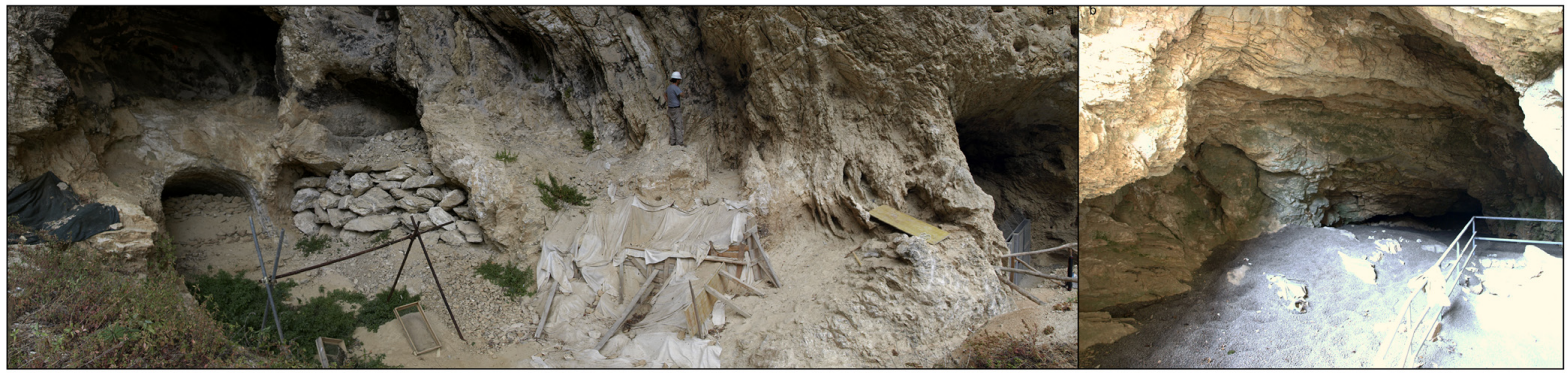
Tecchia di Equi site: the shelter ( **a**) and the inner cave area ( **b**). Photos courtesy of SABAP Massa-Carrara/Lucca.

The Tecchia d’Equi (Equi Terme, Italy), an important archaeological and paleontological site in Tuscany, consists of two contiguous parts: the Tecchia (a shelter) and the Sala (inner cave – Figure 3, Gennai, 2024a).

Systematic excavations from 1911 to 1917 removed sediments previously covering the inner cave ( De Stefani, 1923; De Stefani, 1916a; De Stefani, 1916b; De Stefani, 1916c; De Stefani, 1915; Rellini, 1924; Rellini, 1916). While the shelter area showed occupation during historical times, the inner cave had Prehistoric settlements ( Bigagli *et al*., 2018; De Stefani, 1916a). During these excavations, the whole cave's external area (Sala) was investigated and freed from sediments. The stratigraphical sequence of the cave unveiled an intricate alternation between limestone breccias and thin layers of fine reddish sediment, which turned yellowish at the end of the sequence. Two main levels had human occupation. The first one was at 3.75 m depth, it extends from the exterior part of the cave to the inner part, it is sub-horizontal and contains darker lenses ( De Stefani, 1916c). The second one was at 5.10 m depth, totally within the inner cave and consisting of a yellow sandy deposit ( De Stefani, 1917). The first occupation level had flint artefacts attributed both to the Copper Age and Palaeolithic (mostly Mousterian), and the second one just Mousterian artefacts. The first occupation level contained also human remains ( *H. sapiens*) ( De Stefani, 1916c). Nevertheless, the occurrence of mixed archaeological materials and faunal remains (such as Pleistocene carnivores) is reported between the two levels ( De Stefani, 1917; Ghezzo *et al*., 2014). The mixing of artefacts, human remains, and faunal remains belonging to different ages shows a complex depositional history that was impossible to disentangle at the time. An analysis of 1911 – 1917 lithic findings concluded that they are mostly Mousterian ( Branchini, 1928). In 1931 new excavations explored the shelter deposits and recovered Mousterian artefacts from a reddish clay level ( Blanc *et al.*, 1935). The artefacts are now lost ( Palma di Cesnola, 1970). After World War II, research was carried out intermittingly, but mostly took care of salvaging deposits after looters’ damages or was spatially limited to the deeper areas of the cave: no new stratigraphical details were achieved and most of the findings were considered part of reworked deposits ( Ambrosi, 1959; Ambrosi, 1981; Ambrosi & Feola, 1951; Graziosi, 1969; Graziosi & Guerri, 1972; Guerri, 1982; Guerri, 1980). After a long hiatus, research started again in 2009, when the Soprintendenza per i Beni Archeologici della Toscana found *in-situ* deposits both in the Tecchia and in the Sala ( Iardella *et al.*, 2012). The excavation focused on the western wall of the shelter and revealed a deposit characterised by loess sediments. Within the upper portion of the deposit, Mousterian artefacts, fauna remains, and dispersed charcoals were discovered, often associated with detached boulders from the shelter's roof ( Figure 3 - Gennai, 2024a; Iardella *et al.*, 2012). However, this portion of the deposit up to the floor level, exhibited varying degrees of reworking ( Iardella *et al.*, 2012). The dating of charcoal samples from the most intensively occupied level exceeds the radiocarbon limit ( Bigagli *et al.*, 2018; Palchetti, 2015). Subsequent investigations in 2012 extended to the outermost part of the inner cave (Sala 1 – Figure 3, Gennai, 2024a), revealing yet another *in-situ* deposit. This deposit was defined by loess sediment lenses intermingled with detached boulders and breccias from the cave roof. Mousterian artefacts and charcoal were also uncovered within this context, dated to 43700 ± 1900 BP and 44000 ± 2200 BP, effectively aligning the deposit with MIS 3 (about 60 – 30 ka cal BP ( Sanchez Goñi & Harrison, 2010)) ( Bigagli *et al.*, 2018; Bigagli *et al.*, 2013). Despite the efforts, it is impossible to correlate the new excavations with the 1911 – 1917 ones ( Bigagli *et al.*, 2013; Ghezzo *et al*., 2014). Further developments in 2014 involved the re-excavation of the transitional zone connecting the exterior section of the cave (Sala 1) to the previously explored interior area from the 1980s. This decision was prompted by the recognition of additional *in-situ* deposits. The sediment composition in this area retained its loess nature mixed with breccia debris, and the archaeological findings remained consistent with the Mousterian ( Palchetti, 2015).

**Figure 4.**
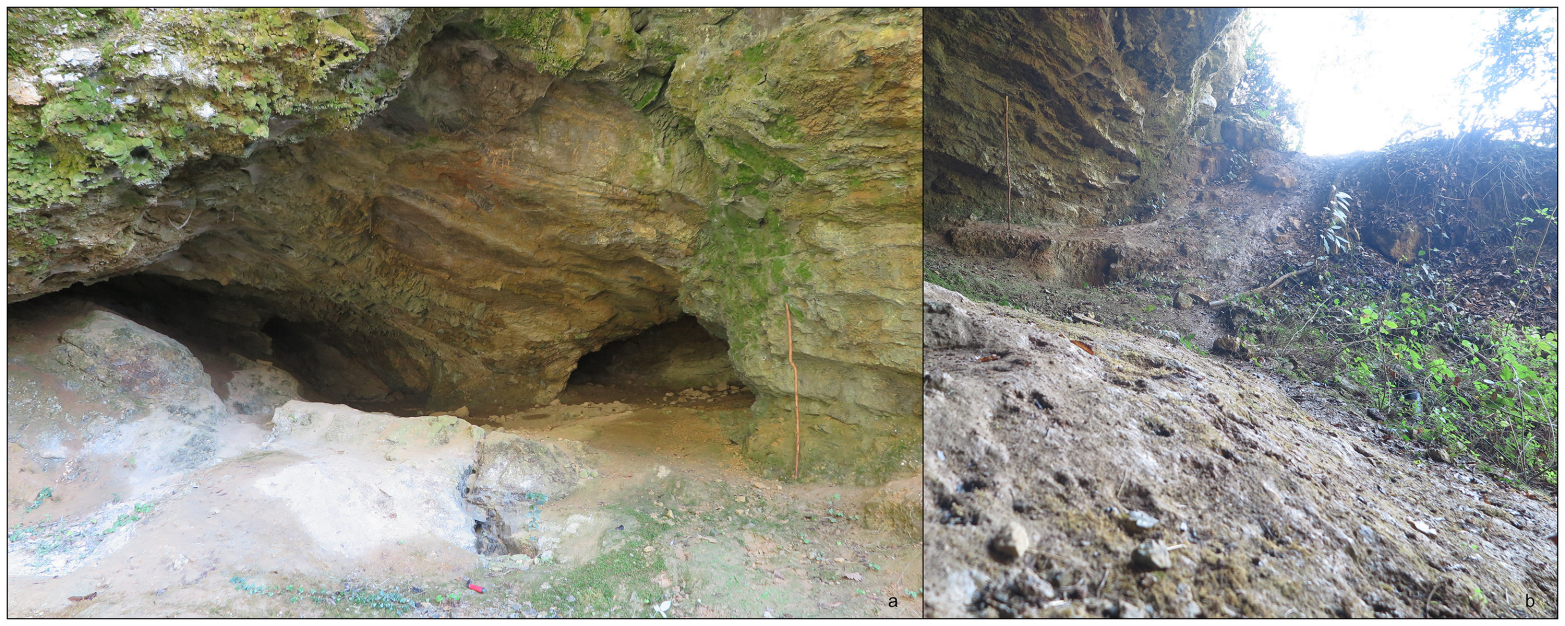
Buca del Tasso site nowadays: inner part ( **a**) and external talus ( **b**). Photos Jacopo Gennai.

Buca del Tasso (Metato, Camaiore, Italy – Figure 4; Gennai, 2024a) was discovered during an archaeological survey in 1919 ( Puccioni, 1922). Between 1919 and 1922 what appeared only a shelter revealed a small cave ( Figure 4) ( Puccioni, 1922). The excavation yielded a thicker deposit in the external area and only the top part of the stratigraphical sequence was found in the inner cave. From the bottom to the top:

- Layer C: it was found only in the exterior area and it consisted mostly of clay deposits subdivided into three horizons, C’’’, C’’, and C’. C’’ corresponds to a collapse of the cave vault. C’’’ was resting upon the bedrock. Layer C yielded eight (8) Mousterian artefacts in total.
- Layer B: it consisted of a clasts-supported breccia level, resting upon the bedrock in the inner cave.
- Layer A: it consisted of silty and loose deposits, divided into a darker upper horizon and a reddish lower one. It yielded fifty-eight (58) Mousterian artefacts ( Palma di Cesnola, 1970; Puccioni, 1926). Furthermore, two diaphyses were reported as human ( Puccioni, 1922). Further investigations reduced the sample to only one; it is a femur portion and it belonged to a 7 – 9 years old child, currently, it is the only known Neanderthal fossil in Tuscany ( Alciati *et al*., 2005).

No dating is available for Buca del Tasso, faunal remains and typological considerations made on the artefacts might imply a Late Pleistocene age, maybe early MIS 3 ( Alciati *et al*., 2005).

Palaeolithic artefacts, among those Mousterian ones, occurred with the commercial sand extraction in various localities of the Massaciuccoli Lake (Viareggio – Massarosa, Lucca, Italy) ( Blanc, 1937; Blanc, 1934; Fornaciari, 1966). Depending on the area of extraction, they could originate from 12 to 26 m depth, but ascertaining the real burial context is impossible at the current stage ( Blanc, 1934; Fornaciari, 1966).

**Figure 5.**
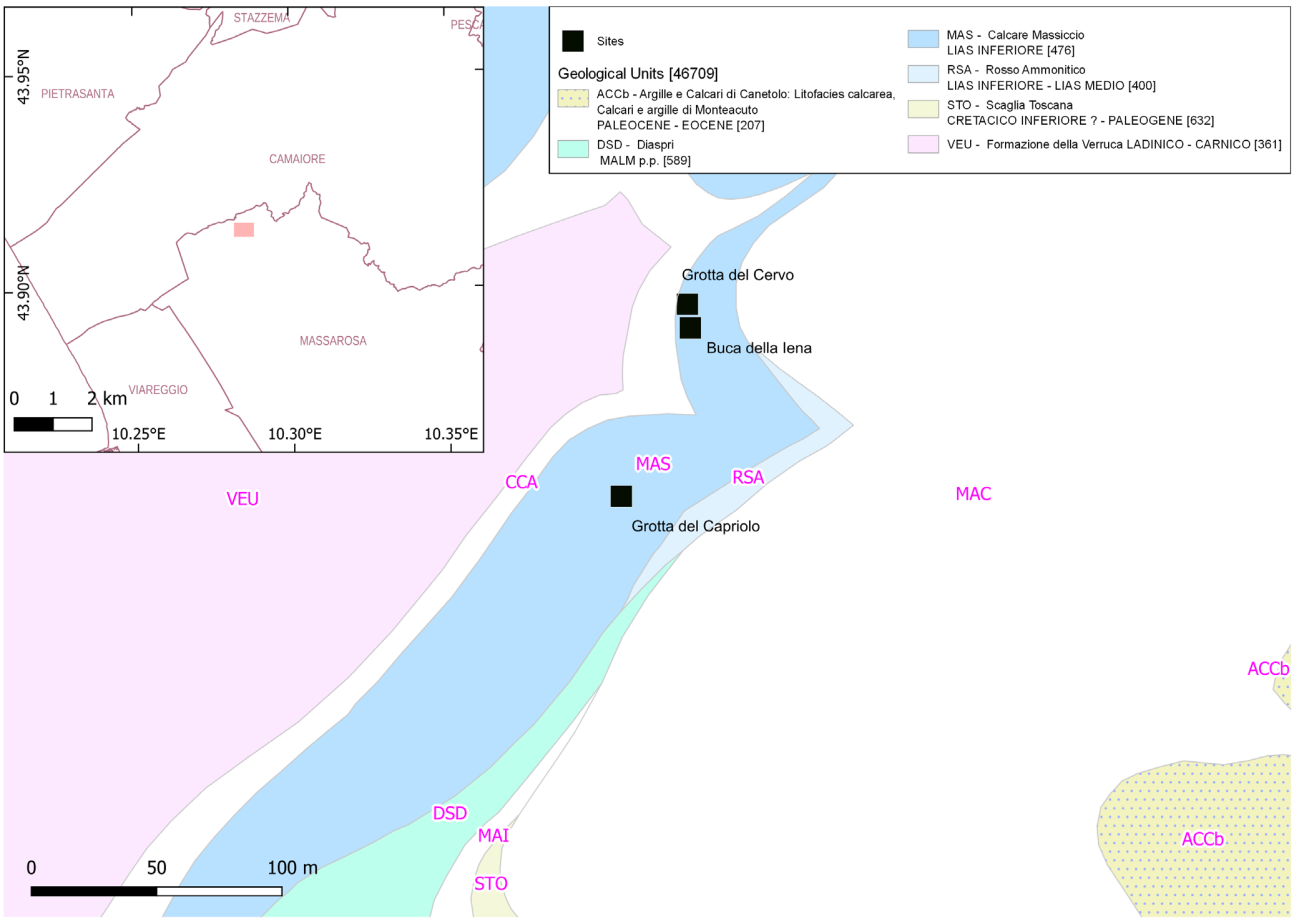
Location of Piano di Mommio sites within their geological context (DB geologico 1:10.000 Regione Toscana).

**Figure 6.**
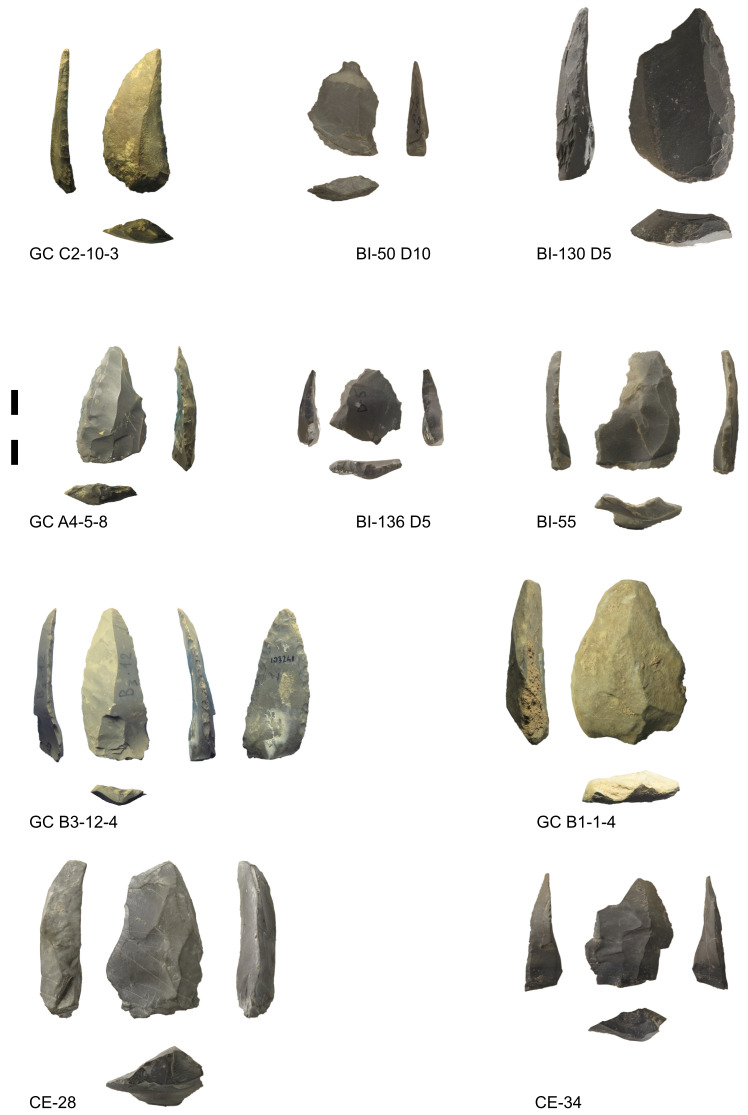
Some of the Mousterian artefacts from Buca della Iena (BI), Grotta del Capriolo (GC) and Grotta del Cervo (CE). Photos Jacopo Gennai.

The available information from Grotta all’Onda, Equi, Buca del Tasso, and Massaciuccoli heavily influenced discoveries and investigation of Buca della Iena, Grotta del Capriolo (in the second half of the 1960s’ - Fornaciari, 1966; Fornaciari, 1977; Gennai, 2024; Pitti & Tozzi, 1971), and Grotta del Cervo (early 1990s’ - Cocchi, 1989; Cocchi-Genick, 1993; Cocchi-Genick, 1992; Cocchi-Genick, 1989; Gennai, 2024). Given the geographical proximity, the sites have assimilated within a coherent development of Neanderthal occupation of North-Western Tuscany. For instance, the lower levels of Grotta del Capriolo were associated with Layer A of Buca del Tasso, due to the higher frequency of elongated artefacts. Grotta all’Onda, Equi, Buca della Iena, Grotta del Cervo, and the upper levels of Grotta del Capriolo were associated together in the so-called “Alpine Mousterian”, rich in denticulates ( Cocchi-Genick, 1993; Palma di Cesnola, 1970; Pitti & Tozzi, 1971). Buca della Iena, Grotta del Capriolo, and Grotta del Cervo are close to each other located within a limestone outcrop next to Piano di Mommio (Massarosa, Lucca, Italy) ( Figure 5; Conti *et al.*, 2010; Gennai, 2024). Dating of a flowstone underlying the Buca della Iena assemblage provided a *terminus post quem* for the artefacts, <41 ka BP ( Fornaca-Rinaldi & Radmilli, 1968), and the sites are generally believed to belong to the last Mousterian manifestations ( Figure 6; Gennai, 2024). More recently, the presence of denticulates was downplayed in favour of a more technological approach, but the sites are still considered largely contemporary, belonging to the MIS 3 and the last Neanderthals in the area ( Dini & Koehler, 2009). Despite the relatively small assemblages, short stratigraphical sequences and limited site extent, Buca della Iena, Grotta del Capriolo, and Grotta del Cervo are important to the understanding of the last Neanderthals’ survival in Central Italy due to the scarcity of stratified sites. Nevertheless, details are difficult to obtain because publications are dispersed in various notes and local papers in Italian language.

### Research objectives

The research for the original documentation began with the primary goal of determining the stratigraphical provenance of the artefacts. These artefacts raised discrepancies with their published provenance details ( Fornaciari, 1966; Pitti & Tozzi, 1971). The main problem is that the stratigraphical sequence is presented according to lithostratigraphical layers (see the Results section below), but the inking on findings does not mirror this. The Buca della Iena findings show, variably, the site initials, a letter (A, B, C, D, E, F), and a number (1 – 15). The Grotta del Capriolo findings show a letter (A, B, C), followed by a subscript number (1 – 5) and a number (1 – 14). Some Grotta del Capriolo lithic artefacts show a sequential number (i.e. 397) or a sticky paper label reporting “Ca” and a sequential number. This makes it impossible to understand artefacts’ stratigraphical positions and to reproduce published results. Additionally, some of the lettering and numbering are identical to published stratigraphical sequences, leading to misunderstandings. After this assessment, it became imperative to source the original documentation and ascertain the genuine stratigraphical origins of these artefacts.

## Methods

The investigation encompassed multiple research avenues.

Most of the original documentation or duplicates were procured through collaboration with the original excavators, specifically Prof. Gino Fornaciari and Prof. Carlo Tozzi. These documents included two profile sections of Buca della Iena, three profile sections of Grotta del Capriolo (Trench B, Trench A and the transversal section cutting the whole deposit at the modern dripline), and the comprehensive plan of the 1968 Grotta del Capriolo excavations. In addition, they provided the Buca della Iena 1966 excavation field notes and the 1970 Grotta del Capriolo excavation field notes. Prof. Fornaciari provided some unpublished excavation pictures for Buca della Iena (1966 excavations) and Grotta del Capriolo (1968 excavations).

Concurrently, the literature search extended to local publications. The Soprintendenza Beni Archeologici e Paesaggistici (SABAP) Massa-Carrara/Lucca possessed additional resources, encompassing the Bulletin of Information and the Activities Diary of the local archaeologists' society, which had conducted excavations at Buca della Iena and Grotta del Capriolo. This source also provided the Grotta del Cervo 1989 post-excavation report, along with the site plan, profile sections, and the list of spits. Furthermore, the Museo Civico Archeologico e dell’Uomo “Carlo Alberto Blanc” in Viareggio housed photographs of the Grotta del Cervo excavation, in addition to the archaeological material.

Given the composite nature of the documentation, sites needed new surveys to compare with the documentation and address any gaps. This entailed multiple site visits to obtain fresh photographs, and coordinates, and to compare or integrate existing documentation. This process was particularly significant for Grotta del Cervo and Buca della Iena, which are both on the same private property.

For Buca della Iena no stratigraphical profile survived. Hence, I integrate the fieldnotes, the available pictures, the surviving profile drawing and the surveying of the modern terrace to build up a spatial distribution of excavation sectors and a more accurate profile drawing. Also, field notes are precious in reconstructing how the deposit looked like and the sedimentary correlations between spits of different sectors.

For Grotta del Capriolo no field notes survived, except some brief notes from the 1970 excavations. Thanks to the 1968 excavations pictures, plan, and the comparison with the modern survey of the site, I can reconstruct the location of the 1970 excavations (unavailable in previous published work). Information about arbitrary spits location is mostly drawn from the 1968 unpublished profile drawings, this led to the newly proposed correlation of spits from different excavation sectors. Correlation between 1968 and 1970 spits is drawn upon the reconstructed location of the 1970 trench and three-dimensional coordinates recorded for some of the 1970 artefacts.

For Grotta del Cervo, I limited myself to making unpublished documentation available.

## Results

### Buca della Iena

**Figure 7.**
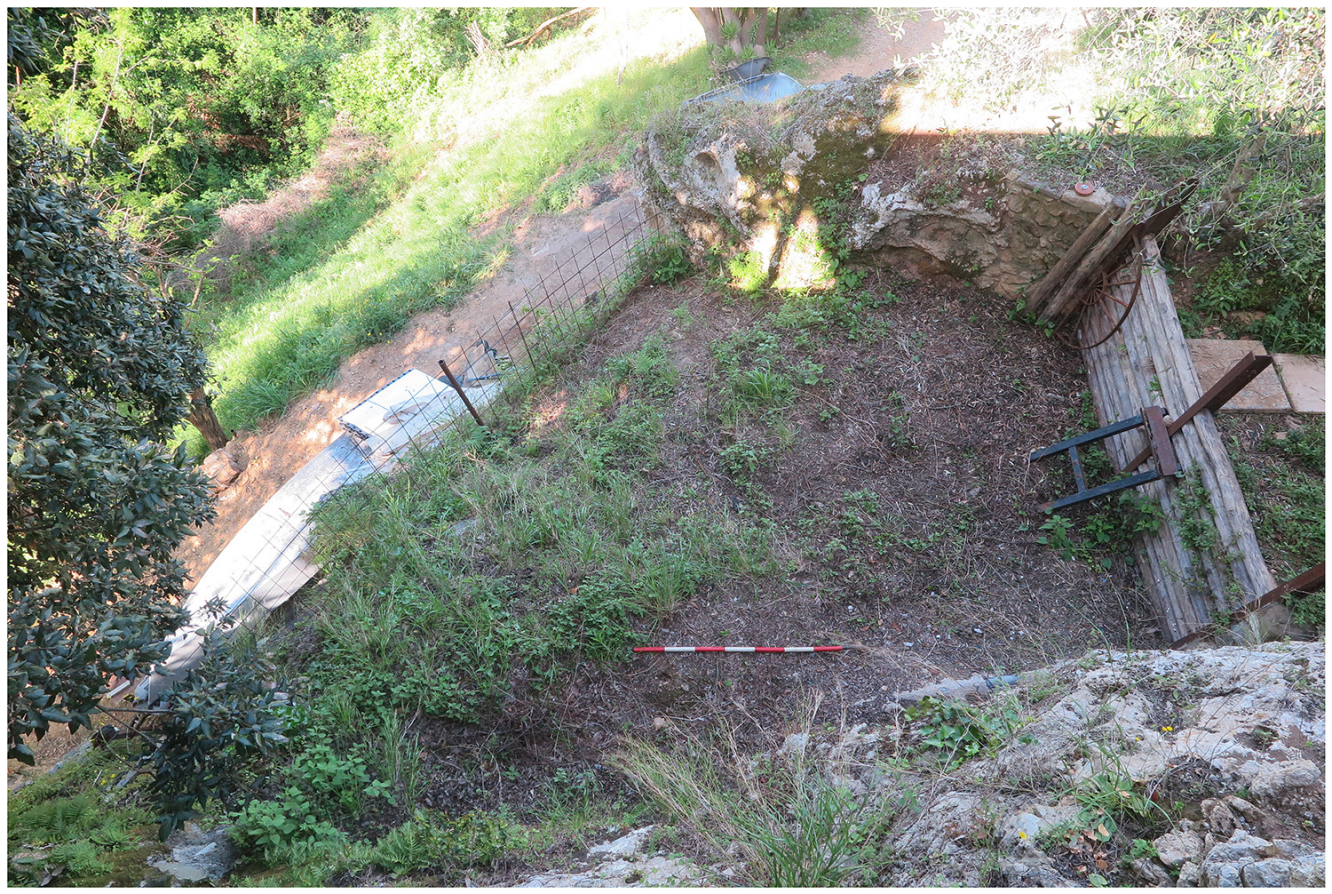
The terrace in front of the Buca della Iena. The limestone boulder ends it. Photo Jacopo Gennai.

**Figure 8.**
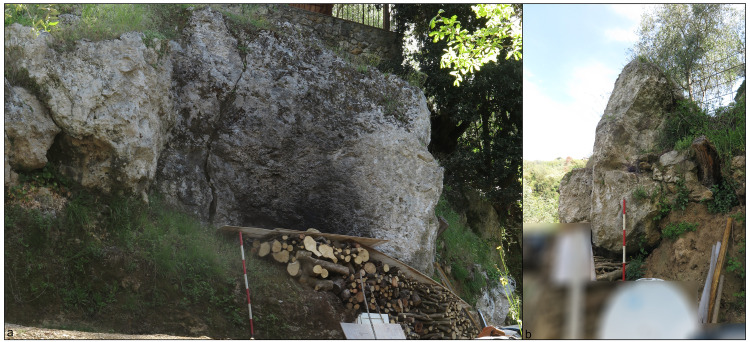
Buca della Iena modern terrace from below and boulder picture. Front of the terrace illustrating the boulder extent ( **a**), and thickness ( **b**). notice the yellow loamy sediment at the bottom. Photo Jacopo Gennai.

**Figure 9.**
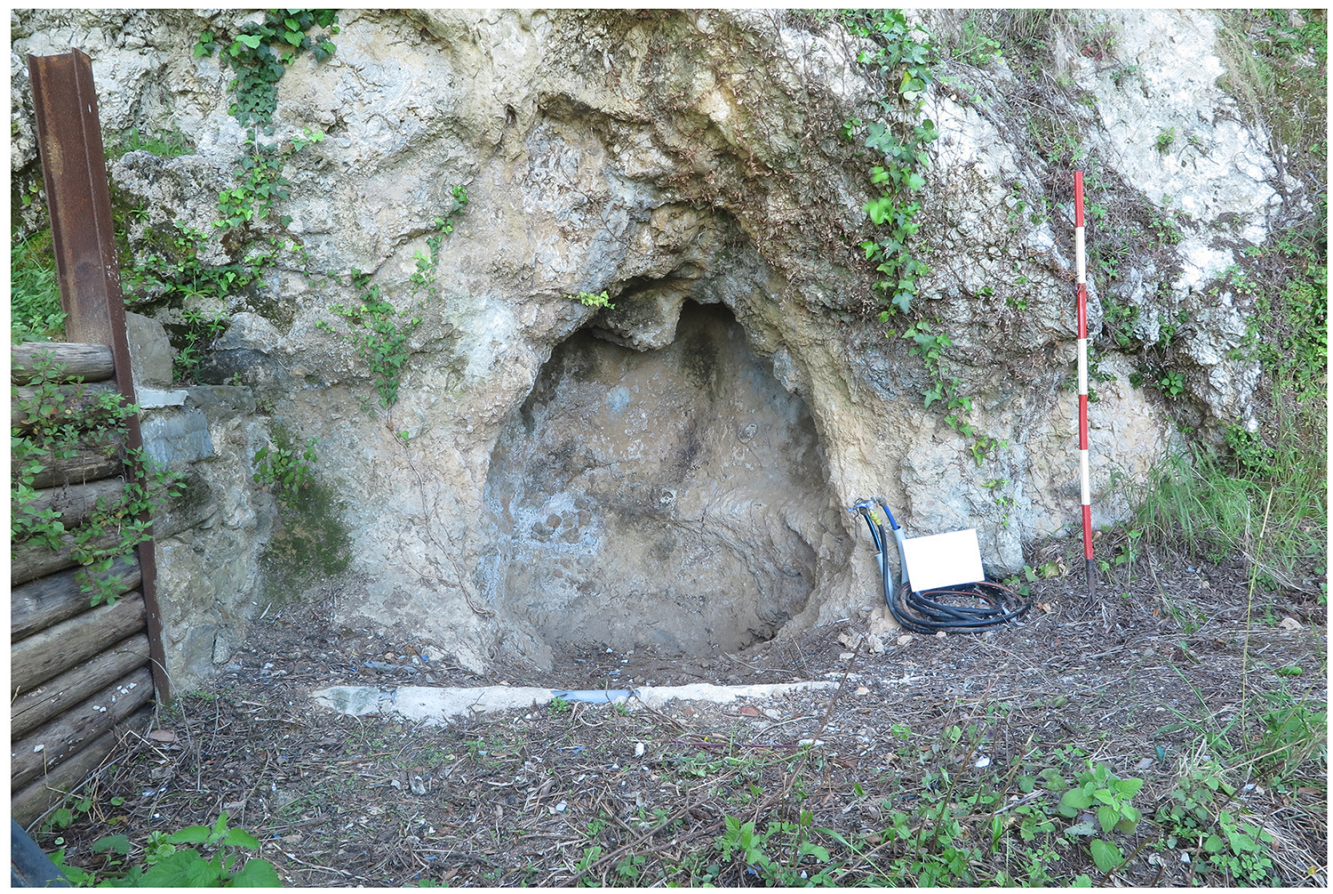
The Buca della Iena niche in the limestone wall. Photo Jacopo Gennai.

The site is located on a limestone outcrop which is isolated by two narrow canyons on the southern and northern sides, nowadays it faces west-southwest (43.9142645 N, 10.2850590 E; Figure 5; Gennai, 2024). The slope was modified by terracing to facilitate farming and one of these terraces took advantage of the extent between the limestone natural step and two limestone boulders ( Figure 7; Figure 8; Gennai, 2024; Gennai, 2024a). Nowadays the site consists only of an empty, small niche in the limestone wall, measuring around 2 m in length 1,7 m in width and 1,85 m maximum height ( Figure 9; Gennai, 2024).

The site was discovered on the 17th of May 1966 and interpreted as a collapsed cave. A small test pit yielded bones of Pleistocene fauna in light-coloured clay underneath the humic level. On the 28th of May, a second test pit was dug, next to the previous one, discovering analogous findings. The systematic excavation started on the 6th of July of that year and lasted until the 20th of July ( Gennai, 2024; Grifoni Cremonesi, 1971; Radmilli, 1966). The following year the Institute for Human Palaeontology of the University of Pisa sampled the remaining stratigraphical sequence to ascertain the sedimentological sequence ( Gennai, 2024; Pitti & Tozzi, 1971; Radmilli, 1967). After recognising damages to the site, the Gruppo di Ricerche Preistoriche ed Archeologiche “Alberto Carlo Blanc” dug the remaining deposit to recover and salvage the archaeological evidence. These excavations took part in the northern sector of the site on the 18th of June 1972, 25th of June 1972, 23rd of July 1972, 27th of August 1972 and 8th of April 1973 ( Gennai, 2024).

**Figure 10.**
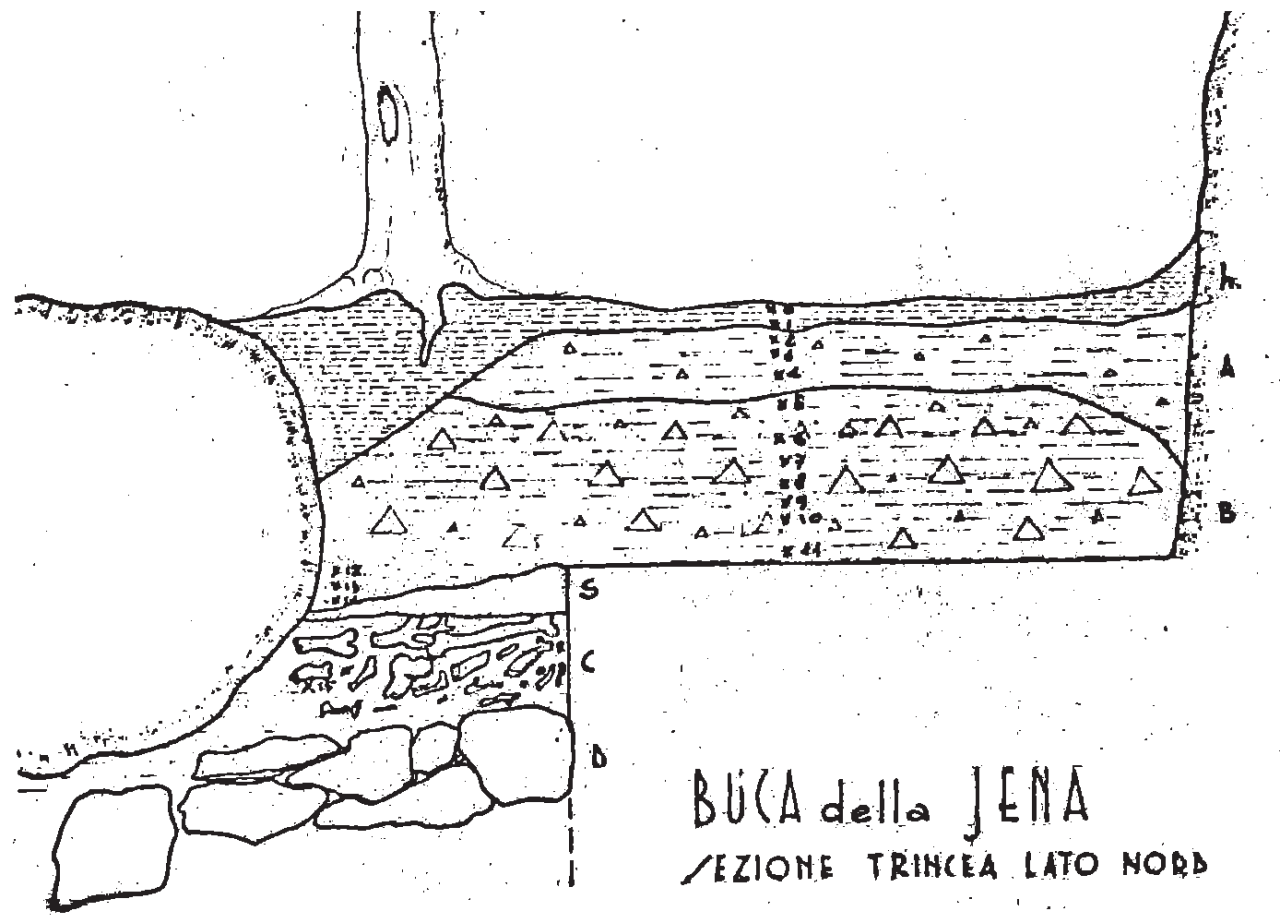
Buca della Iena 1966 northern profile section original drawing. Courtesy of Gino Fornaciari.

According to the original field notes ( Gennai, 2024), the systematic excavation proceeded to open subsequent trenches on the terrace. While the position of these trenches (named sectors in the field notes) can be deduced from field notes, their extent is not recorded. The deposit sequence was explored through arbitrary spits, and sedimentological similarities were used to correlate them to each other. A total of fifteen spits are mentioned, starting from the number 0. Each spit measured roughly 10 to 20 cm, except the last one (no. 15) measuring 50 cm ( Figure 10; Gennai, 2024). The excavated deposit reached 2,75 m depth from the floor level ( Fornaciari, 1966).

The first investigated sectors were A and B. The field notes mention four spits for sector A, two spits for sector B, and a total one-metre depth for both sectors. Spit 2 in Sector A and Spit 1 in Sector B contain reddish clay sediment. Spits 3 in Sector A and 2 and 3 in Sector B are clasts-supported. Spit 4 in Sector A is barely recorded, while in Sector B is mentioned only as containing hyenas’ occupation and without traces of the flowstone. Artefacts attributed to the Mousterian were found in the two upper spits, associated with Pleistocene fauna like cave bear ( *Ursus spelaeus*), while the lower spits only yielded Pleistocene fauna.

Sector C and Sector D followed. Sector D was recorded between Sector C on the left and Sector A on the right. There are thirteen recorded spits in D, while C consists of twelve spits. The two sectors are divided by an olive tree, whose roots and agricultural works highly disturbed the sediments until spit 3. In spit 2 a diagnostic Gravettian or Epigravettian (Upper Palaeolithic) backed point and additional volumetric small blades were found. The following spits showed a progressive increase of limestone fragments and hardening of the sediments, culminating in the appearance of flowstone starting in spits 11 and 12, depending on the sector. The artefacts were all attributed to the Mousterian, and they were mixed with Pleistocene fauna like hyena, cave bear, horse, and rhino. Sector D’s spits are correlated with Sector A’s ones in the following manner:

- Sector D spits 7 and 8 corresponding to Sector A spit 1.
- Secor D spit 10 is corresponding to Sector A spit 3.
- Sector D spit 11 is corresponding to Sector A spit 4.

**Figure 11.**
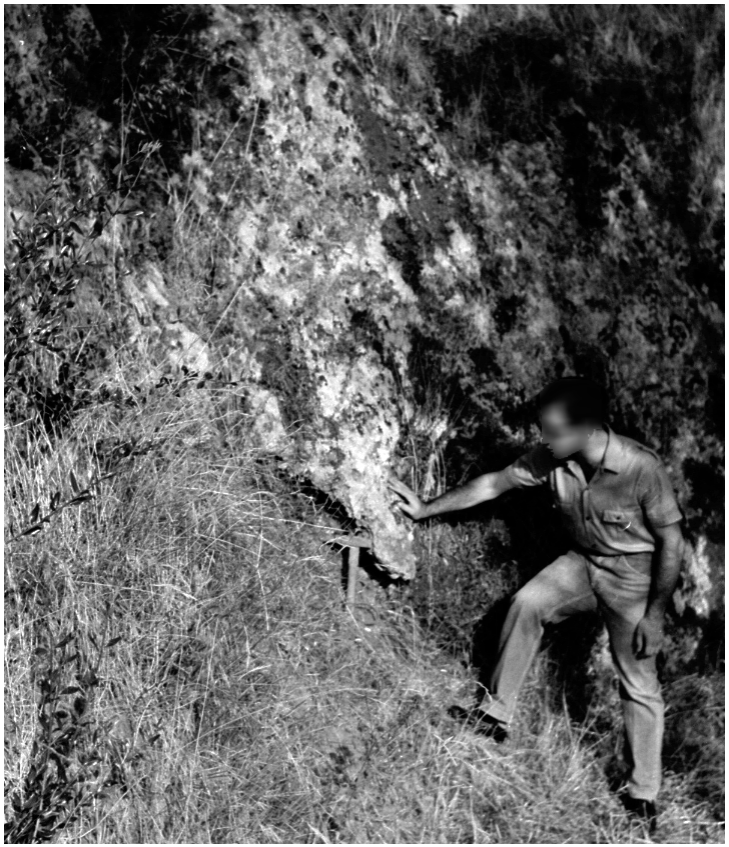
The Buca della Iena before excavation in 1966. Photo courtesy of Gino Fornaciari.

Sector E corresponded to the area of the niche in the limestone wall, which was completely covered before excavation ( Figure 11; Gennai, 2024). It consisted of seven spits, the first one being correlated with spit 5 of sectors C and D. As sediments probably sloped and compacted inside, the sedimentological sequence has less resolution. Hence, spit X corresponds to spits 8, 9, and 10 in sectors C and D. As in the other sectors, sediments transitioned from loose clay to a more cemented one. Deeper and more inside the niche, the deposit shifted to a clast-supported sediment with middle-sized stones. Also, sector E yielded Mousterian artefacts mixed with Pleistocene fauna, mostly cave bear and hyaena.

**Figure 12.**
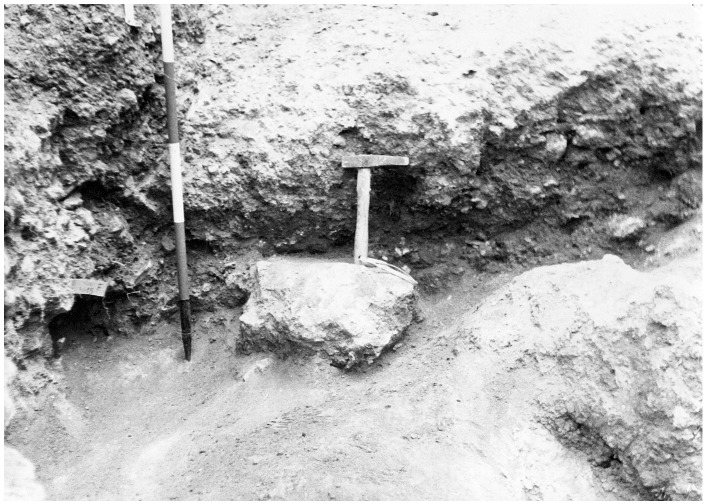
The bottom of the Buca della Iena sequence after excavation in 1966, flowstone closeup. Photo courtesy of Gino Fornaciari.

Finally, sector F was dug. The sector was heavily impacted by an olive tree and by the earliest test-pits meaning that only a small portion of deposit was available for excavation. The upper part of the already excavated sector, F1, showed reddish clay soil, corresponding to the upper spits in Sectors C and D. This witnesses a sloping downwards of the sediments, at least in this excavation area. Sector F consists of eight spits, starting with spit 7 and ending with spit 15, the lowest in the whole excavation. Therefore, sector F is likely located on the slope of the terrace and partially limited by a big limestone boulder, nowadays at the western edge of the terrace. The deposit is similar to that uncovered in the other sectors, increasing in clastic content toward the bottom and hardening progressively. The flowstone is reached in spit 14. The flowstone was noticeably thinner on the front of the spit. Underneath the flowstone, whose lower end is encasing fauna bones, the sediment changes in light-coloured clay with abundant Pleistocene fauna, mostly hyaena and horse, and devoid of any artefacts ( Figure 12; Gennai, 2024). Underneath the clay, there were big blocks interpreted as the bedrock. Sector F is correlated with Sector B spits. Sector F spits 13 and 14 correlate with Sector B spit 2.

**Figure 13.**
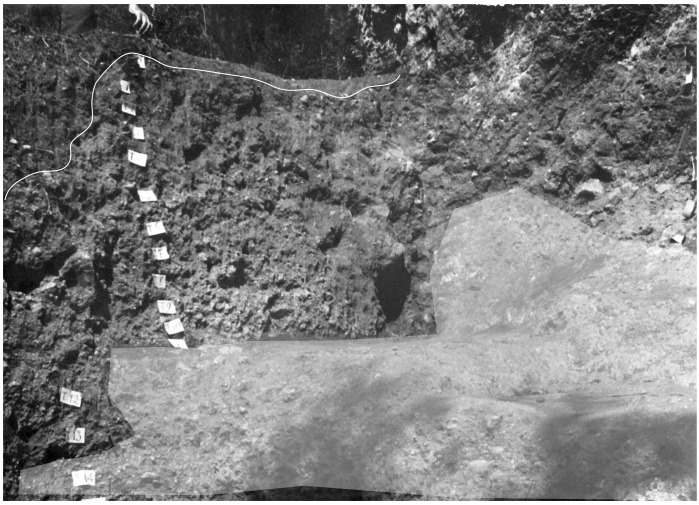
The Buca della Iena northern profile section after excavation in 1966. White line: disturbed sediment, White area: flowstone. Photo courtesy Gino Fornaciari, modified by Jacopo Gennai.

Therefore, the deposit is interpreted as having an upper humic level covering silty reddish-brown sediments with a higher content of limestone and progressive hardening towards the bottom. The flowstone is a clear break in sedimentation because underneath it the sediment is changing in colour, yellowish, and texture, loamy ( Fornaciari, 1966). The subsequent analysis of the sequence by the University of Pisa confirmed the excavators’ impressions ( Pitti & Tozzi, 1971). Both publications ( Fornaciari, 1966; Pitti & Tozzi, 1971) reported the thickness and the sequence of lithological layers, which are difficult to ascertain against the fieldwork notes ( Figure 13; Gennai, 2024).

- Humic level (h)
- Silty sandy brown-reddish loose sediment with gravel, 70 – 50 cm (Layer A)
- Greyish silty sandy sediment with gravel, increasing towards the bottom and progressively more compact 80 – 60 cm (Layer B). Pitti and Tozzi (1971) refined the knowledge of the main deposit (layer B), operating subdivisions according to the gravel content and progressive hardening of the sediment (from bottom to top B _3_, B _2_, B _1_).
- Fornaciari (1966) recorded a 25-cm-thick brown loamy layer with gravel, located in between the flowstone and the main deposit, which is not accounted for in Pitti and Tozzi (1971)
- Flowstone, 35 – 10 cm
- Yellowish loamy sediment, 50 – 30 cm
- Boulders

In 1968, samples of the flowstone were radiometrically dated with the Th ^230^/U ^230^ and gave two dates (< 41000 and < 51000 years), the discrepancy is due to the high content of clay in the flowstone ( Fornaca-Rinaldi & Radmilli, 1968). Because of the clay contamination and similarity with the nearby date of Grotta all’Onda, also obtained on a flowstone underneath sediment with Middle Palaeolithic artefacts, the younger date is favoured ( Pitti & Tozzi, 1971).

In the following years, the archaeological group lamented several damages to the remaining deposit, mainly due to looters. Therefore, to salvage the remaining deposits and archaeological evidence they excavated the northern profile in 1972 and 1973 ( Gennai, 2024). The new sectors were named A and B, progressing towards the North. In sector A the deposit was dug in 5 spits, all 30 cm thick except the initial spit 0, 23 cm thick, corresponding to the humic level. The deposit in A sloped towards the Buca della Iena niche (to the south) and consisted of mostly yellowish clay, becoming more reddish towards the bottom and with increasing stone content. A big boulder spanning the whole sequence was finally extracted in spit 4. In spit 4 a piece of flowstone was found too. Therefore, the excavators believed to have reached the end of the stratigraphical sequence. In sector B, four spits, each 30 cm thick, were dug. The sediment was much looser and consisted of reddish clay with loose gravel. The findings were similar to the previous excavations: Pleistocene fauna and some, dubious, Middle Palaeolithic artefacts.

Pitti and Tozzi (1971) reported one hundred and twenty-five (125) artefacts in total. Excluding eleven (11) artefacts out of context, they are almost equally subdivided into three groups (B _3_ = 46, B _2_ = 33, B _1_+A = 35). In the Archaeological Museum storehouse in Viareggio (LU), where the findings are stored and exposed, I found 157 artefacts, excluding some unmarked pieces of limestone ( Table 1). As the 1972 – 73 campaign did not produce a list of findings and sectors are named as the 1966 ones (A and B), it is impossible to ascertain how many artefacts have been added or if Pitti and Tozzi operated a selection. Pitti and Tozzi (1971) illustrated only artefacts typologically meaningful, I managed to recognise all the illustrated pieces in the current assemblage.

**Table 1.** Buca della Iena artefacts number.

Artefacts reported by Pitti and Tozzi (1971)	Artefacts found
125	157

### Grotta del Cervo

The site is adjacent to Buca della Iena, opening on the northern-facing side of the hill (43.9143446 N, 10.2850449 E; Figure 5; Gennai, 2024). The site is a bigger cavity than Buca della Iena, featuring two branches. It has never received an official name, being called like the nearby main village: Piano di Mommio. Informally, it is known as Grotta del Cervo ( Galiberti, 1997). The cave mouth faces West, the excavation was limited to the West by a drystone wall forming the modern terrace edge, to the South and the South-West by the Buca della Iena northern profile and a big limestone boulder, as to the North additional boulders were present.

**Figure 14.**
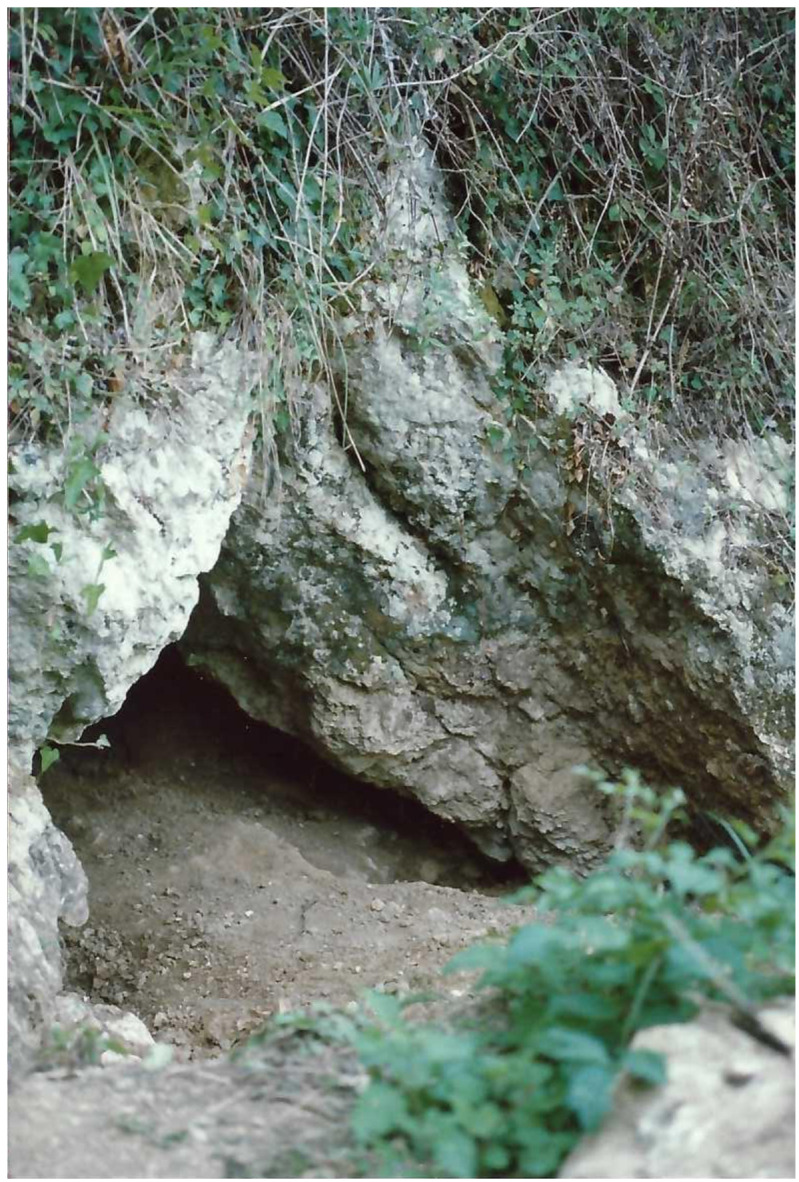
The Grotta del Cervo opening before excavation in 1988. Photo courtesy of Museo Civico and Archeologico e dell’Uomo “Carlo Alberto Blanc”.

The opening might have been discovered during the Buca della Iena excavations in 1972-1973, but systematic excavations started in 1989 ( Cocchi-Genick, 1989; Gennai, 2024). Before excavation, the opening was 3.85 m wide, 2.5 m deep and 0.80 m high ( Figure 14; Gennai, 2024). The excavation took place in four different campaigns in 1989 - 1992 and excavated the whole site, reaching 3.30 m of depth ( Cocchi-Genick, 1989; Cocchi-Genick, 1992; Cocchi-Genick, 1993). The site was divided into a 1 m ^2^ grid, with numbers from 1 to 7 progressing South to North on the X axis and letters from A to F progressing East to West on the Y axis.

This way, the deposit is roughly divided into two areas: the external deposit, corresponding to squares D1-F7, and the inner deposit, corresponding to squares C1-A7. The excavation proceeded by 5 cm spits. The soil was wet-sieved with a 2 mm mesh.

**Figure 15.**
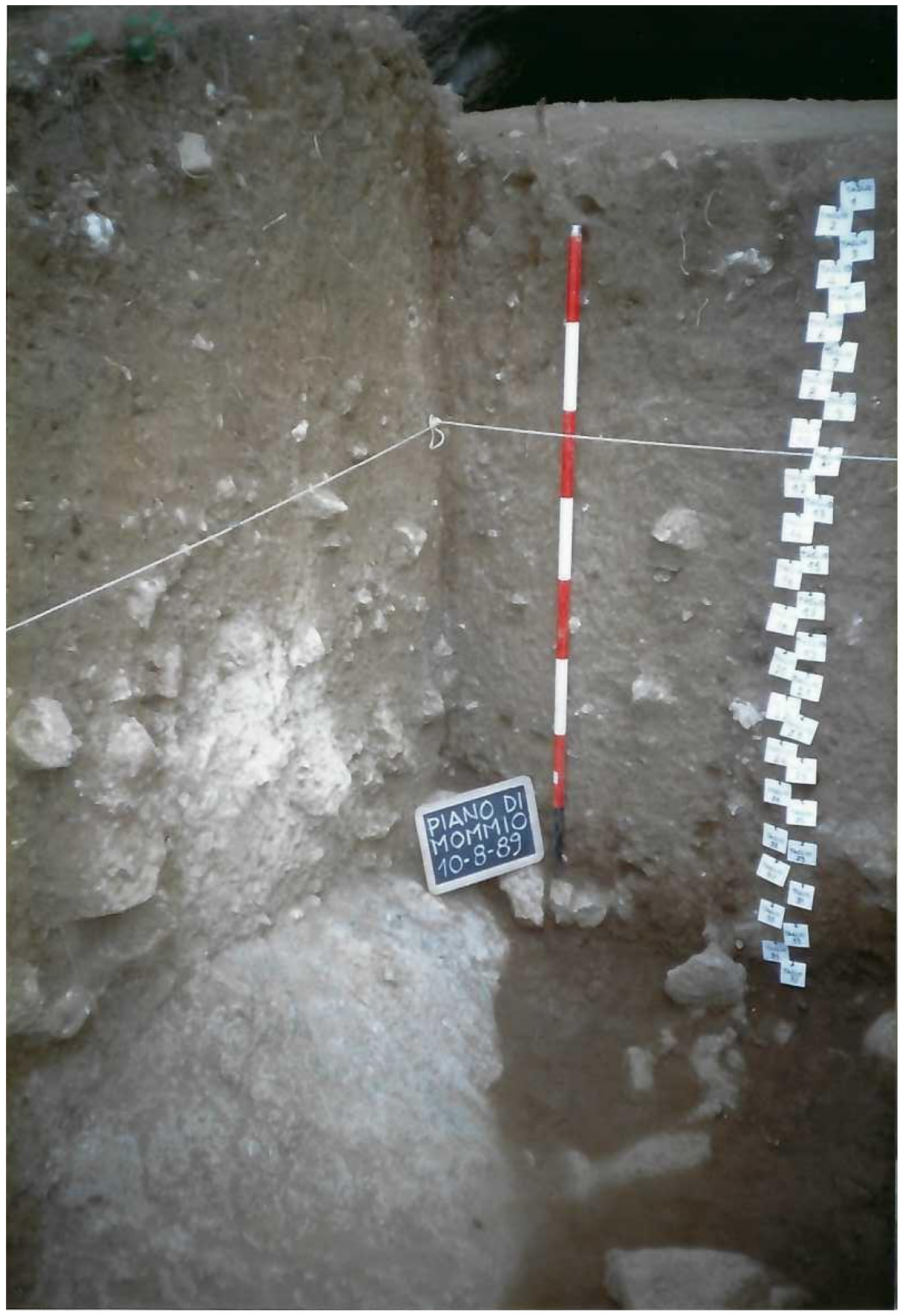
Grotta del Cervo complete excavated sequence, external area. Photo courtesy of Museo Civico and Archeologico e dell’Uomo “Carlo Alberto Blanc”.

During the first campaign, a 2x2 m trench was opened outside (F4-F5-E4-E5) and a second trench of 1x7 m opened a whole transect (D1-D7). The excavation of the external trench removed the humic level up to 30 cm depth and then reddish silt mixed with gravel up to one-metre depth from point 0. The same depth was reached in the inner cave and then excavation continued in the two branches, yielding human remains in B1 (-0.80 m and -1.80 m), A6 (-0.80 m) and B6 (-1.32 m). In C2 also a fragment of pottery was found (-1.30 m). The human remains, belonging to various juvenile and adult individuals, and the pottery fragment revealed a probable secondary burial that happened during the Copper Age, as attested by other nearby sites ( Fornaciari, 1977). The excavation stopped at 1.30 m depth in the northern branch arriving on top of a big stone layer and 2.20 m depth in the southern branch. Instead, in the external trench, it reached 2.90 m depth. The sediment is a homogenous reddish silt with gravel and bigger stones in the lower spits ( Figure 15; Gennai, 2024). Pleistocene fauna started appearing at 1.05 m depth with a higher frequency in the lower spits, Mousterian artefacts started appearing in spit 13 (1.65 – 1.70 m depth), with a higher frequency in the lower spits. The sediments appeared deeply reworked by fossorial animals and agricultural works, only at -2.80 m (spit 36) depth the sediments were hardened and preserved.

**Figure 16.**
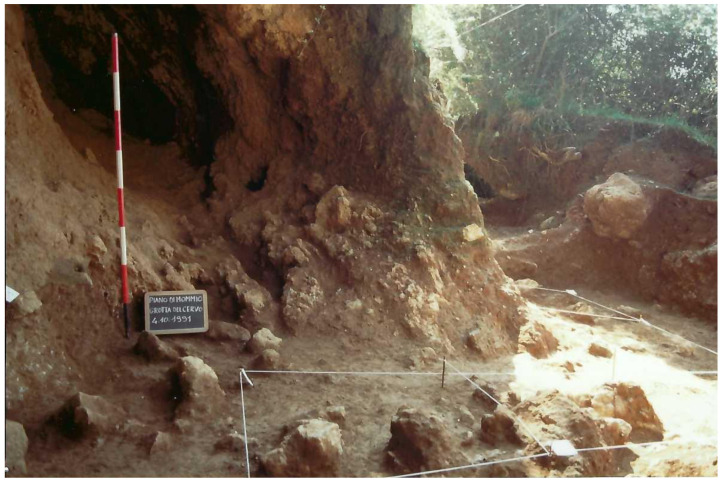
Grotta del Cervo southern branch, bottom of the sequence. The olive tree marks the border with the Buca della Iena excavation. Photo courtesy of Museo Civico and Archeologico e dell’Uomo “Carlo Alberto Blanc”.

During the second campaign, the southern branch of the cave was completely excavated until the stony level (2.40 m depth; Figure 16; Gennai, 2024). The rest of the external trench was enlarged, finding a similar deposit and with Pleistocene findings mostly coming from 2.40 m depth onwards.

The third campaign reached the 2.90 m depth, previously reached outside, inside the cave and expanded the external trench. Only sparse Mousterian lithic artefacts and Pleistocene fauna were found in the lower spits.

The fourth campaign reached the 3.30 m depth inside and externally, the sediment showing hardening and a higher frequency of Pleistocene fauna and some Mousterian lithic artefacts.

Daniela Cocchi-Genik reported eleven (11) lithic artefacts in the first excavation campaign, twelve (12) in the second one, and ten (10) in the last one ( Cocchi-Genick, 1989; Cocchi-Genick, 1992; Cocchi-Genick, 1993). In the Archaeological Museum storehouse in Viareggio (LU) I managed to find seventy (70) artefacts all labelled as belonging to the Grotta del Cervo site ( Table 2).

**Table 2.** Grotta del Cervo artefacts number.

Artefacts reported by Cocchi-Genick	Artefacts found
33	70

### Grotta del Capriolo

**Figure 17.**
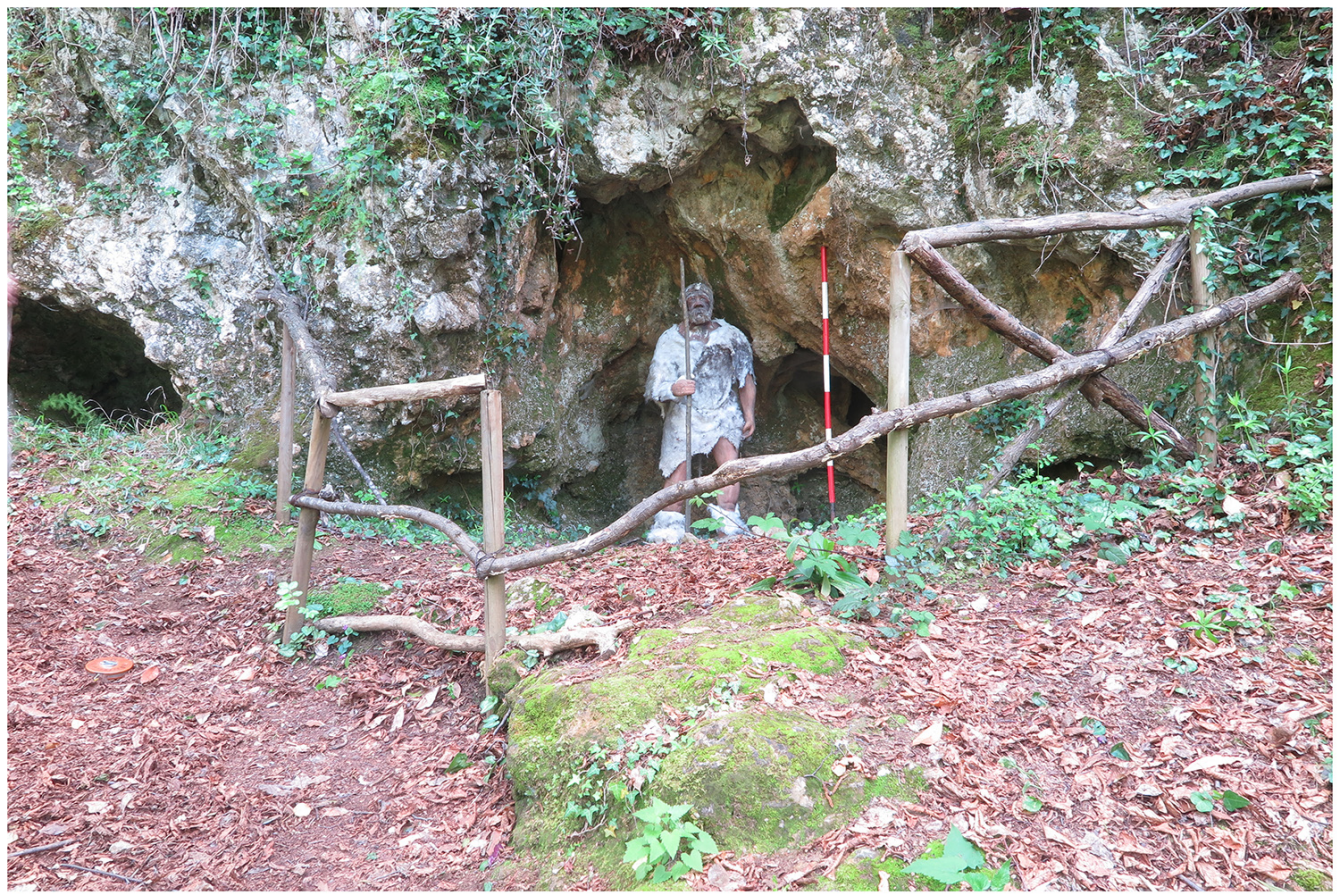
Grotta del Capriolo modern view. Photo Jacopo Gennai.

The Grotta del Capriolo is facing the Grotta del Cervo and Buca della Iena from the southern slope of the canyon at around 90 m a.s.l. (43.9136514 N, 10.2847033 E – Figure 5; Gennai, 2024). The site is a small cave opening in the same limestone outcrop as the above-mentioned sites, running continuously in the whole area. The opening faces to the North and the whole area is comprised of 20 m ^2^. The cave measures approximately 4.00 m in width, 3.00 m in length and 2.50 m in height ( Figure 17; Gennai, 2024).

**Figure 18.**
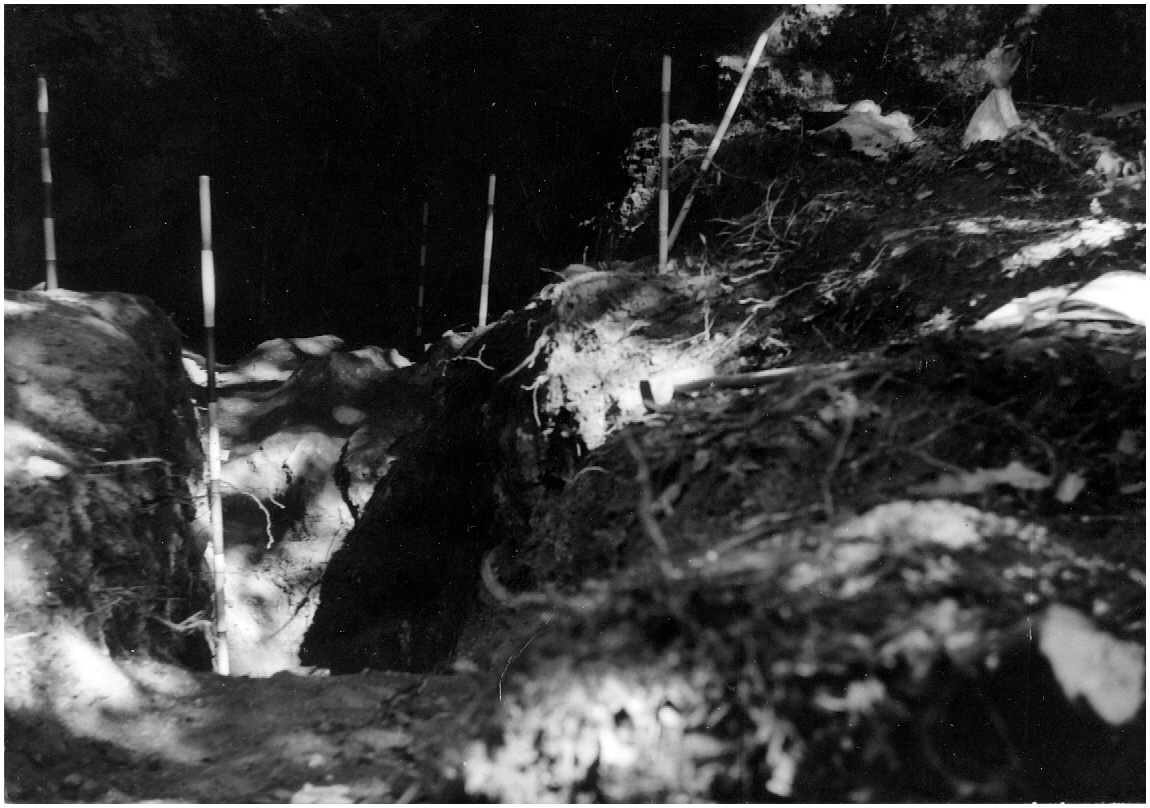
Grotta del Capriolo during excavation in 1968. Photo courtesy of Gino Fornaciari.

The site was discovered in February 1968 and found completely covered by the slope deposit ( Figure 18; Gennai, 2024). The site was excavated between the 24th of July and the 7th of August 1968 by the “Alberto Carlo Blanc” archaeological group ( Radmilli, 1968). Later in May-June 1970, the University of Pisa excavated the remaining deposit ( Pitti & Tozzi, 1971).

**Figure 19.**
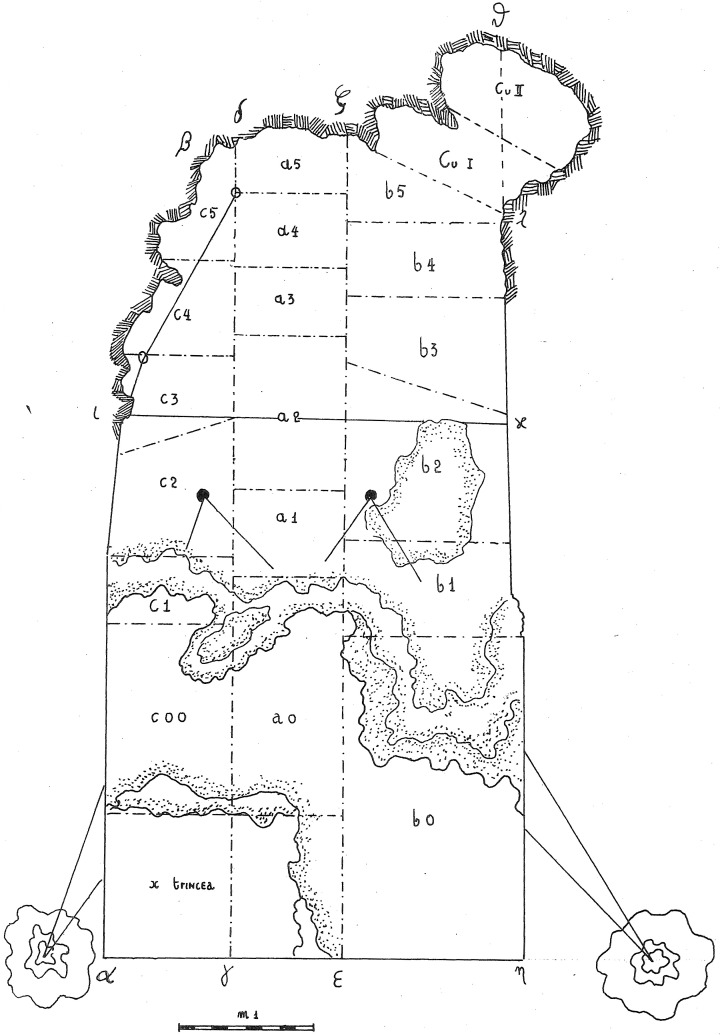
Grotta del Capriolo original site excavation plan 1968. Courtesy of Gino Fornaciari.

In 1968, the excavation of the site involved the creation of three trenches, each extending from the outermost to the innermost areas of the cave. Initially, they dug trench A, positioned at the central point, followed by trench B in the western sector, and finally trench C along the eastern cave wall. Trenches are divided into sectors, numbered sequentially from 0 to 5 as they progress from the exterior to the interior. Trenches and sectors do not follow a specific grid and they differ in extent ( Figure 19; Table 3; Gennai, 2024) Sector 0, yielded little archaeological sediment. Towards the back of the cave, two sectors, Cu I and Cu II, are covering a small niche.

**Table 3.** Grotta del Capriolo area of each sector and total area of excavation.

Sectors	B1	B2	B3	B4	B5	Cu I	Cu II	A1	A2	A3	A4	A5	C1	C2	C3	C4	C5	Tozzi trench	Tot
Area (m2)	0,92	1,64	1,07	0,63	0,78	0,57	0,51	0,53	0,94	0,41	0,45	0,36	0,47	0,91	0,38	0,48	0,33	1,7	13,07

**Figure 20.**
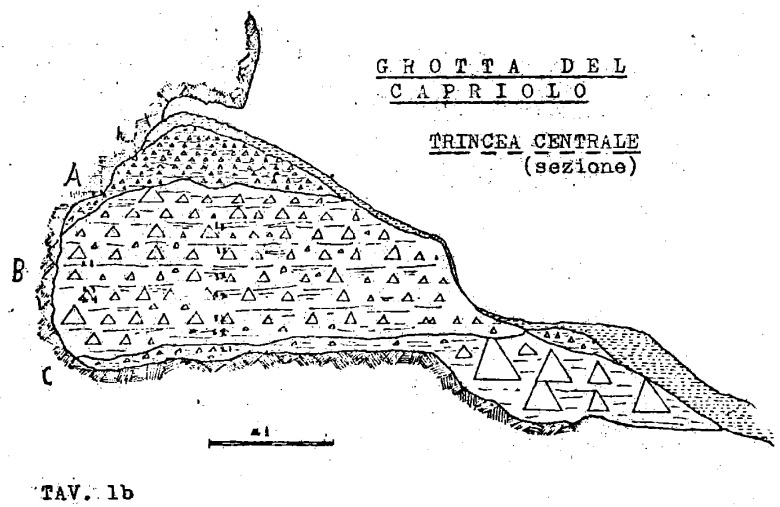
Grotta del Capriolo original Trench A profile section drawing.

**Figure 21.**
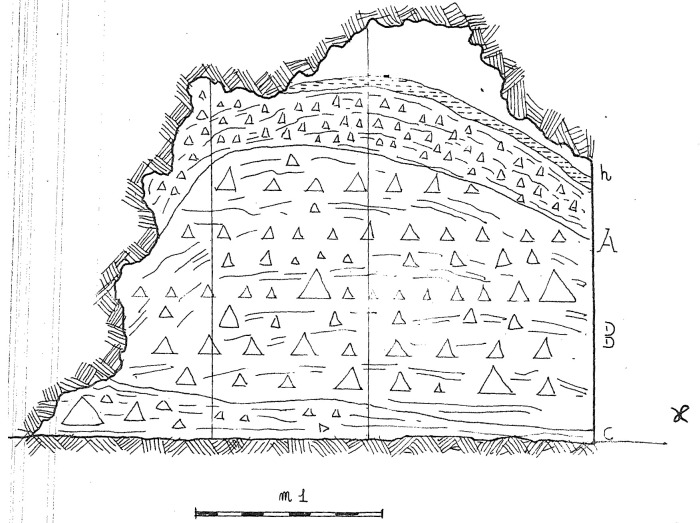
Grotta del Capriolo original transversal (C3-A3-B2/B3) profile section drawing. Courtesy of Gino Fornaciari.

**Figure 22.**
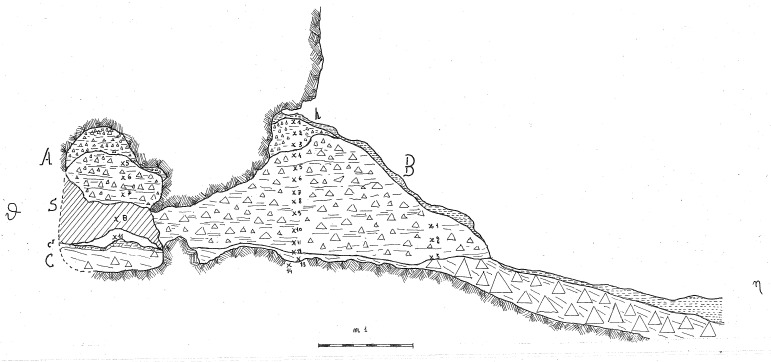
Grotta del Capriolo original Trench B profile section drawing. Courtesy of Gino Fornaciari.

The deposit was excavated in spits, with the total number of spits varying based on factors such as the specific sector, deposit thickness, and the inherent thickness of the spits themselves. For instance, trench A, at its highest point, comprised 7 spits ( Figure 20; Figure 21; Gennai, 2024), while trench B featured 14 spits ( Figure 22; Gennai, 2024), and trench C had 11 spits. The thickness of these spits fluctuated, ranging from 30–40 cm down to 10 cm. Dry sieving was conducted on-site, enabling the recovery of artefacts as small as 10 mm in their maximum dimension.

In 1970, excavation focused on the remaining sediment, which was designated as trench DE ( Gennai, 2024). Unfortunately, only a few fieldwork notes have survived from this period. The deposit was excavated in arbitrary spits, beginning with spit A9 and continuing up to A15. Other spits were denoted with the letters D, E1, E2, and F. The starting excavation height was 50 cm from a reference point, although the exact reference point height remains undocumented. Nevertheless, spatial coordinates (x, y, z) are recorded for some of the artefacts.

The excavations reported a similar stratigraphy.

- Superficial humic level, 5 – 20 cm (h)
- brown-reddish silt with little gravel and devoid of any archaeological material, 20 – 50 cm (A)
- light brown silt with progressively increasing gravel bearing archaeological Mousterian artefacts and Pleistocene fauna, 60/95 – 130 cm (B, subdivisions from bottom to top B _3_, B _2_, B _1_)
- yellow silt covering clast-supported sediment and big boulders interpreted as the bottom of the sequence, 5 – 20 cm (C or D)

The 1968 excavations also recognised a flowstone in Cu I and Cu II. A much more cemented area of sediment is found in the back of the cave (S). The 1970 excavation subdivided layer B into three horizons following the progressive increase of gravel and hardening of the sediments ( Pitti & Tozzi, 1971).

Pitti and Tozzi (1971) reported five hundred and forty-one (541) artefacts in total. Excluding sixty-one (61) artefacts out of context, they are almost equally subdivided into three groups (B _3_ = 117, B _2_ = 200, B _1_ = 163). In the Archaeological Museum storehouse in Viareggio (LU), where the findings are stored and exposed, I found 539 artefacts, excluding some unmarked pieces of limestone. According to the artefact list from the 1968 and 1970 campaigns, 367 artefacts were found in 1968 and 88 were found in 1970 ( Table 4). Many artefacts from 1968 were likely not considered before publication. Most of the artefacts found in 1970 are out of context as they lay on the surface or were part of disturbed sediment. Pitti and Tozzi (1971) illustrated only artefacts typologically meaningful, I managed to recognise all the illustrated pieces in the current assemblage.

**Table 4.** Grotta del Capriolo artefacts number.

Artefacts reported by Pitti and Tozzi (1971)	1968 artefacts recorded on the list	1970 artefacts recorded on the list	Artefacts found
541	367	88	539

## Discussion: new stratigraphical interpretation

The different degrees of documentation survival for the three sites account for the degree of stratigraphical reconstruction and new interpretation. In terms of the sheer number of artefacts, the reassessment changed the available counts.

### Buca della Iena

The absence of quotes taken from a conventional point zero and the reporting of average thicknesses allow only a rough estimate of the extent and position of the lithological layers. Also, the position of the spits on the profile section cannot be translated into precise measurements. Therefore, only a reconstruction of the spits’ relative positions within the stratigraphy and their correlations, reported in the fieldwork notes, is here proposed. The main achieved results are:

- Recognising that vertical spans reported in the published sequence are probably averages, which cannot be reproduced using field notes.
- Attempting a reconstruction of sectors’ relative positions on a bi-dimensional plan, as an excavation plan is unavailable and not mentioned in the published sources.
- Recognising that the stratigraphical integrity of the sequence varies according to the excavation sector.
- Spits correlation is mostly done according to sedimentary composition.

**Figure 23.**
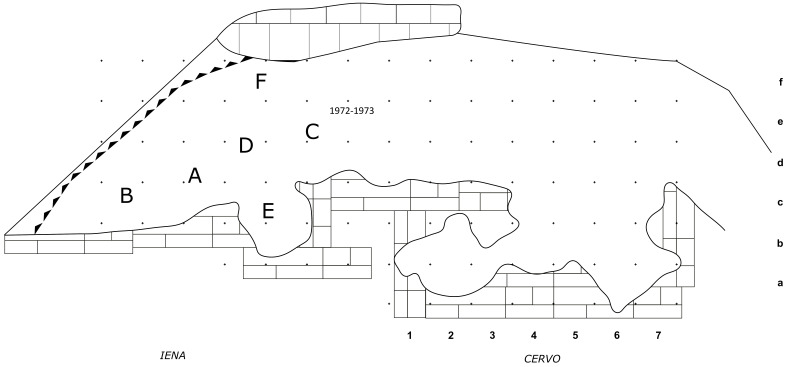
Buca della Iena and Grotta del Cervo redrawn site plan. Drawing Jacopo Gennai.

**Figure 24.**
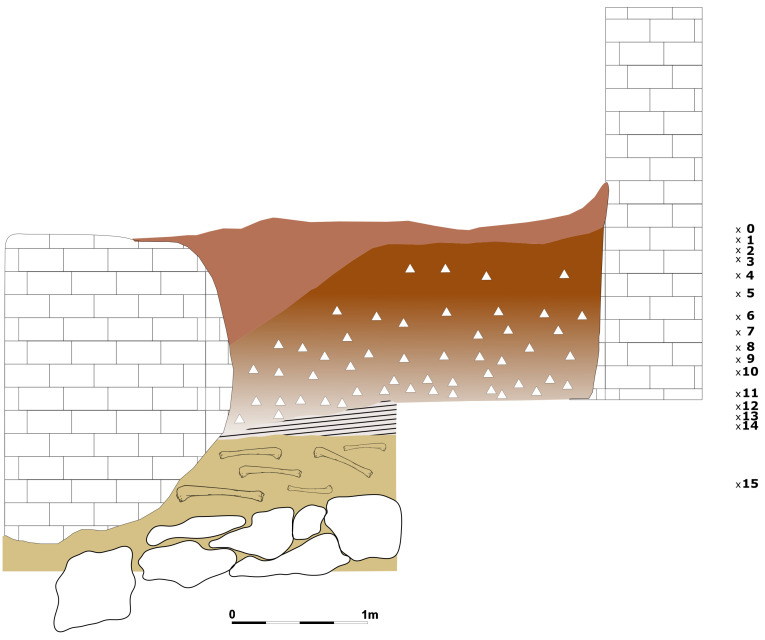
Buca della Iena northern redrawn profile section. Drawing Jacopo Gennai.

Nowadays the current extent of the terrace accounts for a roughly 25 m ^2^ area ( Figure 23; Gennai, 2024). Reconstruction of excavation sectors goes as follows: to the north sectors A and B (1972–73) and sector C run across the width of the terrace from the limestone wall to the big boulder. Attempting to reconstruct stratigraphical relationships between this area and the rest of the Buca della Iena site is tentative at best. While the sedimentary content in A (1972–73) seems more similar to that found at the bottom of the Buca della Iena deposit, in B (1972–73) it seems to correspond to the topmost part. This might lead to the speculation that the B sector is mostly next to the limestone cliff, while the A sector is in the front. More to the south, in front of the opening of the niche, supposedly lays sector D and in front sector F, representing the edge of the terrace with sediments and the flowstone sloping downwards. Sectors C and D were the most intact and the highest of the whole deposit ( Figure 24; Gennai, 2024). Their sediments sloped frontally towards F, which showed a shift of the same sediments one spit lower than D and C. Sector C and D sediments covered the opening of the niche which corresponded to the sector E. Sector E sediments probably sloped inside and compacted, also the weathering of the wall accumulated more clastic sediments towards the cave end. To the South, sector A was probably sloping laterally and frontally because the deposit was only one metre high and excavated in four spits. B was probably in the front, on the slope, because only two spits are mentioned and sedimentologically the B – 2 is correlated with F – 14/15. The yellow clay is found only in these two lowermost spits ( Table 5; Gennai, 2024). According to this reconstruction, Sectors A and B are highly unreliable for stratigraphical purposes as they show a low degree of stratigraphical resolution. Spit 1 of Sector A is correlated with spits 7 and 8 of Sectors C and D, but it contains reddish clay sediment (found in spits 2 to 4 in Sectors C and D). Therefore, the deposit in Sectors A and B is probably a result of erosion from C and D. Only the lower part, yellow clay, might be in place. Sector E is likely affected by stratigraphical sequence compaction too, which is usual for areas next to the cave walls. This is shown in the final picture of the excavation ( Figure 13 – Gennai, 2024) as the hardened flowstone is rising upwards toward the limestone cliff. Sector F's upper part is probably showing sloping of sediments from C and D. As shown from the picture of the northern profile (spanning Sectors C and F – Figure 13; Gennai, 2024) spits 5 to 11 might be considered sub-horizontal and unaffected by disturbances. In the front of the deposit, sediment is slightly sloping also according to the thinner flowstone. The flowstone is thinning towards the end of the slope and in Sector A. The reassessment found more artefacts than the published ones. Even if some might belong to 1972 - 1973 excavations, it does not seem this campaign found thirty-two (32) more artefacts. Some artefacts might have been left out from Pitti and Tozzi's (1971) analysis.

**Table 5.** Buca della Iena Correlation of spits in the different sectors.

B	A	C	D	E	F
		1	1		
		2	2		
		3	3		
		4	4		
		5	5	5	
		6	6	6	
	1	7	7	7	7
		8	8	X	8
	2	9	9		9
	3	10	10		10
	4	11	11	11	11
		12	12	12	12
1				13	13
			13	14	
2					14
					15

### Grotta del Cervo

The main result of Grotta del Cervo's discussion is the correlation with the neighbouring Buca della Iena. No correlations are mentioned in the sources, only a fleeting comment about the Buca della Iena northern profile providing a limit for the Grotta del Cervo excavation. Despite being the two sites next to each other, no comprehensive site plan was drawn. Here, a comprehensive position of the two sites has been reported ( Figure 23; Gennai, 2024).

**Figure 25.**
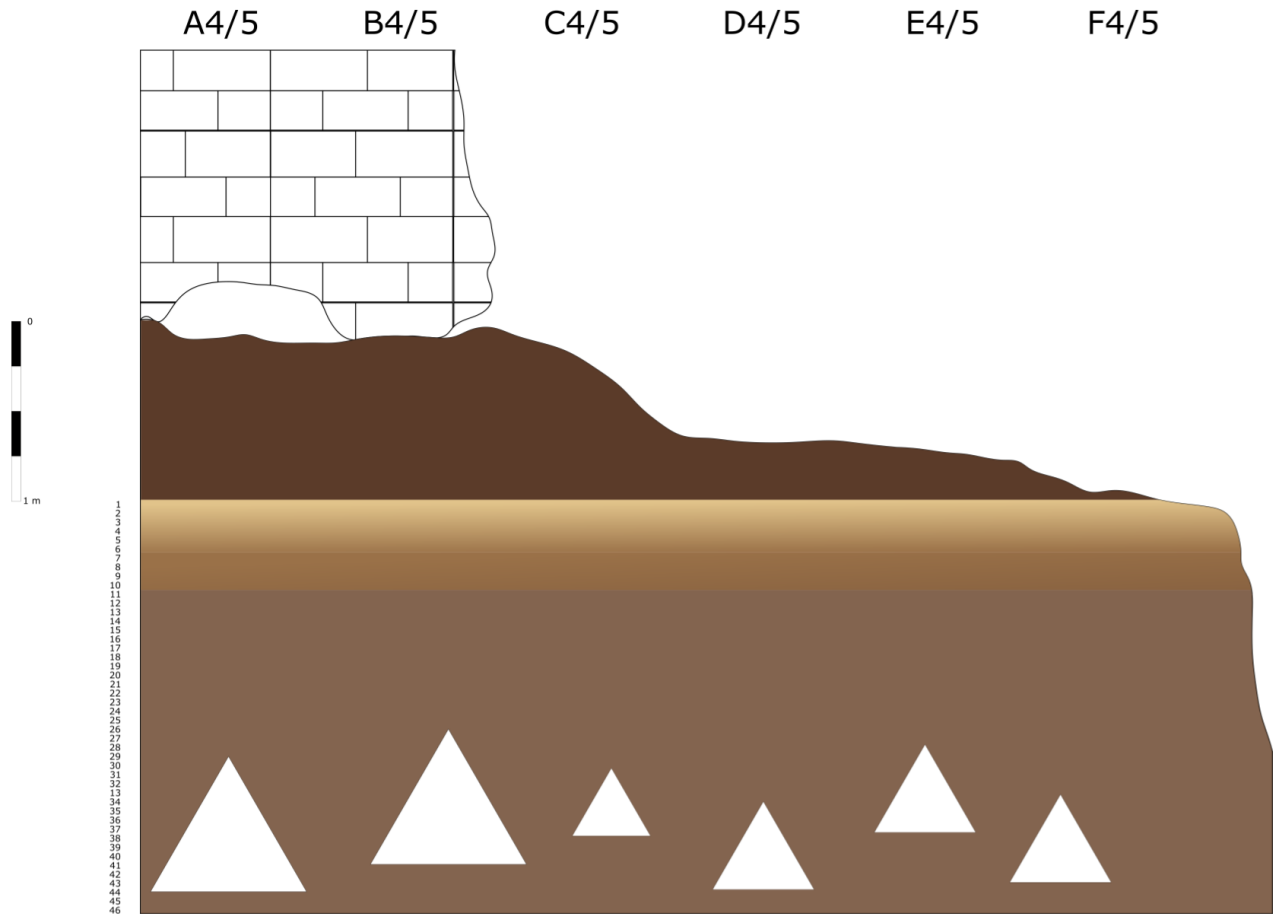
Grotta del Cervo profile section redrawn. Drawing Jacopo Gennai.

Grotta del Cervo shows many analogies with the adjacent Buca della Iena. The sediment is a brownish-reddish loose silt with gravel progressively hardening with depth ( Figure 25; Gennai, 2024). Also, the increase of stones and boulders in the lower spits is similar to the stratigraphical sequence in Buca della Iena. Tentative correlations might be operated between spit 5 - 6 of Buca della Iena and spit 1 of Grotta del Cervo (1.05 – 1.10 m depth). Since no sloping is reported by the excavators, it is assumed that similarly numbered spits reached the same height in each part of the excavation, forming a subhorizontal surface. Buca della Iena and the adjacent Grotta del Cervo might have been part of the same site frequented by Middle Palaeolithic groups. Unfortunately, as the two excavations have been refilled correlation between the two sites is tentative. A correlation between Buca della Iena 1972 – 73 sectors and Grotta del Cervo seems unlikely as the reported sedimentary contents differ. The reassessment almost doubled the artefacts found at the site.

### Grotta del Capriolo

**Figure 26.**
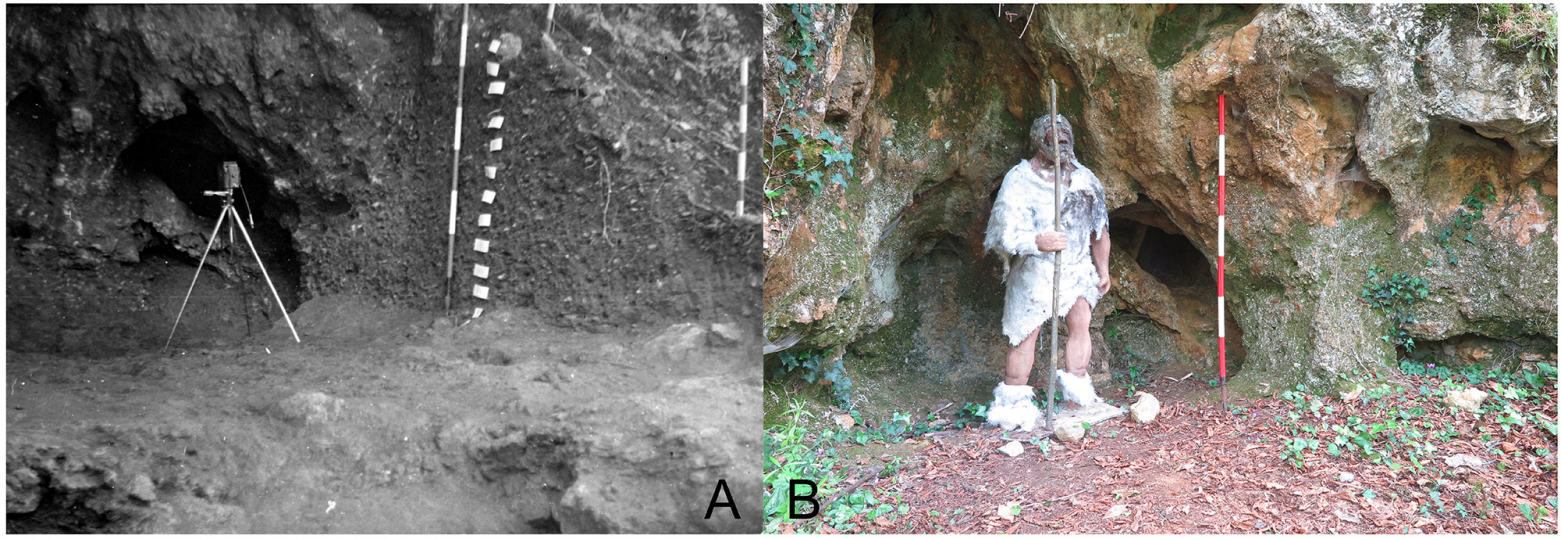
Grotta del Capriolo western cave wall. Comparison between the end of 1968 Grotta del Capriolo excavation ( **a**) and modern state ( **b**). Photo Jacopo Gennai and Gino Fornaciari.

**Figure 27.**
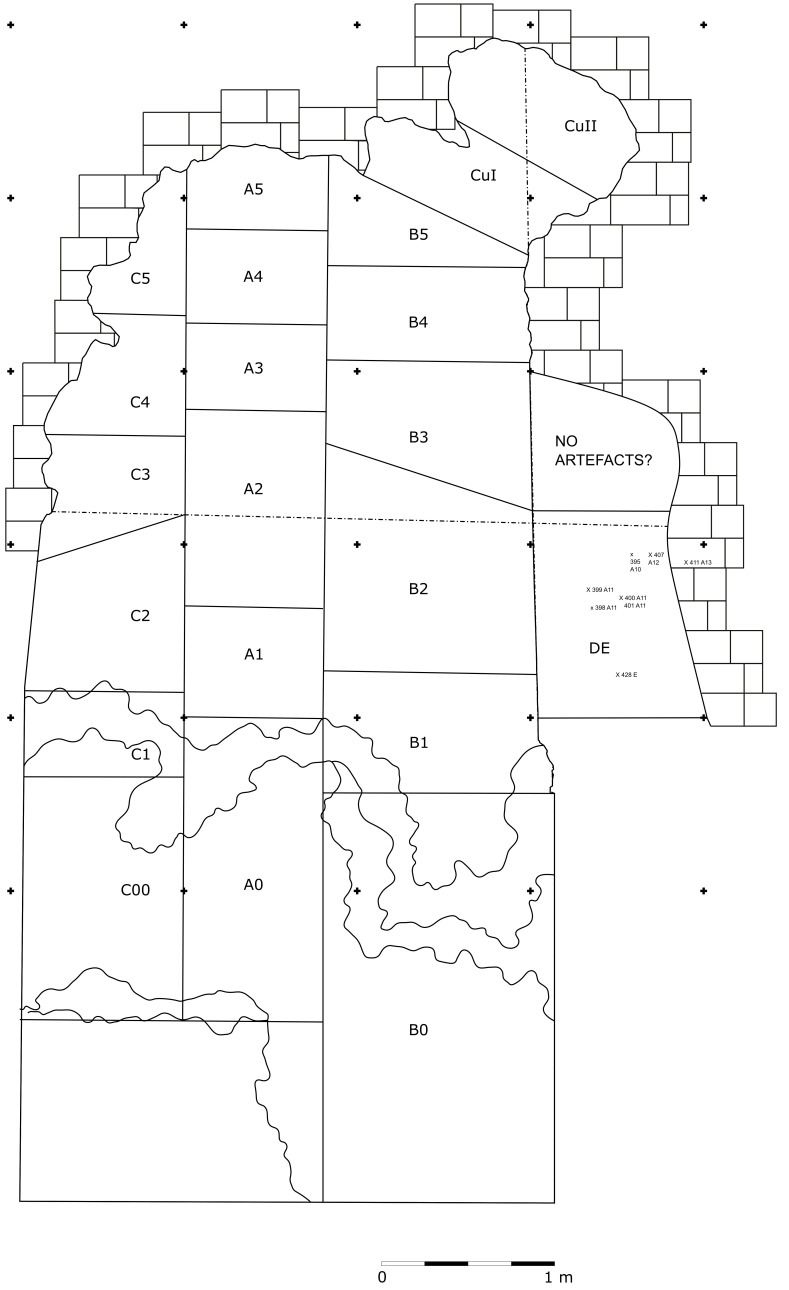
Grotta del Capriolo redrawn site plan including trench DE. Drawing Jacopo Gennai.

**Figure 28.**
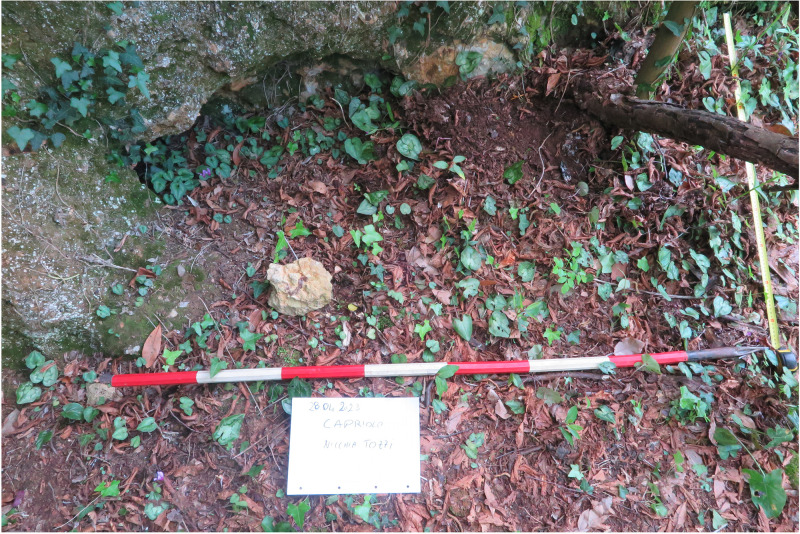
The likely location of trench DE. Photo Jacopo Gennai.

**Figure 29.**
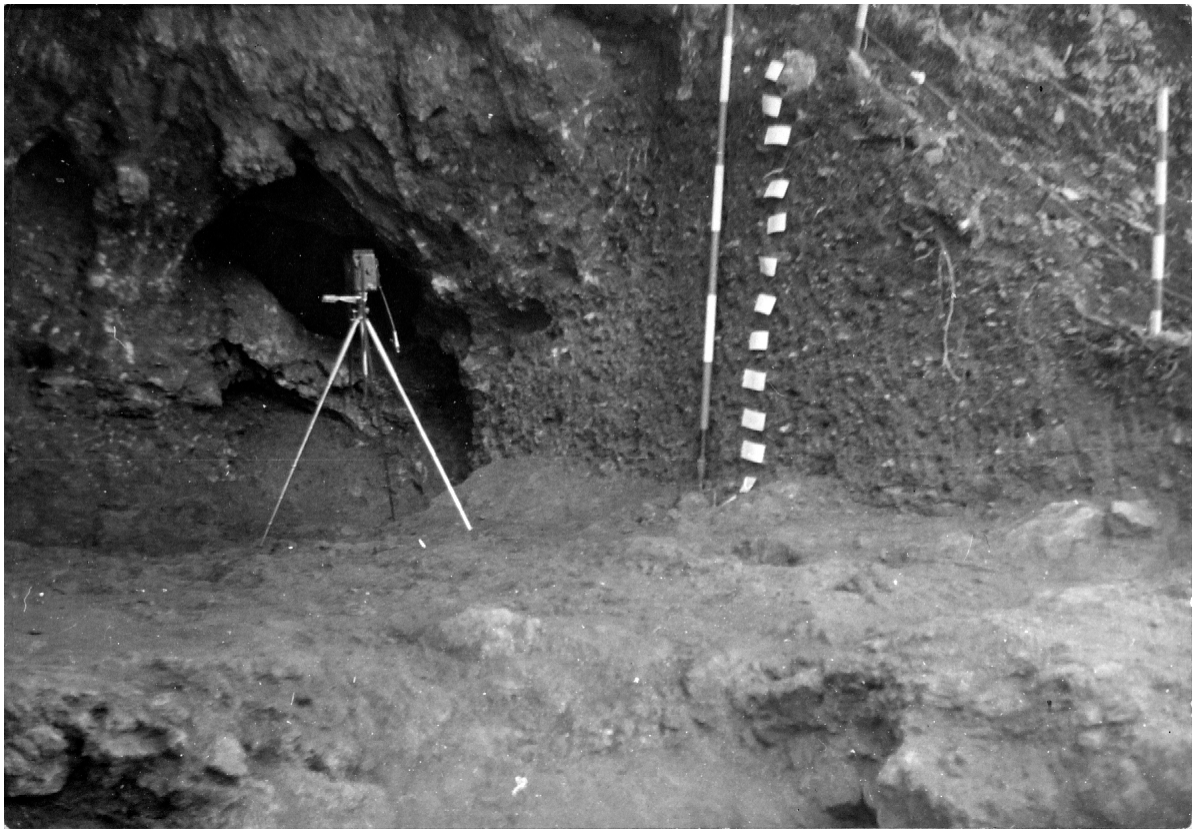
The end of 1968 Grotta del Capriolo excavation and Trench B profile section. Photo Gino Fornaciari.

**Figure 30.**
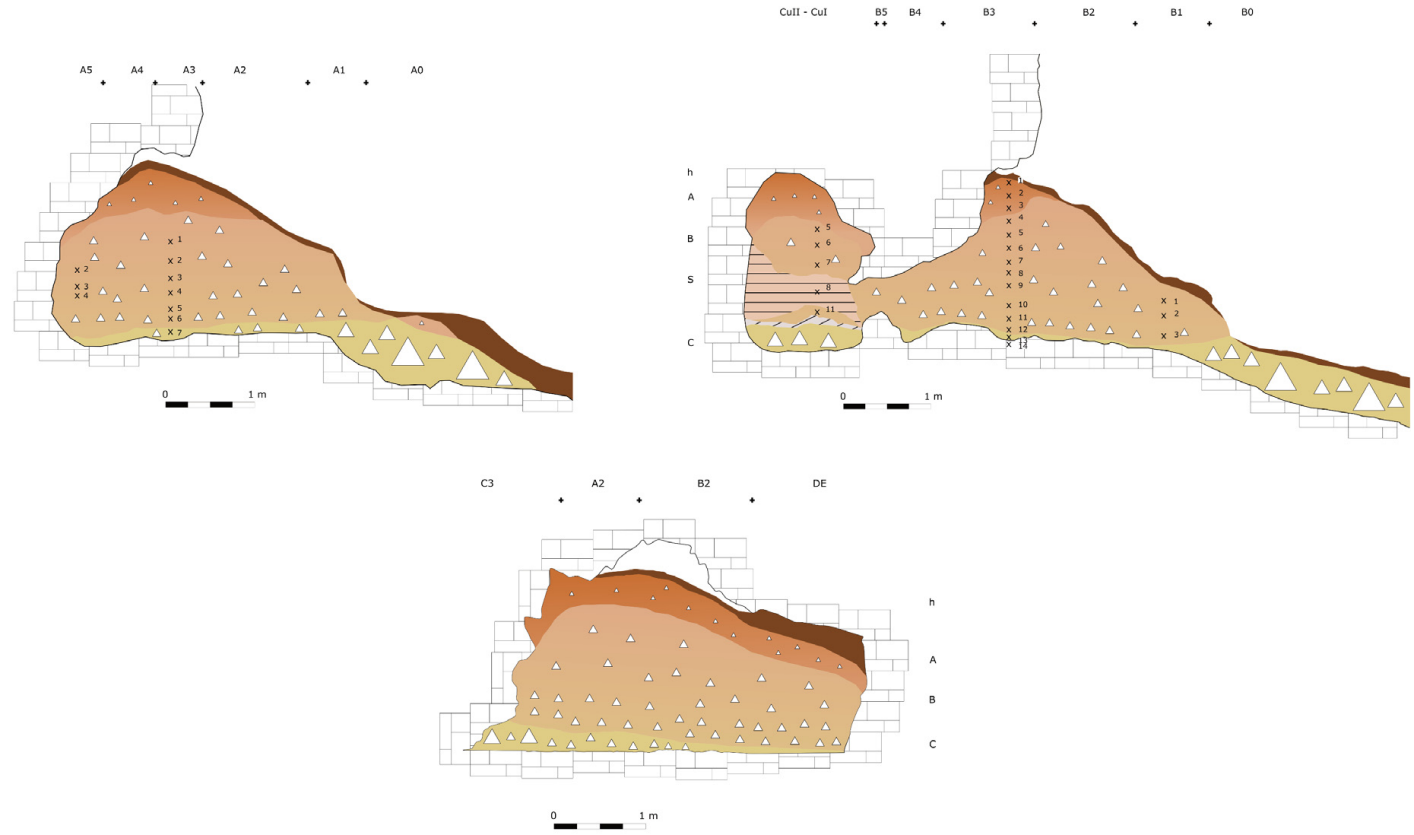
Grotta del Capriolo 1968 redrawn profile sections drawings. Drawing Jacopo Gennai.

In contrast to the relatively comprehensive documentation available for Buca della Iena, the records for Grotta del Capriolo are rather sparse. The excavation diaries are either missing or contain minimal information. However, we do have the benefit of surviving plans and three section profiles’ drawings that describe the 1968 excavation. Additionally, a few photographs from the 1968 excavation have survived. As it is, the Grotta del Capriolo sequence is the most re-interpreted as no notes from the original excavators are available. Both the photographic and graphical documentation align well with the present-day conditions of the site ( Figure 26; Gennai, 2024). Unfortunately, pinpointing the precise extent and location of trench DE remains a challenge. Nevertheless thanks to the modern survey and the newly available 1968 end of excavation picture it is possible to confine Trench DE to the small area between 1968 Trench B and the external western wall ( Figure 27; Figure 28; Gennai, 2024). As depicted in the photographs and section profiles ( Figure 17; Figure 29; Figure 30; Gennai, 2024), the distribution of the deposit followed the primary hill slope. Consequently, the highest accumulation of sediment can be observed in sectors A2 and A3, gradually sloping towards the cave walls, particularly evident in Trench B. Simultaneously, the deposit conformed to the slope of the main hill, resulting in less sediment in sectors 1, with the highest accumulation occurring in sectors 2 and 3, which are positioned adjacent to the modern dripline. The approximate positions of spits are recorded on the trench A and B profile sections. Consequently, the correlations of spits based on the topography of the deposit could estimated: this led to the grouping of spits of different sectors in seven (7) levels ( Table 6). Nevertheless, sub-horizontality of the levels cannot be assumed as the sediment probably accumulated as a cone, peaking around the middle of the sites and the modern dripline, and sloping in all directions. The apparent homogeneity of the sediments does not help in recognising discrete levels within the main artefact-bearing deposit (former Layer B). Therefore, only the central area (Sectors A2/3, partially B2/3) might be considered as preserving some stratigraphical integrity. Artefacts and sediments in the front, back and sides of the deposit might be considered mixed by slope processes. Understanding the spatial organization of the DE trench and its spits is a complex undertaking. The initial excavation height was set at 50 cm, which aligns well with the beginning of Layer B next to the cave's western wall and the highest points where artefacts were buried (z). According to notes accompanying the artefacts' list, spits E and F correspond to the lower portion of the deposit and are sedimentologically similar to the lowest Layer B and to Layer C. Specifically, spits A9 to A15 cover the upper section (z= 47 – 102 cm), while spits F and E encompass the lower section (z= 80 – 125/138 cm). By examining the x and y coordinates of artefacts in spits A and E, the approximate position can be deduced. Spits A9 – 15 and D are situated near sector B2 and can be correlated with spits B2-6 to B2-9 ( Figure 29; Figure 30; Gennai, 2024). Conversely, spits E are located adjacent to sector B1 and can be correlated with spits B1-2 and 3 ( Table 6). As Trench DE is essentially an appendix of Trench B extending up to the western wall, the positions of the artefacts are to be considered affected by slope processes. The reassessment found almost the same number of published artefacts. The main difference is the lower number of artefacts which is reported in the 1968 list.

**Table 6.** Grotta del Capriolo spits’ correlation.

C1	A1	B1	C2/C3/C4	A2/A3/A4	B2/B3/B4	A5	B5	C5	DE	New Level
			1	1	1	1	5			Level 1
					2					
					3					
			2		4					
					5					
							6			
			3	2	6	1	7		A9	Level 2
			4		7				A10	
								A11		
1	1		5	3	8	2	8		A12	Level 3
2	2	1	6	4	9	3-4			A13	Level 4
									A14	
									A15	
3	3	2	7	5	10	5	9		E1	Level 5
4			8			10				
						11				
5		3	9	6	11		12	8-9	E2	Level 6
6			10	7	12	7	13	10	F	Level 7
7			11		13		14	11		
					14					

## Conclusions

The collaborative efforts invested in digitizing, preserving, and disseminating previously unexplored documentation have significantly advanced the accessibility and transparency within the field of archaeological research. This transparency helps in cultivating an environment of cooperation and knowledge exchange, ultimately proving advantageous for the broader scientific community. The manuscript delved into the intricacies of reconstructing stratigraphical relationships of long-gone deposits in three sites of north-western Tuscany (Italy): Buca della Iena, Grotta del Capriolo and Grotta del Cervo. The sites are important as they feature Mousterian artefacts, probably belonging to the Middle to Upper Palaeolithic Transition (MUPT) period, and they fill a geographical gap of MUPT sites presence between North-Western Liguria and Southern Tuscany. The rarity of stratified contexts in the area makes the reassessment pivotal to provide a baseline for future interpretations and research in the area. This is most important, as the other sites in the area (Grotta all’Onda and Buca del Tasso) feature a more ephemeral human occupation than Buca della Iena, Grotta del Capriolo and Grotta del Cervo.

 Buca della Iena and Grotta del Capriolo's stratigraphical context is barely discussed within the original publication and stratigraphical information marked on the findings cannot be related to the published sequence. Using previously unpublished documentation from these sites has played a pivotal role in addressing this lacuna in our understanding of the sites’ artefact burial context. The incorporation of previously undisclosed field notes, diaries, photographs, and drawings provides insights into the intricacies of the excavation procedures, enabling a more precise and nuanced reconstruction of the stratigraphical sequences at these sites. Thanks to this effort, the stratigraphical reliability of the findings is accessible. Also, the manuscript provides all information in English allowing access to international researchers.

Regarding Buca della Iena, this reassessment has resulted in the systematic identification of sectors and the subsequent correlation between various spits. The reassessment provides a safer reliability for findings located in Sectors C, D, and lower Sector F. The most noteworthy outcomes, however, have originated from the re-evaluation of Grotta del Capriolo, where an understanding of the precise positioning of spits and their associated heights would have remained elusive without access to the original section profiles. Grotta del Capriolo deposit must be considered cone-shaped, therefore any findings away from the cone centre are likely affected by slope processes. The manuscript reviews existing information from Grotta del Cervo and tries to establish correlations with the neighbouring Buca della Iena.

Following the re-evaluation of the three sites’ stratigraphy, ongoing investigations are focusing on the technological attributes of each archaeological assemblage and gaining insights into the site functions. Furthermore, radiocarbon dating has been initiated to refine our chronostratigraphic understanding of the sequences. In summary, the assimilation of previously unpublished documentation will provide the necessary backbone to introduce fresh perspectives on Neanderthal presence in Western Italy and enhance our understanding of the MUPT.

## Ethics and consent

Ethical approval and consent were not required.

## Data Availability

DARIAH-DE: Underlying data for ‘The Mousterian in North-Western Tuscany: publishing fieldwork documentation leads to a new stratigraphical interpretation from of the Piano di Mommio sites ‘ The Tuscan Mousterian, https://doi.org/10.20375/0000-0011-BF54-8 ( Gennai, 2024). This project contains the following underlying data: Figure 1_Gennai_Toscana_Musteriano.tiff (image/tiff) Figure 2_Gennai_Piano di Mommio sites and geology.tiff (image/tiff) Figure 3_Gennai_Mousterian artefacts.tiff (image/tiff) Figure 4_Gennai_Buca della Iena opening.tiff (image/tiff) Figure 5_Gennai_Buca della Iena terrace.tiff (image/tiff) Figure 6_Gennai_Buca della Iena northern profile section drawing 1966.tiff (image/tiff) Figure 7_Gennai_Buca della Iena before excavation in 1966.tiff (image/tiff) Figure 8_Gennai_Buca della Iena bottom of the sequence 1966.tiff (image/tiff) Figure 9_Gennai_Buca della Iena Northern profile after excavation in 1966.tiff (image/tiff) Figure 10_Gennai_Grotta del Cervo before excavation 1988.tiff (image/tiff) Figure 11_Gennai_Grotta del Cervo external area complete excavated sequence.tiff (image/tiff) Figure 12_Gennai_Grotta del Cervo bottom of the sequence inner southern area.tiff (image/tiff) Figure 13_Gennai_Grotta del Capriolo modern view.tiff (image/tiff) Figure 14_Gennai_Grotta del Capriolo during excavation 1968 trench A.tiff (image/tiff) Figure 15_Gennai_Grotta del Capriolo site plan.tiff (image/tiff) Figure 16_Gennai_Grotta del Capriolo transversal section drawing 1968.tiff (image/tiff) Figure 17_Gennai_Grotta del Capriolo trench A profile section drawing.tiff (image/tiff) Figure 18_Gennai_Grotta del Capriolo trench B profile section drawing.tiff (image/tiff) Figure 19_ Gennai_Buca della Iena and Grotta del Cervo redrawn site plan.tiff (image/tiff) Figure 20_Gennai_Buca della Iena redrawn Northern profile sectiion.tiff (image/tiff) Figure 21_Gennai_Grotta del Cervo profile section.tiff (image/tiff) Figure 22_Gennai_Grotta del Capriolo comparison between end of excavation 1968 and modern.tiff (image/tiff) Figure 23_Gennai_Grotta del Capriolo redrawn comprehensive site plan.tiff (image/tiff) Figure 25_Gennai_Grotta del Capriolo end of excavation 1968.tiff (image/tiff) Figure 24_modern niche DE.tiff (image/tiff) Figure 26_Gennai_Grotta del Capriolo sections redrawn.tiff (image/tiff) SI_Gennai_Piano di Mommio.pdf (Text/pdf) bollettino gruppo di ricerche archeologiche Blanc_1_1967.pdf (application/pdf) bollettino gruppo di ricerche archeologiche Blanc_5_1968.pdf (Text/pdf) bollettino gruppo di ricerche archeologiche Blanc_9_1972.pdf (Text/pdf) bollettino gruppo di ricerche archeologiche Blanc_11_1973 .pdf (Text/pdf) bollettino gruppo di ricerche archeologiche Blanc_13_1975.pdf (Text/pdf) Buca della Iena 1966-1973 settori A e B.pdf (Text/pdf) Grotta del Capriolo_diario di scavo Tozzi 1970.pdf (Text/pdf) Grotta del Capriolo_inventario manufatti Fornaciari-Tozzi.pdf (Text/pdf) Grotta del Cervo_1989_Cocchi.pdf (Text/pdf) Buca Iena_Diario Fornaciari trascritto.pdf (Text/pdf) Siti Musteriano Toscana.csv (Dataset/csv) Siti Musteriano Toscana.xlsx (Datasets/xlsx) The Tuscan Mousterian 2. https://doi.org/10.20375/0000-0011-de19-8 ( Gennai, 2024a) This project contains the following underlying data: Grotta all'Onda.tiff (image/tiff) Buca del Tasso.tiff (image/tiff) Tecchia e Riparo Equi_unito.tiff (image/tiff) Buca della Iena_end of the modern terrace_boulder.tiff (image/tiff) Data are available under the terms of the Creative Commons Attribution 4.0 International license (CC-BY 4.0)
