# Supplementary material for: The Mousterian in North-Western Tuscany: publishing fieldwork documentation leads to a new stratigraphical interpretation of the Piano di Mommio sites
Source: Open Res Eur. 2024 Jun 21;4:37. Originally published 2024 Feb 22. [Version 2] doi: 10.12688/openreseurope.17018.2 (PMC11109565; doi:10.12688/openreseurope.17018.2)
Supplement: Peer review history

# Reviewer report: Reviewer response for version 2

**DOI:** 10.21956/openreseurope.19288.r41571

**Published:** 2024-07-12

**Authors:** Kévin Di Modica

**Affiliations:** 1 Scladina Cave Archaeological Centre, Espace Muséal d’Andenne, Andenne, Belgium

The author took into consideration our comments on Version 1. I am ok with the changes.

If any, are all the source data and materials underlying the results available?

Yes

Is the work original in terms of material and argument?

Partly

Is the work clearly and cogently presented?

Yes

Is the argument persuasive and supported by evidence?

Partly

Does the research article contribute to the cultural, historical, social understanding of the field?

Yes

Does it sufficiently engage with relevant methodologies and secondary literature on the topic?

Partly

Reviewer Expertise:

Middle Palaeolithic in NW Europe; Site stratigraphy; Old collections reappraisal; Lithic studies; Chronostratigraphy

I confirm that I have read this submission and believe that I have an appropriate level of expertise to confirm that it is of an acceptable scientific standard.

# Reviewer report: Reviewer response for version 2

**DOI:** 10.21956/openreseurope.19288.r41570

**Published:** 2024-07-01

**Authors:** Juan Manuel Jiménez-Arenas

**Affiliations:** 1 Department of Prehistory and Archaeology, University of Granada, Granada, Spain

Dear author, I totally agree with your comments and reviews. So, I change my status of review report to accepted.

If any, are all the source data and materials underlying the results available?

Yes

Is the work original in terms of material and argument?

Partly

Is the work clearly and cogently presented?

Partly

Is the argument persuasive and supported by evidence?

Partly

Does the research article contribute to the cultural, historical, social understanding of the field?

Partly

Does it sufficiently engage with relevant methodologies and secondary literature on the topic?

Yes

Reviewer Expertise:

Archaeology; Prehistory; Palaeoecology; Human Evolution

I confirm that I have read this submission and believe that I have an appropriate level of expertise to confirm that it is of an acceptable scientific standard.

# Reviewer report: Reviewer response for version 2

**DOI:** 10.21956/openreseurope.19288.r41573

**Published:** 2024-06-24

**Authors:** Paolo Biagi

**Affiliations:** 1 Università Ca' Foscari Venezia, Venice, Italy

I do not have any further comments.

If any, are all the source data and materials underlying the results available?

Yes

Is the work original in terms of material and argument?

Yes

Is the work clearly and cogently presented?

Yes

Is the argument persuasive and supported by evidence?

Yes

Does the research article contribute to the cultural, historical, social understanding of the field?

Yes

Does it sufficiently engage with relevant methodologies and secondary literature on the topic?

Yes

Reviewer Expertise:

Prehistoric Archaeology, Lithic Studies

I confirm that I have read this submission and believe that I have an appropriate level of expertise to confirm that it is of an acceptable scientific standard.

# Reviewer report: Reviewer response for version 1

**DOI:** 10.21956/openreseurope.18390.r39212

**Published:** 2024-05-11

**Authors:** Paolo Biagi

**Affiliations:** 1 Università Ca' Foscari Venezia, Venice, Italy

During the last twenty-year, archaeologist have started to reconsider the results of old excavations, mainly those carried out during the last century, and propose new interpretations of the sequences and finds. Whenever possible, they have also started to redate the sequences by radiocarbon method. The results that have come out are sometimes controversial, sometimes different from those achieved by the excavators, sometimes difficult to interpret. The paper by J. Gennai considers the Mousterian sites and their related assemblages excavated in Tuscany mainly by the University of Pisa in the aforementioned period, presents and discusses this problem. Fortunately, many of the excavation plans, profiles and photographs of the sites and their sequences were available to the author, who has been capable to provide a good iconographic view of the caves and their locations as they were during those times and give the reader an idea of the excavation and retrieving techniques employed by the researchers and their related assemblages. It is also clear from some of the original hand drawings shown for the first time in the paper, how some of the sequences are unreliable (see figure 17), the way some others have been excavated in artificial spits of 5-10 cm (see figure 9 and 11), or the apparent homogeneity of the stratigraphic series. Following these premises, the task performed by the author to try to put some order into the Middle Palaeolithic cave sites of the region, has been very complicated, despite that fact that some of the authors of the old excavations are still active in the field and have been of some help in this respect. In my opinion, the most important merit of the paper is to have provided a clearer view of the old research, the way they were conducted, and to have published and interpreted in a reasonable way the original sequences and their descriptions, as they were shown by the old authors. The sites are undoubtedly very important within the general picture of the Middle Palaeolithic of the north Tyrrhenian Italian region. The documentation provided by the author is complete as regards both data and illustrations (photographs and line drawings). It can help interpret what was done in the recent past and the way the events that took place in those years, and the lithic artefacts have been reinterpreted. This is an important and systematically made task, without which it would be impossible to study the assemblages in a reliable way. I think that these are the most important aspects of the paper. There is no doubt that the some of the reinterpretations are somewhat weak due to the absence of important, original data and a reliable radiometric chronology of all sequences (see page 6 and 7 for example), and that another author might have achieved (slightly) different results. The procedures followed by the author are correct and complete as much as they could be. My general impression about the paper is positive.

If any, are all the source data and materials underlying the results available?

Yes

Is the work original in terms of material and argument?

Yes

Is the work clearly and cogently presented?

Yes

Is the argument persuasive and supported by evidence?

Yes

Does the research article contribute to the cultural, historical, social understanding of the field?

Yes

Does it sufficiently engage with relevant methodologies and secondary literature on the topic?

Yes

Reviewer Expertise:

Prehistoric Archaeology, Lithic Studies

I confirm that I have read this submission and believe that I have an appropriate level of expertise to confirm that it is of an acceptable scientific standard.

# Response

**Published:** 2024-05-23

**Authors:** Jacopo Gennai

**Affiliations:** Civilisations and Forms of Knowledge, University of Pisa, Pisa, Tuscany, Italy

I would like to thank the reviewer for the appreciation expressed.  As mentioned, the manuscript's main goal is to provide a re-assessment of the stratigraphy to provide a baseline for new investigations. I agree that the level of detail is too scant for modern requirements. At the same time, the sites represent the only evidence we currently have for this area and therefore I believe it is important to try to salvage the few information that could be still obtained.  For example, according to the new interpretation proposed radiocarbon dating has been performed and it will provide the first reliable chronological assessment of the Grotta del Capriolo and Buca della Iena deposits.

# Reviewer report: Reviewer response for version 1

**DOI:** 10.21956/openreseurope.18390.r39217

**Published:** 2024-05-11

**Authors:** Kévin Di Modica

**Affiliations:** 1 Scladina Cave Archaeological Centre, Espace Muséal d’Andenne, Andenne, Belgium

This paper is presenting evidence related to the Mousterian in Nort-Western Tuscany based on a reappraisal of excavation works done quite anciently, and not reaching the current standard of field research. I would first say this is an important work,

 I would first like to say that this is a considerable task, requiring significant work in collecting archives, and then integrating these archives - of various kinds - into a coherent system allowing for the interpretation of the site and its occupations. This type of meticulous work, which demands a lot of time, is not common but is nonetheless important, as the results obtained in terms of recontextualizing the material, with all its limitations, condition the interpretations of the material from the excavations and provide a solid foundation upon which future research can rely. In this regard, even if the results obtained do not appear particularly innovative or spectacular, this kind of work should be encouraged. 

 However, I note a difficulty in following the structure and objectives pursued by this article. The objectives and structure should appear more clearly at the beginning of the introduction. It is also not very clear that the article consists of a reassessment of data related to 3 specific sites, whereas the introductory chapter extensively covers several other sites, which are not dealt with subsequently. At the end of this introduction - or at the beginning of the "Methodology" chapter - it would be interesting to address the fact that the 3 sites treated in the article will provide complementary data to those detailed in the introduction.

 In the "Introduction" chapter, the stratigraphic data of the mentioned sites are quite complex, notably due to a long history of excavations. For readers not familiar with these sites, the text can be difficult to follow in the absence of graphical documents. Figures showing the locations of the various excavation campaigns on each site, as well as a summary in the form of a table of the stratigraphies of the different excavators, would be helpful.

 The "Methodology" chapter should be a bit longer. The types of data processed are explained clearly, but a paragraph detailing how these will be integrated and exploited is missing.

 The results are presented clearly.

 The discussion should be more in-depth. The impact of the results, for each site, is not apparent. To what extent has the revision of the documents affected the understanding of the site, the coherence of the assemblages, and their chronological positioning, for example?

 A more developed discussion would support the conclusion. Highlighting the contribution as well as the inherent limitations of this type of lithic assemblages when approached through this type of approach would better emphasize the importance of this kind of methodology. Such considerations would be more useful than others that are too general ("crucial insights into the last Neanderthal occupations…").

 To conclude this review on a positive note, I believe that some enhancements in the illustrations, improved text structuring, and a more thorough contextualization of the results and their limitations would better highlight the value of this text. It represents the culmination of significant historiographical efforts and underscores the necessity of such approaches to leverage, to the extent possible, ancient excavations within a modern research framework.

 In this regard, it serves as an example of a direction seldom taken by academic research, yet fundamental in the pursuit of optimizing the utilization of data from ancient excavations.

If any, are all the source data and materials underlying the results available?

Yes

Is the work original in terms of material and argument?

Partly

Is the work clearly and cogently presented?

Yes

Is the argument persuasive and supported by evidence?

Partly

Does the research article contribute to the cultural, historical, social understanding of the field?

Yes

Does it sufficiently engage with relevant methodologies and secondary literature on the topic?

Partly

Reviewer Expertise:

Middle Palaeolithic in NW Europe; Site stratigraphy; Old collections reappraisal; Lithic studies; Chronostratigraphy

I confirm that I have read this submission and believe that I have an appropriate level of expertise to confirm that it is of an acceptable scientific standard, however I have significant reservations, as outlined above.

# Response

**Published:** 2024-05-23

**Authors:** Jacopo Gennai

**Affiliations:** Civilisations and Forms of Knowledge, University of Pisa, Pisa, Tuscany, Italy

I would like to thank the reviewer for his appreciation and his suggestions. According to the comments, I improved the Introduction structure. Now it reports only the key information, I added some figures and I highlighted why it is important to include this information within the introductory section. I also added a Research Goal paragraph, so it would be easier for the reader to understand the manuscript goals. The methodology section has been improved including how the different sources have been used to produce the reassessment of each site. within the discussion and result sections I have made it clearer what are the new findings and how they depart from previously available information. I also provide a clearer re-assessment of the limitations of the sites according to the new interpretation. I hope that the new version meets the requested changes.

# Reviewer report: Reviewer response for version 1

**DOI:** 10.21956/openreseurope.18390.r38834

**Published:** 2024-05-11

**Authors:** Marko Banda

**Affiliations:** 1 Department of Archaeology, Faculty of Humanities and Social Sciences, University of Zagreb, Zagreb, Croatia

The article *The Mousterian in North-Western Tuscany: new data from the Piano di Mommio sites*, written by Jacopo Gennai, focuses on three Middle Palaeolithic sites in North-Western Tuscany - Buca della Iena, Grotta del Cervo and Grotta del Capriolo. It provides an exhaustive overview of the history of research and site information, including the stratigraphy and associated finds, according to the available field documentation, post-excavation reports and published local papers. This is done with the aim of providing a new interpretation of the stratigraphy for future analyses of the materials from the sites. 

 The contents and the arguments presented in the paper are original **.** While it does use documentation made by earlier researchers, it provides an original synthesis and is supported by photographs, drawings and plans made by the author themselves. The first part of the paper presents a wider discussion on the Middle Palaeolithic, especially related to the nature of lithic variability. The section after that features a historical synthesis of the research done on the Middle Palaeolithic in North-Western Tuscany. Thus, the article references the relevant secondary literature extensively, providing an overview of this archaeological phenomenon in this part of Italy from a mainly research history perspective. This part also points to the importance of studying the subject assemblages within the context of available stratified sites in Central Italy.

 The methodology of the paper is sound. It consists of a reassessment of the older documentation and supporting photographic documentation created during recent visits. The author uses the available information about sediment characteristics and the relative positions of arbitrary spits in order to reconstruct equivalent levels across the excavated areas. The resulting associations are convincing, and seem supported, insofar as the available original documentation is concerned. The sources are available both within the paper itself and in an open-access online repository that is frequently referenced throughout the paper.

 Therefore, the article provides a good example of how to reassess old field documentation in order to reconstruct the stratigraphy of previously excavated sites. Finally, it shows how this can enable the separation of the assemblages coming from those sites into relevant chronostratigraphic units. Because of that, the article has bearing not just on the discussions about the regional Middle Palaeolithic, but also on the wider issue of how to properly evaluate and utilize data from old excavations. This is particularly relevant in areas, such as the one in this paper, where old excavations account for a significant part, if not the majority, of the recovered evidence from stratified contexts.

 If any suggestion should be made, it would be that one more table could be added that would show the number of lithic finds according to the reconstructed levels. In my opinion, this would add to the readers' appreciation of the meticulous effort made by the author and would serve as a bridge to any future paper calling back to this publication. However, if this data is still unavailable, its absence would not invalidate the present paper.

 In addition, there are several minor suggestions that could improve the article, without requiring any substantial modification of the content. These are the following:

 1) Is it possible to add the spits from the Northern profile sectors A and B (1972-1973) from Buca della Iena to the correlation of the spits in Table 1? The author mentions 5 spits in the northern sector A and 4 in sector B. It would be interesting to see how these correlate to the spits in the other sectors, particularly because they also border the G. del Cervo excavation surfaces.

 2) A table of correlations for the spits in the various squares of Grotta del Cervo, such as the ones for B. della Iena and B. del Capriolo, is not provided. Since this site was excavated recently compared to the others (1989-1992), does this mean that the artefact provenience data and that the correlations between the spits in the various parts of the site are well documented in the original material? If this is the case, then the exclusion of such a table is justified. However, a sentence could be added to the section in the discussion about G. del Cervo that would state that explicitly.

 3) I would suggest that the title of the section "The Mousterian sites in Tuscany" rather be "The Mousterian sites in **North-Western** Tuscany". That way, any confusion could be avoided, as one may reasonably object to the exclusion of other important Tuscan sites from this overview, such as Grotta dei Santi (Moroni et al. 2019) or Grotta La Fabbrica (Villa et al. 2018).

 4) Finally, the text could be checked for some minor grammar or vocabulary improvements. Also included are some of the minor errors found throughout the text, including the page number referring to the PDF version:

 p. 6 - " **Registrar**" should perhaps be changed to " **Register**" or " **Registry**" as the first word refers to a person.

 p. 11 - Instead of "the **sedimenti is changing**" it should be something along the lines of "the **sediment is different in terms of**".

 p. 11 - "...thick brown loamy **with a gravel layer**" should rather say "...thick brown loamy **layer with gravel**"

 p. 11 - "...and the **progressing** hardening of the sediment" should be changed to " **progressive **hardening of the sediment"

 p. 11 - In the sentence "In 1968, samples of the flowstone were radiometrically dated with the Th230/U230 and gave two dates (< 41000 and < 51000 years), the discrepancy **of which **is due to the high content of clay in the flowstone" the bolded words should be added.

 p. 12 - "big **stones** layer" should rather say "big **stone** layer"

 p- 12/13 - In the sentence "...only at -2.80 m (spit 36) depth did the sediments **hardened **and were preserved" should be replaced with " **harden**" or the sentence should be changed to: "...only at -2.80 m (spit 36) depth **were **the sediments hardened and preserved."

 p. 13 - "...by the “Alberto Carlo Blanc” archaeological

**Group** (Radmilli, 1968)" - Maybe it is not necessary to capitalize the word " **group**".

 p. 14 - In the sentence "The deposit was excavated in arbitrary **spit**..." the last word should be in plural: " **spits**"

If any, are all the source data and materials underlying the results available?

Yes

Is the work original in terms of material and argument?

Yes

Is the work clearly and cogently presented?

Yes

Is the argument persuasive and supported by evidence?

Yes

Does the research article contribute to the cultural, historical, social understanding of the field?

Yes

Does it sufficiently engage with relevant methodologies and secondary literature on the topic?

Yes

Reviewer Expertise:

My area of research is the Middle Palaeolithic, in particular the lithic technology from this period. However, I have also done some research on Middle Palaeolithic bone tools (retouchers), Late Pleistocene palaeoenvironment and Neanderthal ecology and radiometric dating. My work has included assemblages from sites which were excavated in the mid-20th century which have contextual problems associated with insufficient documentation and substandard methodology. In geographic terms, I have worked on both the Central European and Mediterranean Middle Palaeolithic. However, I am less familiar with the western coast of Italy when compared to the eastern (Adriatic) coast and its hinterland.

I confirm that I have read this submission and believe that I have an appropriate level of expertise to confirm that it is of an acceptable scientific standard.

## References

[1] : Late Neandertals in central Italy. High-resolution chronicles from Grotta dei Santi (Monte Argentario - Tuscany). *Quaternary Science Reviews* .2019;217: 10.1016/j.quascirev.2018.11.021 130-151 10.1016/j.quascirev.2018.11.021

[2] : From Neandertals to modern humans: New data on the Uluzzian. *PLoS One* .2018;13(5) : 10.1371/journal.pone.0196786 e0196786 10.1371/journal.pone.0196786 29742147 PMC5942857

# Response

**Published:** 2024-05-23

**Authors:** Jacopo Gennai

**Affiliations:** Civilisations and Forms of Knowledge, University of Pisa, Pisa, Tuscany, Italy

I would like to thank the reviewer for the appreciation showed to the manuscript and its goals. I have accepted all the reviewer's suggestions: - Now I provide a numerical reassessment of lithic assemblages. Nevertheless, I plan a more detailed publication on lithic techno-typological aspects. - I provide a better discussion of the Buca della Iena 1972-73 campaign and its findings. - I include a sentence highlighting that Grotta del Cervo deposit is assumed to be subhorizontal, even though some caution should be exerted about the upper part of the sequence as the original excavator pointed out some disturbance. Even though she does not provide any explanation of the process.   - Now the paragraph title correctly reports "North-Western".  - I thank the reviewer for spotting typos, which I promptly corrected

# Reviewer report: Reviewer response for version 1

**DOI:** 10.21956/openreseurope.18390.r39213

**Published:** 2024-04-22

**Authors:** Juan Manuel Jiménez-Arenas

**Affiliations:** 1 Department of Prehistory and Archaeology, University of Granada, Granada, Spain

First of all, I would like to express my gratitude for the opportunity to review this manuscript. It is a necessary task as a preliminary step to bring order to the dispersed and sometimes unsystematic results derived from ancient excavations, in this case  the Middle/Upper Palaeolithc sites of Buca de la Iena, Grotta del Cervo, and Grotta del Capreolo (Tuscany, Italian Peninsula). This is a common place in archaeology, applicable to other geographical contexts as well, such as the Iberian Peninsula (e.g., Solana del Zamborino, Fonelas, Granada, southern Spain: Botella et al. 1976). Therefore, this type of study is fully justified.

 As the author points out, it is necessary to gather the scattered information published in local media that is difficult to access for the scientific community at large. However, it would be expected in a work classified as a "Research Article" to provide some substantial novelty to the discussion on Neanderthal/AMH settlement in this region of the Italian Peninsula based on three sites: Buca de la Iena, Grotta del Cervo, and Grotta del Capreolo.

 However, as a manuscript reviewer, it is not clear to me what the author's objectives. And that is fundamental. Knowing where the work is heading is informative for both the author and the readers. My suggestion, therefore, is for the author to make them explicit. An appropriate place could be, for example, at the end of the introductory section. Furthermore, the author should consider that outlining the objectives should lead to a restructuring of their work, especially the results and conclusions, taking into account the "horizons" towards which this work is directed.

 The sections dedicated to the introduction and results are very interesting as a state of the art for an article that includes interpretative novelties contributing to the debate on human occupation during the Middle Paleolithic and Upper Paleolithic, as well as their transition in Tuscany (Italian Peninsula).

 Regarding the conclusions, the author highlights that compiling previously generated documentation, along with their effort to compare it with what can still be seen in the field, contributes to a paradigm shift. If we consider Thomas Kuhn's proposal (1970) for such a process to occur, there must be changes in theories, instruments, values, and/or metaphysical assumptions leading to a new "normal science." This brings me to a recurring theme throughout the article, the writing style. I believe the author should avoid using qualifying adjectives that exaggerate the narrative.

 Regarding the relationship between stratigraphic position and relative age, it is relevant to point out that although, in general terms, the oldest layers tend to be at a lower position, not everything deeper is necessarily older. Care must be taken with such statements when made uncritically, as post-depositional processes may undergo reworking. This is especially critical when dealing with ancient excavations where information on the excavation process and taphonomic information is either nonexistent or scarce.

 Finally, please avoid the use of absolute datings and opt for numerical datings or radiometric datings.

If any, are all the source data and materials underlying the results available?

Yes

Is the work original in terms of material and argument?

Partly

Is the work clearly and cogently presented?

Partly

Is the argument persuasive and supported by evidence?

Partly

Does the research article contribute to the cultural, historical, social understanding of the field?

Partly

Does it sufficiently engage with relevant methodologies and secondary literature on the topic?

Yes

Reviewer Expertise:

Archaeology; Prehistory; Palaeoecology; Human Evolution

I confirm that I have read this submission and believe that I have an appropriate level of expertise to state that I do not consider it to be of an acceptable scientific standard, for reasons outlined above.

# Response

**Published:** 2024-05-23

**Authors:** Jacopo Gennai

**Affiliations:** Civilisations and Forms of Knowledge, University of Pisa, Pisa, Tuscany, Italy

I would like to thank the reviewer for his appreciation on my effort to make widely available unpublished and partially published stratigraphical and research history details about the Buca della Iena, Grotta del Capriolo, and Grotta del Cervo sites. I also thank you for the constructive criticisms, which led to the improved new version of the manuscript. the first critical point addressed whether my manuscript brought enough novel information to be a research article. I believe that the problem highlighted by the reviewer lies in the article title and the expectations ensuing it. The new title specifies that the previously unpublished information is the new information. These details have never been published and led me to a new stratigraphical reassessment of at least Buca della Iena and Grotta del Capriolo. Through the newly published details, I provide a new interpretation and reassessment of the sites, their intra and inter-correlations and whether they can be reliably included in new investigations about the Neanderthal presence in North-Western Tuscany. I think that the manuscript qualifies as a research article, according to the Open Research Europe definition.  The second critical point outlined that the manuscript's objectives were not clear enough. I accepted the reviewer's suggestion and included a Research Objectives paragraph. Also, I made it clearer throughout the text what information was available in the previously published works and what is my contribution to the new understanding of the sites.  I think the third critical point ("no paradigm shift") is partly due to the two previous ones. The reviewer expected new data about the Middle to Upper Palaeolithic Transition and rightly pointed out the absence. Now I removed the sentence and I think it is clearer that the paradigm shift I was referring to was the new stratigraphical interpretation and sites' reassessment.  The fourth critical point sounds more like a general statement, which I agree with in principle. Nevertheless, I fail to understand how and where I would have assumed the integrity of the sites. In the new version, I took care of highlighting what I believe is in situ and what part of the sites I reasonably interpret as affected by post-depositional processes.  The fifth critical point is also difficult to understand. I believe the reviewer hinted at the use of Marine Isotope Stages (MISs) chronology. I would like to point out that using MIS is an accepted absolute dating practice in prehistoric archaeology, especially when defining broad evolutionary events that do not have a punctual enough date (for example, the introduction of the Levallois concept in lithic technology). Nevertheless, as MIS can be provided with their numerical age span, I now added those for the benefit of the reader.  a final criticism has been voiced about the writing style. I reduced the number of qualifying adjectives when it was not necessary.
